# ﻿Taxonomic revision and phylogenetic relationships of *Thinouia* (Sapindaceae), a neotropical genus

**DOI:** 10.3897/phytokeys.252.129621

**Published:** 2025-02-20

**Authors:** Herison Medeiros, Pedro Acevedo-Rodríguez, Jenifer de Carvalho Lopes, Rafaela Campostrini Forzza

**Affiliations:** 1 Instituto de Pesquisas Jardim Botânico do Rio de Janeiro, Pacheco Leão, 915, 22460-030, Rio de Janeiro, Rio de Janeiro, Brazil Instituto de Pesquisas Jardim Botânico do Rio de Janeiro Rio de Janeiro Brazil; 2 Universidade de São Paulo, Instituto de Biociências, Departamento de Botânica, Rua do Matão, 277, 05508-090 São Paulo, SP, Brazil Universidade de São Paulo São Paulo Brazil; 3 Department of Botany, MRC-166 Smithsonian Institution, P.O. Box 37012, Washington D.C. 20013-7012, USA Smithsonian Institution Washington DC United States of America; 4 Department of Organismic & Evolutionary Biology, Harvard University Herbaria, 22 Divinity Avenue, Cambridge, MA 02138, USA Harvard University Herbaria Cambridge United States of America; 5 Instituto Chico Mendes de Conservação da Biodiversidade, ICMBio, Prado, Bahia, Brazil Instituto Chico Mendes de Conservação da Biodiversidade Prado Brazil

**Keywords:** Lianas, Neotropical flora, Paullinieae, Sapindales, Sapindoideae

## Abstract

A taxonomic revision of *Thinouia* (Sapindaceae) is presented, including typification and descriptions for all accepted species. Phylogenetic analyses based on molecular data confirm that the genus is monophyletic and suggest two main clades with *T.cazumbensis* sister to the other species of the genus. Thirteen species of *Thinouia* are accepted, including a new species from Amazonia. The genus is distributed across continental tropical America, with its highest diversity found in South America. Its species can be distinguished by morphological details of leaves, indumentum, inflorescences, flowers, and fruits. We present a comprehensive review of the morphology and geographical distribution of the genus, along with an identification key, distribution maps, conservation risk assessments, illustrations, and comments on the ecology and taxonomy for all species, gathered from the literature and fieldwork.

## ﻿Introduction

*Thinouia* Triana & Planch. is a small genus of Neotropical lianas, comprising 13 species distributed from southern Mexico to southern Brazil and Argentina, across a wide variety of habitats including savannas, evergreen forests and rainforests in the Neotropical region (Ferrucci and Somner 2008; Acevedo-Rodríguez et al. 2011; Medeiros et al. 2020). The genus is monophyletic and currently is placed in tribe Paullinieae, alongside *Cardiospermum* L., *Lophostigma* Radlk., *Paullinia* L., *Serjania* Plum. ex Miller, and *Urvillea* Kunth (Acevedo-Rodríguez et al. 2017; Medeiros et al. 2020). *Thinouia* is characterized by the presence of umbelliform and racemiform thyrses, actinomorphic flowers with marginal or bifid petal appendages, isopolar tricolporate pollen grains, and schizocarpic fruits that split into three mericarps, each with a distal wing (Ferrucci and Somner 2008; Acevedo-Rodríguez et al. 2017; Medeiros et al. 2020).

After extensive work in the field, herbarium, and molecular laboratory (Acevedo-Rodríguez et al. 2017; Medeiros et al. 2020), we present an updated taxonomic revision of *Thinouia* based on novel molecular phylogenetic data. Since the last taxonomic revision of the genus (Radlkofer 1931–1934), almost 100 years ago, new species have been described; more information regarding distribution, morphology and ecology have been gathered; and the phylogenetic relationships among the species have been analyzed. In this revision, we provide morphological descriptions for all taxa, along with an identification key, illustrations, distribution maps, conservation risk assessments, and comments on their ecology, nomenclature and taxonomy.

## ﻿Material and methods

### ﻿Taxonomy

Morphological descriptions and phenology of species are based on fieldwork observations gathered by the senior author, and on the study of herbarium specimens deposited at the following herbaria: ALCB, BHCB, CEPEC, COL CVRD, ESA, F, FUEL, GH, HCF, HSTM, HUEFS, IAN, ICN, INPA, F, JPB, K, NY, MBM, MBML, MEDEL, MEXU, MG, MICH, MO, P, QCNE, R, RB, RBR, RON, SP, SPF, U, UB, UFACPZ, UPCB, USM, US, and VIES (according to Thiers 2022). Our analyses included the samples deposited in the Laboratory of Botany and Plant Ecology of the Federal University of Acre (LABEV). The indumentum terminology follows Beentje (2010), leaf and fruit terminology follow Radford et al. (1974), leaf venation terminology follows Ellis et al. (2009), and inflorescence terminology follows Weberling (1989). The species concept follows the evolutionary species concept of de Queiroz (2007) and we considered isolated evolutionary lineages diagnosed by morphological characters to be separate species.

Maps were elaborated using ArcGIS 10.5 software (ESRI 2016), geographical coordinates were obtained from herbaria specimens, and shapefiles were obtained from The Americas Base Map (Bletter et al. 2004) and WWF (2022). The distribution of *Thinouia* across biomes in the Neotropics is based on Olson et al. (2001). For each species, one representative specimen per municipality is cited. An Index to Numbered Collections Studied is included in Appendix 1.

The conservation status for each species was evaluated according to criteria adopted by the International Union for Conservation of Nature (IUCN 2022). For *Thinouiatomocarpa* and *T.silveirae* the extent of occurrence (EOO) and area of occupancy (AOO) were calculated using Kew’s GeoCAT tool (available at: <https://geocat.iucnredlist.org/>) (Bachman et al. 2011). For the other species, the EOO and AOO were calculated in collaboration with the CNCFlora (available at: <http://www.cncflora.jbrj.gov.br/portal>), adopting EOO for the weedy species and AOO, based on 2 km cell width, for the remaining species. Collections that could not be properly georeferenced were excluded from EOO and AOO calculations.

### ﻿Phylogenetic reconstruction

The phylogenetic analysis included the same markers as in Acevedo-Rodríguez et al. (2017) and Medeiros et al. (2020): the plastid marker trnL intron and nuclear ribosomal internal transcribed spacer (ITS). The genomic DNA was extracted using DNA NucleoSpin Plant II kit (Machery-Nagel, GmbH & Co. KG, Dueren, Germany) following the manufacturer’s protocol. Approximately 60 mg of leaf tissue were pulverized with Tissuelyzer (Qiagen, Dusseldorf, Germany) for 3 min at 60 hz. PCR amplification used the primers and the protocols described in Acevedo-Rodríguez et al. (2017). PCR products were purified and sequenced by Macrogen (Seoul, South Korea). All sequences, vouchers and GenBank accession numbers are summarized in Appendix 2.

The alignments were performed using Muscle (Edgar 2004) using the default parameters implemented in Geneious software (Kearse et al. 2012). Poorly aligned regions were manually adjusted and removed in case they could not unambiguously be aligned. We used jModelTest 2.0 (Guindon et al. 2010; Darriba et al. 2012) and the Akaike information criterion (AIC) to select the best-fit model of nucleotide substitution for each dataset. GTR+I+G was selected as the best model for the ITS dataset, whereas HKY+G was selected as the best model for the trnL dataset. Bayesian Inference (BI) analyses were conducted using MrBayes 3.2.2 (Ronquist et al. 2012) in the online CIPRES Science Gateway interface (Miller et al. 2015) with four Markov chain Monte Carlo (MCMC) runs using a random starting tree and 10 million generations, with a sampling frequency of one every 1000^th^ generations. We used Tracer 1.7 (Rambaut et al. 2018) to check for convergence of the MCMC and to check for stationarity. We discarded 25% of the trees as burn-in.

Since the morphology of petal appendages was an important character in Radlkofer’s infrageneric classification of the genus (1878), we performed an ancestral character state reconstruction analysis of this structure with Mesquite v. 3.61 (Maddison and Maddison 2019), to demonstrate if Radkofer’s classification is good. We analyzed the size attributes of the petal appendages (longer than the petals vs. shorter or equal to the petals) using two complementary approaches, parsimony of ancestral states, and likelihood of ancestral states. For the likelihood of ancestral states method, the current probability model was selected.

## ﻿Results

### ﻿Taxonomic history

*Thinouia* was described by Triana and Planchon (1862) based upon *Thouiniascandens* Cambess., a species that they transferred into a new genus, *Thinouia*. Although no explanation is given by Triana and Planchon for the origin of the name *Thinouia*, it seems to be an anagram of *Thouinia*, a genus that honors André Thouin [1747–1824] a distinguished French botanist. The genus wasn’t immediately recognized by contemporaries of Triana & Planchon, e.g., Bentham and Hooker (1862) or Baillon (1874), until Radlkofer [1829–1927], the acclaimed specialist of Sapindaceae, published a new species and a synopsis of the genus in 1878, when he recognized seven species. In his treatment of Sapindaceae for Martius’ "Flora Brasiliensis" (Radlkofer 1892–1900), Radlkofer monographed *Thinouia*, recognizing 11 species. In his posthumous publication of Sapindaceae for Engler’s "Das Pflanzenreich" (1931–1934), his treatment of *Thinouia* was published verbatim from his treatment for "Flora Brasiliensis".

Radlkofer (1878) organized the genus in two sections. Thinouiasect.Lepidodine (= sect. Thinouia), presented a single species, *T.myriantha*, and was characterized by the presence of a petaloid appendage that is longer than the petals; Thinouiasect.Petalodine, with five species, was characterized by a petaloid appendage that is shorter than the petals. *Thinouiaobliqua* Radlk. wasn’t classified in this scheme because its flowers were unknown at the time. In 1890, recognizing the affinity of *Thinouia* with other genera of climbing Sapindaceae, Radlkofer placed *Thinouia* in a monotypic subtribe (Thinouieae) within tribe Paullinieae, which in turn was placed in his series Eusapindaceae (= subfam. Sapindoideae). In his 1931–1934 monograph, Radlkofer presented a brief taxonomic history of the genus and recognized the following 11 species: *Thinouiacompressa* Radlk., *T.coriacea* Radlk., *T.mucronata* Radlk., *T.myriantha* Triana & Planch., *T.obliqua* Radlk., *T.paraguayensis* (Britton) Radlk., *T.repanda* Radlk., *T.sepium* S. Moore, *T.scandens* (Cambess.) Triana & Planch., *T.ternata* Radlk., and *T.ventricosa* Radlk.

Following Radlkofer’s work, few changes have taken place in the taxonomy of the genus. Based on the striking morphology of the fruits, Standley and Record (1936) described *Thinouiatomocarpa* from British Honduras (now = Belize), a discovery that expanded the known occurrence of the genus outside South America. Croat (1976) recorded *T.myriantha* from Panama while placing *T.tomocarpa* as a synonym of this species. In your work de la Cruz and Dirzo (1987) recorded *T.tomocarpa* for Veracruz, Mexico. Ferrucci (1991), in her treatment of Sapindaceae for the "Flora de Paraguay", recorded the distribution of *T.compressa* and *T.mucronata* for Paraguay for the first time and synonymized *T.repanda* under *T.mucronata* and *T.sepium* under *T.paraguayensis*.

While revising Serjaniasect.Platycoccus, and based on a morphological-based cladistic analysis, Acevedo-Rodríguez (1993) proposed *Thinouia* as a member of tribe Cupanieae, in the vicinity of *Allosanthus* Radlk. This placement was later reversed, and *Allosanthus* was placed in synonymy with *Thinouia* (Acevedo-Rodríguez et al. 2011), a decision that is supported by recent molecular studies (Acevedo-Rodríguez et al. 2017, Medeiros et al. 2020). Ferrucci and Somner (2008) described *Thinouiarestingae* Ferrucci & Somner, a species endemic to SE Brazil and placed in section Petalodine. They also presented a key to the species of *Thinouia* occurring in Brazil. In 2020, *T.cazumbensis* H. Medeiros was described from the state of Acre, Brazil (Medeiros et al. 2020).

### ﻿Distribution and ecology

**Figure 1.**
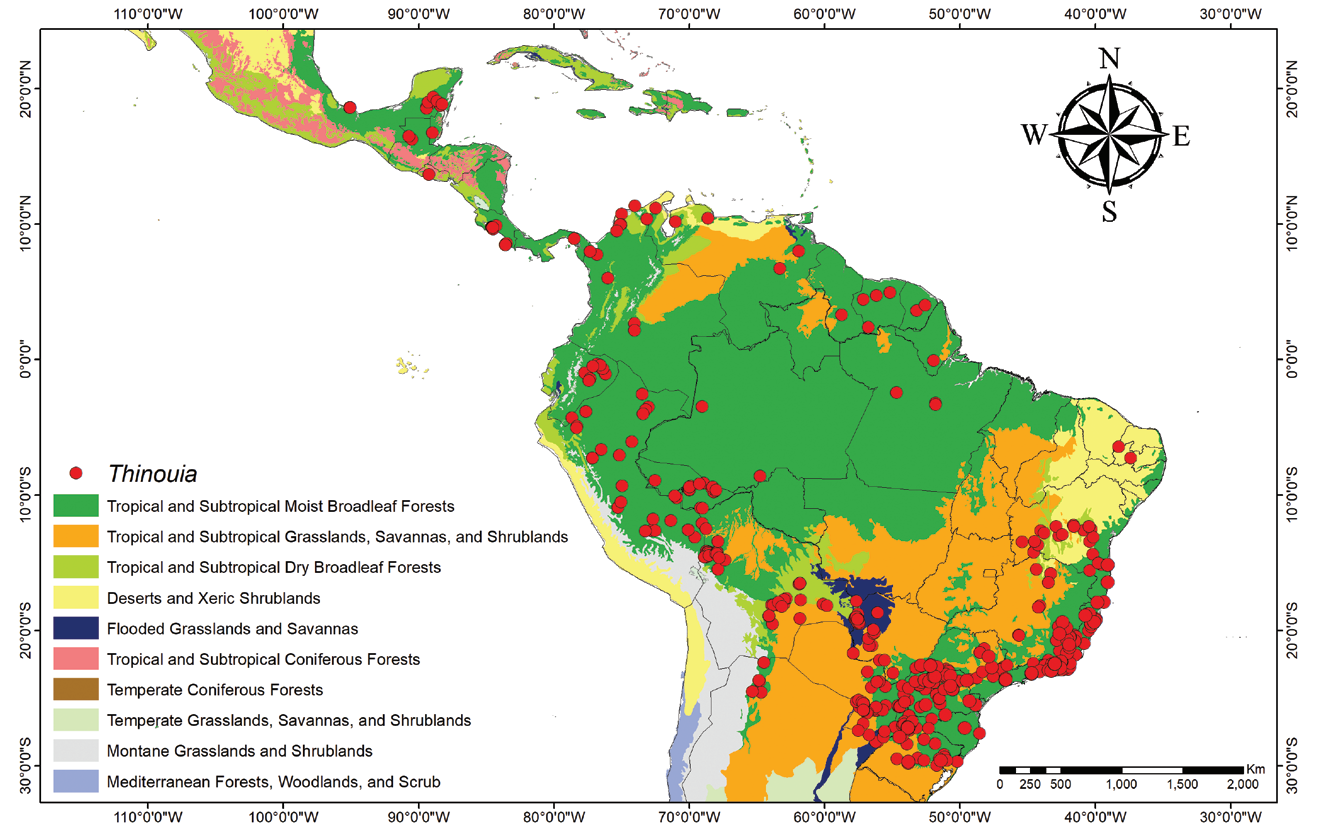
Distribution of *Thinouia* across biomes in the Neotropics. Shapefile WWF 2022 (Olson et al. 2001).

*Thinouia* is a relatively small genus of Neotropical lianas comprising 13 species distributed from southern Mexico to southern Brazil and Argentina, across a wide variety of habitats, including savannas, evergreen forests and rainforests in the Amazon, Central America, and Brazilian Atlantic Forest (Fig. 1). The occurrence of *Thinouia* in Central America and on the Guiana Shield is almost entirely due to two species (*T.trifoliolata* and *T.myriantha*) that are widely distributed, and in southern Mexico it is represented by only one species (*T.tomocarpa*). The Amazon region and the Brazilian Atlantic Forest are major centers of diversity for the genus with four and eight species, respectively. Furthermore, some species also occur in cerrado vegetation, in gallery or seasonally dry forests.

### ﻿Morphology

#### ﻿Life form, stem morphology and macroanatomy

All species of *Thinouia* are tendrilled lianas that often reach the canopy of the forest. However, in the absence of phorophytes, some species may grow as arching shrubs.

**Figure 2.**
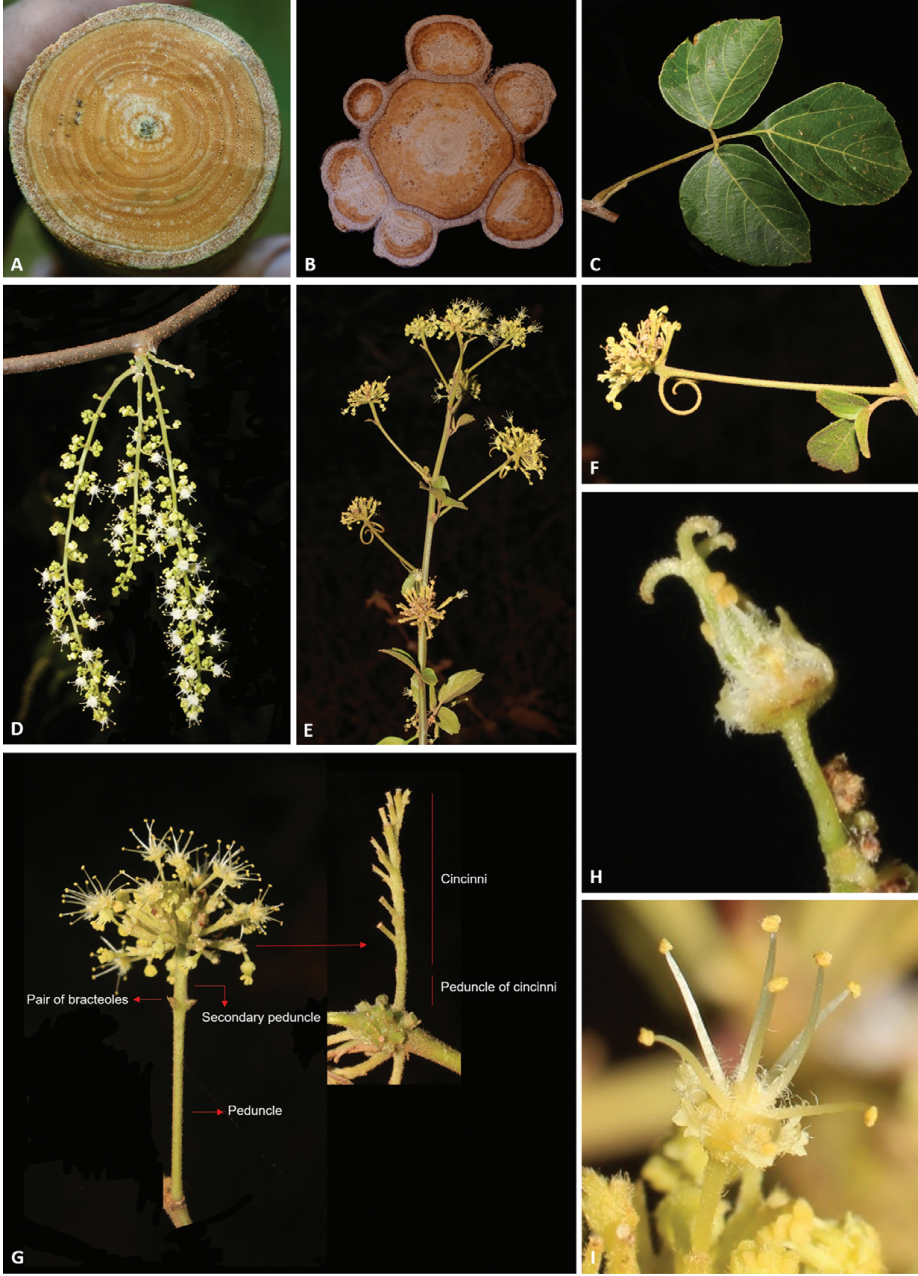
**A** Cylindrical simple stem in *Thinouiaparaguayensis***B** lobed stem with a central vascular cylinder and six neoformed peripheral ones in *T.scandens***C** trifoliolate leaf of *T.obliqua***D** cauliflorous inflorescence in *T.trifoliolata***E** terminal frondobracteate synflorescence in *T.silveirae***F** umbelliform, long-peduncled thyrse with tendrils in *T.silveirae***G** diagram of an umbelliform inflorescence with terminology **H** pistillate flower **I** staminate flower [Acevedo-Rodríguez 16750 (**A**) 3701 (**B**); Medeiros 3832 (**C**) 3331 (**D**) 4496 (**E–G**, **I**) 4464 (**H**); photos: **A, B** by P. Acevedo-Rodríguez **C–I** by H. Medeiros].

Stems are woody, cylindrical (Fig. 2A) or ribbed (Fig. 2B), reaching up to ca. 13 cm in diam. and 15 m in length (e.g., *Thinouiascandens*). During initial stages and early secondary growth, stems in all species have a regular anatomy (here termed simple, Fig. 2A) that is produced by the activity of a single, continuous cambium. This regular anatomy continues through the plant’s entire lifespan in some species (e.g., *Thinouiacazumbensis*, *T.compressa*, *T.myriantha*), but in a number of species (e.g., *T.obliqua*, *T.restingae*, *T.scandens*), secondary vascular cylinders develop from new cambia within the pericycle during late secondary growth. These secondary vascular cylinders (= peripheral cylinders), which aren’t connected to the initial vascular cylinder (= central cylinder), grow in thickness in a manner similar to the one described for the central cylinder (Tamaio and Somner 2010). The combination of these vascular cylinders form a cable-like structure, where some of the peripheral cylinders project as ribs along the external surface of the stem (Fig. 2B). The peripheral cylinders (5–7 larger ones and numerous smaller ones), are produced continuously, and in a cross-section of the stem are shown as having diameters that relate to their developmental stages (Fig. 2B). This anatomical variant is referred to by Tamaio and Somner (2010) as the corded vascular system and by Angyalossy et al. (2015) as neoformed secondary vascular cylinders (neo formations).

*Thinouia* may be confused with woody species from the closely related genera *Paullinia* or *Serjania* but may be distinguished from these by the presence of neoformed secondary vascular cylinders. These are characterized by the presence of sclereids in the center, instead of a pith as in the peripheral cylinders in species of *Paullinia* and *Serjania* (Tamaio and Somner 2010, Cunha Neto et al. 2017). The latter two genera may have neoformed secondary vascular cylinders, but these are formed symmetrically around the central cylinder instead of randomly, as in *Thinouia* (Acevedo-Rodríguez et al. in press).

#### ﻿Stipules, leaves, trichomes and indumentum

The shape of stipules seems to be a conserved character as all species show minute, triangular or deltate stipules, axillary .

Leaves in all species are trifoliolate (Fig. 2C), with the terminal leaflet consistently different from the lateral ones. Leaflets are elliptic to obovate, acute, decurrent, subcuneate, truncate, obtuse, or rounded at the base, and acute, acuminate, rounded, emarginate or obtuse and frequently mucronate at the apex. Most species have subtriplinerved venation.

The indumentum is variable regarding both type (simple, arachnoid or capitate) and density (glabrous to tomentose) of trichomes. Indumentum variation in the cavity of the seed locule is of great utility in distinguishing different species.

#### ﻿Inflorescences and flowers

Inflorescences in *Thinouia* are umbelliform (Fig. 2E) or racemiform thyrses (Fig. 2D) often bearing a pair of circinate tendrils at the apex of the peduncle (Fig. 2F), which always bears a diminute bract at base of the peduncle and two bracteoles at the apex or at the base of the secondary peduncle (Fig. 2G). Thyrses are axillary and simple, or terminal and forming a synflorescence. Additionally, *T.trifoliolata* may be cauliflorous (Fig. 2D). Flowers are grouped in cincinni (Fig. 2G).

*Thinouia* flowers are actinomorphic (Fig. 2I) and do not show much variation across species, with the exception of *T.obliqua* where the number of stamens is 6–7 (instead of 8, like in the other species). Flower size typically ranges between 2 to 5 mm long. Sepals are variable in terms of pubescence and shape, but normally they are abaxially pubescent and adaxially glabrous.

The corolla consists of five free petals, mostly spatulate or less frequently lanceolate, deltoid or obdeltate. Petals are accompanied by petaloid appendages that are either adnate to their adaxial basal surface or are a prolongation of the petal’s margin. The appendages are appressed against the filaments in a way that seems to restrict access to the nectary disc. They are bilaterally symmetrical, sometimes branched distally, and vary in size, being either shorter or slightly, to significantly, longer than the petals.

The androecium consists of 8 stamens (6–7 in *Thinouiaobliqua*) with basally connate filaments of equal length, which are usually white or cream. Stamens are 1.5 to 4.8 mm long (from the base of the united filaments to the anther apex). The anthers are yellow, ca. 0.5 mm long, ellipsoid, dorsifixed, and introrse, opening by longitudinal slits; they are glabrous, glandular or villous (Fig. 2I). Pollen grains are isopolar, obtusely triangular in polar view and subspherical in equatorial view, tricolporate, with elongate colpi nearly reaching the poles, and striate (Ferrucci and Anzotegui 1993; Acevedo-Rodríguez et al. 2017). The gynoecium is superior, syncarpic and tricarpellate; the ovary in most species is pubescent and the style is usually longer than the three papillose and terete stigmata (Fig. 2H); the ovules are solitary with axial placentation.

#### ﻿Fruits, seed and embryo

**Figure 3.**
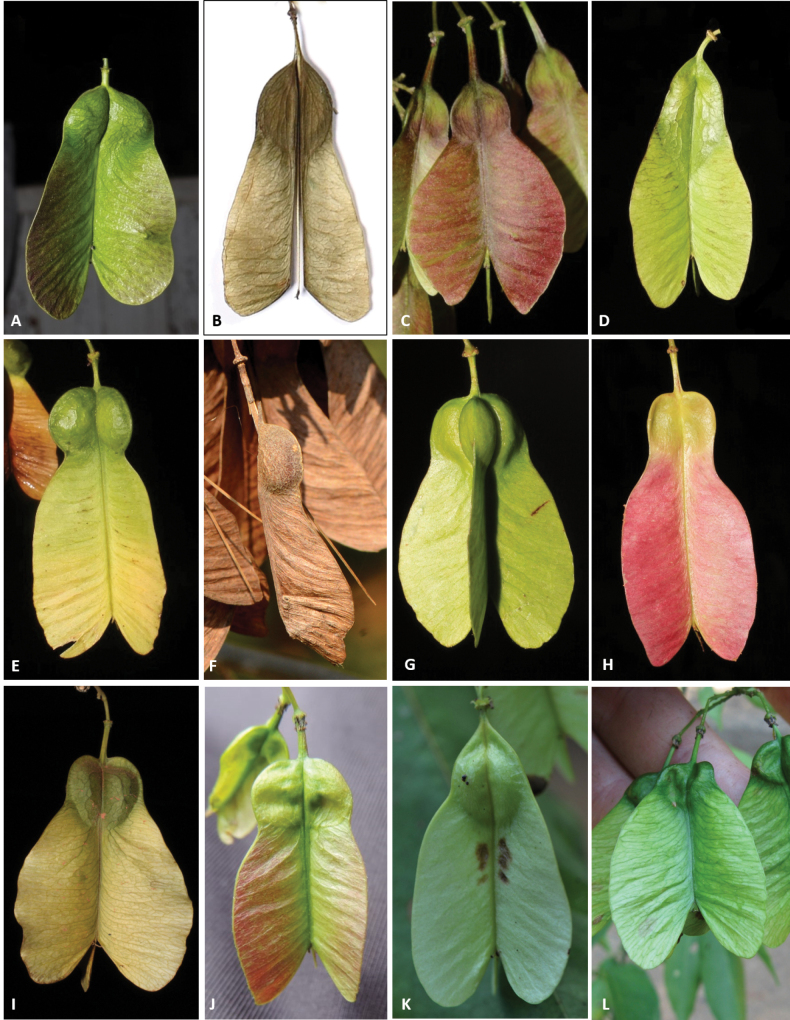
Color and shapes of schizocarps in *Thinouia*. **A***T.cazumbensis***B***T.compressa***C***T.mucronata***D***T.myriantha***E***T.obliqua***F***T.paraguayensis***G***T.restingae***H***T.scandens***I***T.silveirae***J***T.ternata***K***T.trifoliolata***L***T.ventricosa* [Acevedo-Rodríguez 16750 (**F**) 17159 (**K**); Cálio 70 (**B**); Daneu 746 (**J**); Ferrucci s. n.. (**L**); Figueira 927 (**C**); Medeiros 3401 (**A**) 3330 (**D**) 3832 (**E**) 4453 (**G**) 4473 (**H**) 2191 (**I**); photos: **F, K** by P. Acevedo-Rodríguez; J by L. Daneu **L** by M. S. Ferrucci **C** by M. Figueira **A, B, D, E, G, H, I** by H. Medeiros].

The fruit in *Thinouia* is a stipitate schizocarp that splits into three mericarps, each with a distal wing and a proximal locule where the seed is located (Fig. 3). Young and not fully mature fruits are often greenish or reddish rose, turning straw-colored when mature (Fig. 3F). Mericarp size varies from 3 to 7.5 cm in length, but there is little variation in shape between species (Fig. 3). The locule is consistently subglobose or lenticular although horizontally flattened in *T.compressa* (Fig. 3B), and in *T.myriantha* (Fig. 3D) the wing is distal but follows the locule along the dorsal margin; however, in some species the wing is not formed on the dorsal margin of the locule, forming an indentation on that region (Fig. 3C, E, H). The pericarp is glabrous or less often puberulous to pubescent. The cavity of the seed locule is glabrous or pubescent. Seeds are trigonous-ellipsoid or lenticular-ellipsoid, exarillate, with a small hilum (Acevedo-Rodríguez et al. 2017). The embryo is consistently ovoid, showing folded cotyledons. 2n = 28 (Urdampilleta et al. 2008).

### ﻿Phylogenetic relationships

**Figure 4.**
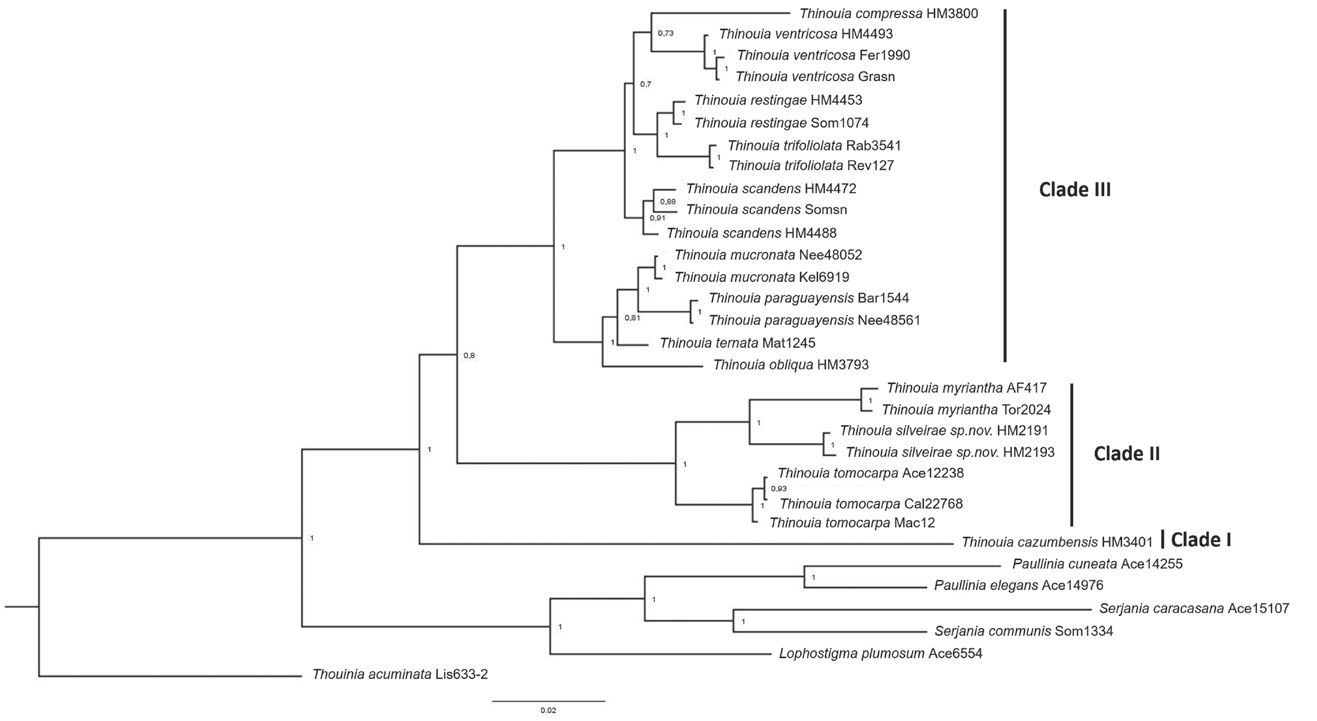
Maximum clade credibility tree from a Bayesian analysis of the combined two-marker dataset for *Thinouia* and outgroups. Bayesian posterior probability values are indicated above the branches.

The ITS dataset included 31 terminals and the alignment had a length of 781 bp; the trnL dataset included 31 terminals and was 616 bp long; the combined dataset included 31 terminals and had a length of 1398 bp. Only the topology from the combined analysis is described here (Fig. 4), as our separate analyses of each locus did not reveal any strongly supported incongruences.

Based on Acevedo-Rodríguez et al. (2017) and Medeiros et al. (2020), the monophyly of *Thinouia* is strongly supported and the group is recovered as sister to the other genera in tribe Paullinieae. In the current phylogenetic reconstruction of *Thinouia* (Fig. 4), we analyzed all 13 species of the genus, nine of which are represented by more than one accession. In all instances, the species form a single monophyletic lineage. *Thinouiacazumbensis* was recovered as sister to a clade containing the remaining species of the genus and with a high posterior probability (PP = 0.8). Within this group there were two clades, here called Clade II and Clade III. Clade II is strongly supported (PP = 1) and includes *T.tomocarpa* as sister to *T.myriantha* and the new species *T.silveirae*. Clade III has strong support (PP = 1), and contains two subclades; the first subclade included *T.obliqua*, successively followed by *T.ternata*, and *T.paraguayensis* + *T.mucronata*. The second subclade included *T.scandens* as sister to a group formed by *T.trifoliolata*, *T.restingae*, *T.compressa*, and *T.ventricosa* (PP = 1).

**Figure 5.**
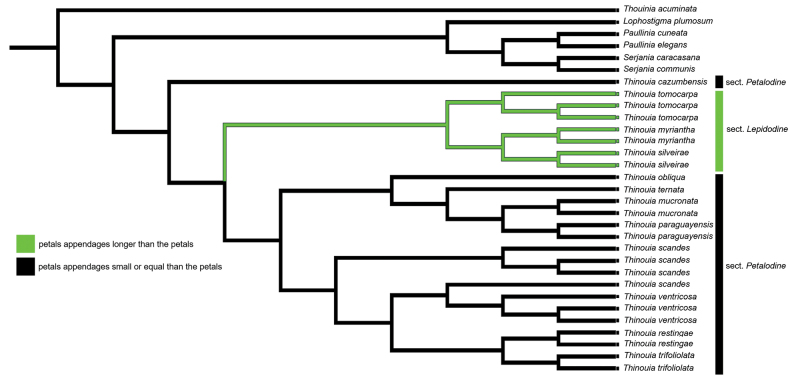
Maximum parsimony ancestral state reconstruction using Mesquite v.3.70. Ancestral states are shown as the colors of the branches (see legend).

The results of the petal appendage type reconstruction based on MP and ML were largely consistent, with the MP analysis displayed in Fig. 5. They revealed that petal appendages shorter or equal to the petals represent the ancestral state of *Thinouia*. The size of appendages relative to the petals, the trait that differentiates T.sect.Lepidodine from sect. Petalodine according to Radlkofer (1878), appears to have evolved just one time.

### ﻿Taxonomic treatment

#### ﻿ Thinouia

Taxon classificationPlantaeSapindalesSapindaceae

Triana & Planchon, Ann. Sci. Nat. Bot. Ser. 4, 18: 368. 1862

61CDE426-21C3-59FF-8B24-C6E3E8226DDC

Carpidiopterix H. Karsten, Fl. Columb. 2: 45. 1863. Type: Carpidiopterixmacroptera (Cassar.) H. Karst. = Thinouiascandens (Cambess.) Trian. & Planch.
Allosanthus Radlk. in A. Engler., Pflanzenr. IV. 165. (Heft 98): 1157. 1933. Type: Allosanthustrifoliolatus Radlk. = Thinouiatrifoliolata (Radlk.) Acev.-Rodr. & Ferrucci

##### Type.

*Thinouiamyriantha* Triana & Planchon.

##### Description.

Lianas or seldom shrubs with arched branches; climbing with the aid of a pair of circinate tendrils that are proximal to the floriferous part of the inflorescence and homologous to a cincinnus. Stems terete or ribbed, lenticellate; cross-sections of branches and young stems simple, i.e., with single vascular cylinders, some species developing a compound stem where 1–5(7–8) vascular cylinders are formed within the cortex, these becoming wider as the stem grows wider. Stipules minute, axillary, deltate to lanceolate, early deciduous. Leaves alternate, trifoliolate; petioles and petiolules unwinged. Inflorescences of umbelliform or racemiform thyrses, often bearing a pair of tendrils at the base of the rachis, axillary or forming a frondobracteate synflorescence on distal portion of branches, rarely cauliflorous, peduncle and secondary peduncle, sometimes sessiles; flowers produced in cincinni; pedicels articulated. Flowers actinomorphic; calyx cup-shaped, partly connate, sepals 5, valvate, of equal size; petals 5, obovate to spatulate, with a pair of short appendages, smaller or equal than the petals or longer than the petals, sometime clawed; disc extrastaminal, annular, rarely 5-lobed; stamens 6–8, filaments equal or in two unequal series, anthers dorsifixed; pollen isopolar, obtusely triangular in polar view, subspherical in equatorial view, tricolporate, with elongated colpi nearly reaching the poles, striate; ovary 3-carpellate, the ovules solitary with axile placentation; style elongated with 3 papillose stigmatic branches. Fruit schizocarpic, stipitate, splitting into 3 mericarps, each with a distal wing, the seed locus subglobose, lenticular or rarely flattened; seed trigonous-ellipsoid, or lenticular-ellipsoid, exarillate, with a small hilum.

### ﻿Key to the species of *Thinouia*

**Table:** 

1a	Inflorescence racemiform	**2**
2a	Epicarp glabrous; seed locule cavity sparsely ferruginous pubescent, with simple, capitate and arachnoid trichomes; nectary disc annular to lobed; petal appendages 0.8–1 mm long	***T.trifoliolata* (12)**
2b	Epicarp strigose; seed locule cavity glabrous; nectary disc 5-lobed; petal appendages rudimentary, < 0.5 mm long	***T.cazumbensis* (1)**
1b	Inflorescence umbelliform	**3**
3a	Petal appendages longer than the petals	**4**
4a	Seed locule subglobose, flattened at the base, epicarp pubescent at the locule, the wing puberulous or pilose; cavity of seed locule densely villous	***T.silveirae* (9)**
4b	Seed locule slightly flattened, epicarp sparsely pubescent at the locule, the wing sparsely pubescent; cavity of seed locule glabrous or rarely sparsely pilose, with capitate trichomes	**5**
5a	Stamens with filament villous throughout; petal appendages marginal. Belize, southern Mexico, El Salvador, and Honduras	***T.tomocarpa* (11)**
5b	Stamens with filament villous on the lower half; appendages basal. Costa Rica to Amazon basin	***T.myriantha* (4)**
3b	Petal appendages shorter than or equal to the petals	**7**
7a	Fruit locule flattened	***T.compressa* (2)**
7b	Fruit locule subglobose	**8**
8a	Cavity of seed locule with arachnoid trichomes	**9**
9a	Leaflets with entire margins, or rarely with basally one-toothed margins; venation brochidodromous, without domatia on the abaxial side of secondary vein axils	***T.restingae* (7)**
9b	Leaflets with entire or serrate margins, venation semicraspedodromous, with domatia on the abaxial side of secondary vein axils	***T.ventricosa* (13)**
8b	Cavity of seed locule with capitate trichomes	**10**
10a	Petal appendages distally bifurcated; stamens 6–7	***T.obliqua* (5)**
10b	Petal appendages distally entire; stamens 8	**11**
11a	Fruits > 4.5 cm long	***T.scandens* (8)**
11b	Fruits < 4.5 cm long	**12**
12a	Leaflet secondary veins semi-craspedodromous; lateral leaflet decurrent at the base	***T.mucronata* (3)**
12b	Leaflet secondary veins craspedodromous; lateral leaflet truncate or rounded at the base	**13**
13a	Mericarps 2.5 cm wide; epicarp glabrous; terminal leaflet widely elliptic, obtrullate or ovate. SE and NE Brazil	***T.ternata* (10)**
13b	Mericarps 1.3–2.3 cm wide; epicarp glabrous or with sparse, simple trichomes on the locule and ventral margin of the wing; terminal leaflet broadly ovate. Western Brazil, Bolivia and Paraguay	***T.paraguayensis* (6)**

#### ﻿1. Thinouia cazumbensis

Taxon classificationPlantaeSapindalesSapindaceae

H. Medeiros, PhytoKeys 165: 118. 2020.

1FDD3E2B-6A59-5B42-A5EF-BBC61DDD8AAE

Fig. 6 , 13A

##### Type.

**Brazil. Acre.** • Mun. Sena Madureira, Reserva Extrativista do Cazumbá-Iracema, Núcleo Cazumbá, castanhal coletivo, 20 July 2018, *H. Medeiros et al. 3401* (holotype: RB!, isotypes: INPA!, SPF!, UFACPZ!, US!).

##### Description.

Tendrilled liana, 6–8 m long; stem puberulent, with yellowish to whitish indumentum, lenticellate; cross-section simple, cylindrical. Leaves trifoliolate; stipules ca. 2 mm long, linear, triangular to lanceolate, hirsute-tomentose; petiole 2–8.5 cm long, canaliculate; terminal petiolule 1.2–1.7 cm long, tomentose or tomentulose, lateral petiolules 0.2–0.8 cm long; leaflets glabrous on both sides, the secondary venation eucamptodromous but distally craspedodromous; secondary veins 7–8 pairs, subalternate or alternate, spacing irregular, sometimes with domatia on abaxial secondary vein axils; intersecondaries present; tertiary veins reticulate; margins entire to dentate-serrate, with 2–4 teeth reduced to inconspicuous glands, ciliate; terminal leaflet 12–13 × 6.5–7.5 cm, oblong, the apex acute, mucronate, the base truncate or rounded to obtuse; lateral leaflet 9.5–11.5 × 4.8–5.7 cm, oblong or ovate-rhomboidal, the apex acute, mucronate, the base truncate or rounded. Thyrses axillary, racemiform, 8.5–16 cm long; peduncle 1.1–2.8 cm long; rachis 7.5–16 cm long; cincinni numerous, sessile. Flowers ca. 2 mm long, pedicel ca. 0.5 mm long; sepals ca. 1 mm long, connate at the base, lobes ovate, acute, glabrous and with prominent veins on the adaxial surface, abaxial surface villous; petals ca. 1.5 mm long, obovate, obtuse, not clawed, glabrous on the central part and villous on the margins; appendages rudimentary, ca. 0.3 mm long, bifid, shorter than the petals, adnate to central portion of petal, villous; nectary disc glabrous, 5-lobed, the lobes ca. 1 mm long. Staminate flower with stamens 8, ca. 1.5 mm long, the filaments villous for more than half of their length, the anthers glabrous; pistillode ca. 1.5 mm long. Pistillate flower with staminodes ca. 1 mm long; pistil ca. 1.5 mm long, the style villous, the ovary puberulent. Fruits ovate, chartaceous, 5–5.5 × 2–2.3 cm; stipe 2–3 mm long; seed locule slightly subglobose, 1.2–1.4 × 1.1–1.4 cm; epicarp densely strigose, with simple and capitate trichomes on cocci, strigose on wings; cavity of seed locule glabrous. Seeds trigonous-ovoid, ca. 6 × 4 mm, basally attached, glabrous.

##### Distribution, habitat and phenology.

*Thinouiacazumbensis* is known from the type and from a collection from the state of Pará, Brazil, in non-flooded tropical and subtropical moist broadleaf forests. It occurs in the Reserva Extrativista do Cazumbá-Iracema where it is an infrequent liana that reaches the canopy of the open ombrophilous forest with abundant bamboo (*Guadua* spp.) (Fig. 13A). Collected in flower during July and September, and in fruit in July.

##### Notes.

**Figure 6.**
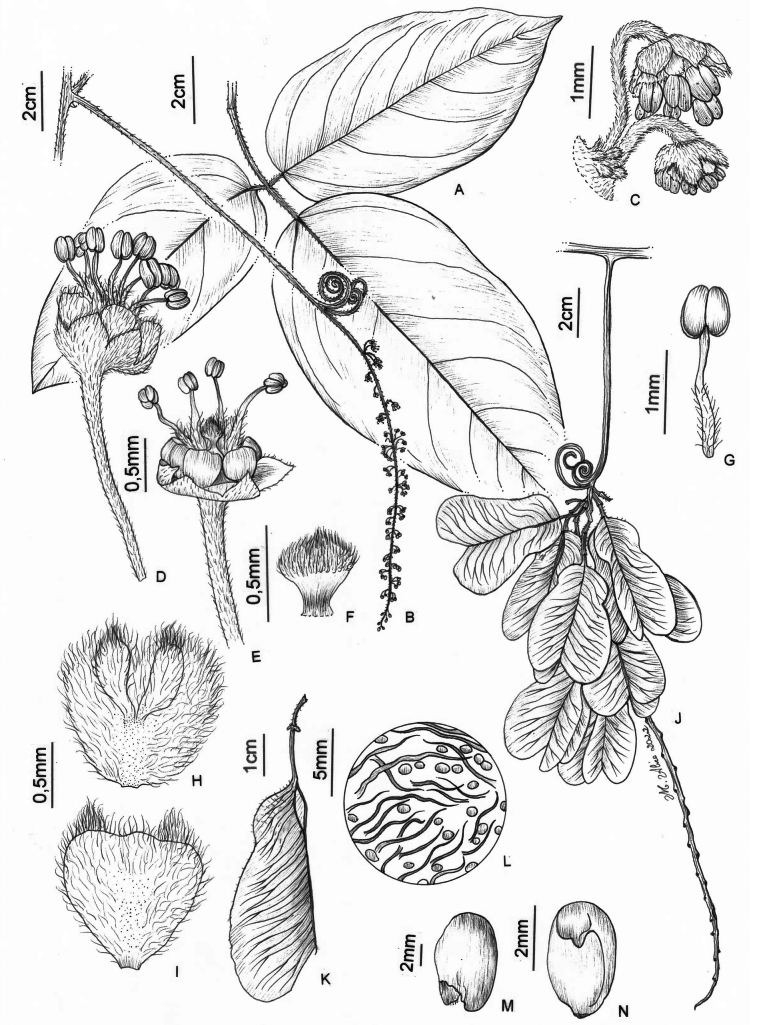
*Thinouiacazumbensis* H. Medeiros **A** leaf **B** inflorescence racemiform with tendrils **C** portion of cincinni **D** staminate flower showing sepals, petals and stamens **E** staminate flower with petals removed showing a 5-lobed nectary disc and pistillode **F** pistillode **G** stamen of staminate flower **H** petal with minute appendages, frontal [adaxial] view **I** petal in dorsal [abaxial] view **J** infructescence **K** mericarp **L** epicarp densely strigose and with glandular trichomes **M** seed **N** embryo (**A**–**N** from *Medeiros 3401*). Illustration by Maria Alice de Rezende.

*Thinouiacazumbensis* is differentiated from most species of *Thinouia* by the racemiform thyrses (Fig. 6B) and the 5-lobed nectary disc, a character recorded for the first time in the genus (Fig. 6E).

##### Conservation status.

The species is still only known from a single locality each in Acre and Pará and it is categorized as Data Deficient (DD) according to IUCN (2022). Further field studies are needed to evaluate its conservation status more accurately.

##### Additional specimen examined.

**Brazil. Pará** • Rio Jarí, Monte Dourado, terra firme forest, 17 Sep 1968, N. T. Silva 1022 (IAN, US).

#### ﻿2. Thinouia compressa

Taxon classificationPlantaeSapindalesSapindaceae

Radlk., Sitzungsber. Math.-Phys. Cl. Königl. Bayer. Akad. Wiss. München 8: 282. 1878

57E11865-1F7C-5212-BB0F-7C1B29237150

Figs 7 , 13A

Thinouia coriacea Britton, Bull. Torrey Bot., Club 16: 191. 1889. Type: Bolivia. Guanai, May 1886, *H. H. Rusby 550* (lectotype, designated here: NY! [NY00387407], isolectotypes: GH [GH10593] [image!], MICH [MICH1115486] [image!], NY! [NY00387406]).

##### Type.

**Brazil.** [**Rio de Janeiro**?] • Cantagallo, Jul 1832, *L. Riedel 513* (lectotype, designated here: US! [US00169545], isolectotypes: AAH!, LE? [not seen], NY! [NY5169]).

**Figure 7.**
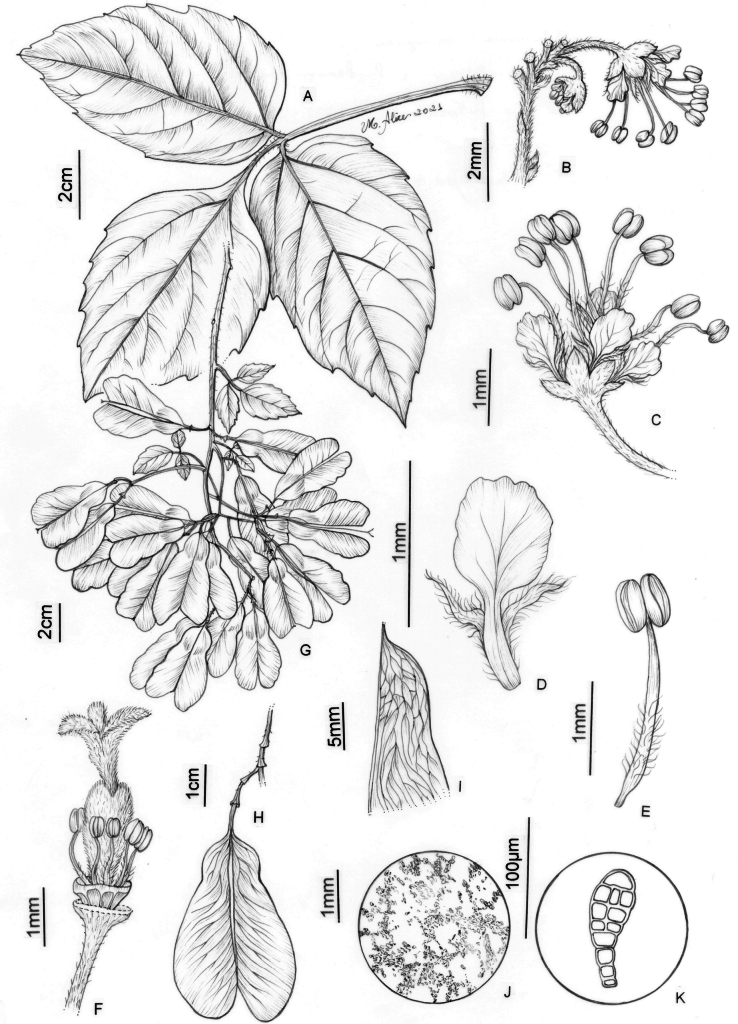
*Thinouiacompressa* Radlk. **A** leaf **B** cincinnus **C** staminate flower **D** petal with bifid appendage, dorsal [abaxial] view **E** stamen **F** pistillate flower with perianth removed showing nectary disc, staminodes and gynoecium **G** infructescence **H** fruit **I** detail of mericarp showing locule **J** detail of indumentum in seed locule cavity **K** Capitate trichome with uniseriate stalk and multicellular terminal cells (**A, G–K** from *França 4971***B–F** from *Hatschbach 67777*). Illustration by Maria Alice de Rezende.

##### Description.

Tendrilled liana; stem cylindrical, striate, puberulous, with ferruginous, rounded or elliptic lenticels; cross-section simple. Leaves trifoliolate; stipules ca. 0.8 mm long, triangular, tomentose; petiole 2–3 cm long, terete, pubescent; terminal petiolule 0.2–0.5 cm long, canaliculate, lateral petiolules 0.2–0.3 cm long; leaflets with adaxial surface glabrous or puberulous, abaxial surface puberulous or pubescent, the leaflet secondary venation semicraspedodromous or craspedodromous; secondary veins 4–5 pairs, subalternate or opposite, spacing irregular, with domatia on the abaxial side of secondary vein axils; intersecondaries present or absent; tertiary veins irregular reticulate; margins serrate or dentate-serrate, subrevolute, with (3)6–8 teeth reduced to inconspicuous glands; terminal leaflet 3.7–5.5 × 2.3–3.7 cm, ovate-rhomboid, symmetrical or asymmetrical, the apex obtuse or acuminate, mucronate, the base decurrent; lateral leaflets 3–4.8 × 2.2–3 cm, ovate, asymmetrical, the apex obtuse or acuminate, mucronate, the base subcuneate to obtuse. Thyrses axillary or terminal, umbelliform, 1.7–4 cm long; peduncle 0.7–2.4 cm long; secondary peduncle subsessile or 0.2–0.5 cm long; cincinni numerous, peduncle of cincinni 2–3.5 mm long, tomentose. Flower 2.5–4.5 mm long, pedicel 1–2 mm long, pilose or tomentose; sepals 1–1.25 mm long, connate at base, deltate, abaxially pilose, adaxially glabrous; petals 1–2.5 mm long, spatulate, distally orbiculate, clawed, the margin erose, adaxially glabrous with glands; petal appendages rudimentary, bifid, shorter than the petals, ca. 0.5 mm long, villous; nectary disc glabrous, annular. Staminate flower with stamens 8, ca. 2.5 mm long, the filaments villous on lower half, the anthers pilose to glabrous; pistillode ca. 0.6 mm long, villous at the apex. Pistillate flower with staminodes ca. 1.7 mm long, villous more than half of their length; pistil 3 mm long, villous. Fruits 3–5 × 2.2–2.3(2.7) cm; accrescent pedicel 3–5 cm long; stipe 5–8 mm long; seed locule flattened; epicarp glabrous or with sparse simple trichomes; cavity of seed locule with capitate, ferruginous trichomes with uniseriate stalk and multicellular terminal cells. Seed ellipsoid, 6–7.5 × 3.8–4.4 mm, basally attached, glabrous.

##### Distribution, habitat and phenology.

*Thinouiacompressa* is known from tropical and subtropical moist broadleaf forests, dry broadleaf forests, grasslands, savannas, shrublands, and xerophytic shrublands in Bolivia, Brazil, and Paraguay, along streams, roadside thickets, semi-deciduous forests, and caatinga vegetation in Brazil (Bahia, Ceará and Pernambuco), (Fig. 13A). Flowering from February to August, and fruiting from March to August.

##### Notes.

*Thinouiacompressa* is easily distinguished from other species of *Thinouia* by its fruits with flattened seed locules (vs. subglobose or lenticular). *Thinouiacompressa* is similar to *T.paraguayensis*. They both have leaflets with mucronate apex, margins dentate-serrate, flowers 3–5 mm long, and fruits 2.5–5 cm long, with seed ellipsoid. However, *Thinouiacompressa* differs from the *T.paraguayensis* by the adaxially glabrous or puberulous leaflets and the spatulate-orbiculate petals (vs. puberulous, pubescent-tomentose only along the veins, and spatulate-obovate petals).

##### Conservation status.

*Thinouiacompressa* occurs from Northeastern Brazil to Paraguay and Bolivia within an EOO of 3,207,299.55 km^2^ and an AOO of 140.00 km^2^. This species occurs in dry tropical forests, a habitat that is under threat as it is being converted into large-scale agricultural plantations and urban development. Despite this, the EOO values and the number of threat situations extrapolate the thresholds for inclusion of the species in a threat category. Moreover, there are no data on population declines for the application of other criteria. Thus, it should be regarded as Least Concern (LC).

##### Selected specimens examined.

**Bolivia. La Paz** • Prov. Franz Tamayo, 2 km W de la Hacienda Ubito, 850 m, 13 Jul 1993, Kessler et al. 3983 (LPB, US). **Brazil. Bahia** • Mun. Brejolândia, 5 km to the North of Tabocas, 1 Mar 1980, Harley et al. 21997 (CEPEC, SPF) • Mun. Correntina. Distrito de São Manoel do Norte, 479 m, 8 Apr 2005, Miranda et al. 706 (HUEFS) • Mun. Coribe, 545 m, 10 Apr 2007, Queiroz et al. 12715 (RB) • Mun. Espigão Mestre, 3 km S of Cocos, 14 Mar 1972, Anderson et al. 36945 (F, MO, U, UB) • Mun. Ibiquera, Gruta da Lapinha, 610–710 m, 1 Jan 2004, França et al. 4971 (RB) • Mun. Iraquara, Gruta da Lapa Doce, 19 Mar 2019, Medeiros and Sousa 3800 (RB, SPF, UFACPZ) • Mun. Oliveira dos Brejinhos, Canabrava, 16 Mar 1998, Hatschbach et al. 67777 (BHCB, HCF, MBM, RB, UB, UFACPZ) • Mun. Macaúbas, Estrada para Canatiba, 600–800 m, 20 Apr 1996, Hatschbach et al. 65076 (CEPEC, MBM) • Mun. Machado Portello, 19–23 Jun 1915, Rose et al. 19992 (US). **Ceará** • Crato, 1910, Löfgren 625 (R); Serra de Saturité, 24 Aug 1908, Ducke s. n. (INPA 12613, UB). **Espírito Santo** • Mun. Alegre, São João do Norte, 25 Jun 2008, Kollmann 11067 (CEPEC). **Mato Grosso do Sul** • Mun. Bonito, Projeto Guaicurus, 14 Mar 2003, Hatschabach et al. 74746 (HUEFS, MBM, MEXU, SPF). **Minas Gerais** • Mun. Caratinga, Fazenda Macedônia/Cenibra-Ipaba, 21 Aug 1991, Stehmann and Soares s. n.. (MBM 227949) • Mun. Januária, 13 km by road W of Januária, 19 Apr 1973, Anderson et al. 9187 (F, UB) • Mun. Leopoldina, Domingos Pisoni, 30 Mai 1936, Barreto 7558 (BHCB, F, R) • Mun. Marliéria, Parque Estadual do Rio Doce, 28 May 2001, Stehmann 2959 (ESA) • Mun. Santo Hipólito, 5 km de Santo Hipólito em direção à Monjolos, 14 Aug 1998, Rapini 628 (SPF) • Mun. São João del-Rei, Serra do Lenheiro, 17 Jun 1996, Alves 4981 (R) • Mun. Tombos, Fazenda Antilhas, 30 May 1941, Oliveira 387 (UB). **Paraíba** • Mun. Maturéia, Pico do Jabre, 1225 m, 11–13 Jul 2007, Agra et al. 6985 (JPB). **Pernambuco** • Entre Salgueiro, Cedro e Jardim, 17 May 1971, Heringer 725 (R, RB, UB). **Rio de Janeiro** • Mun. Barra, Fazenda Boa Esperança, 650 m, 8 Mai 2013, Bovini et al. 3824 (CTES, RB, UFAPZ, US) • Mun. Santa Maria Madalena, 16 km antes do portal de Santa Maria Madalena, 17 Jun 2004, Calió et al. 70 (CTES, K, NY, MBM, RB, SPF). **Paraguay. Guaira** • Cordillera de Ybytyruzú, 28 May 1989, Zardini and Velásquez 12165 (MO, US) • Road to Polilla, 800 m, 23 Jul 1989, Zardini and Velásquez 13669 (MO, US). **Paraguari** • Acahay Massif, Easternmost Peak, 13 Jan 1992, Zardini and Aquino 29700 (MO, US). **Primavera** • High woodland, 27 Jul 1956, Woolston 840 (US). **San Pedro** • Línea Caraguatay, 2 Oct 1987, Zardini and Benítez 3333 (MO, US) • Yaguarete Forest, 152 m, Zardini and Guerrero 48472 (MO, P, US) • Colonia Primavera, 17 Jul 1956, Woolston 664 (P).

#### ﻿3. Thinouia mucronata

Taxon classificationPlantaeSapindalesSapindaceae

Radlk., Sitzungsber. Math.-Phys. Cl. Königl. Bayer. Akad. Wiss. München 8: 282. 1878

FC34F210-22E8-5FDF-9F6C-E6633D1875F0

Figs 8 , 13B

Thinouia repanda Radlk. in Engler & Prantl, Nat. Pflanzenfam. 3 (5): 308. 1895. Type: Paraguay. Yaguaron, February 1877, *B. Balansa 2488* (lectotype, designated by Ferrucci 2021, pg. 401: P [P04795732] [image!], isolectotypes: K [K000634087] [image!], P [P04795732] [image!]), G [G00008258] [image!].

##### Type.

**Brazil. São Paulo** • Campinas, 1875, *H. Mosén 3953* (lectotype, designated by Massing and Miotto 2020, p. 160: S [S17-1112] [image!], isolectotypes: P [P06695515] [image!], S [S17-1101] [image!], S [S17-1104] [image!].

**Figure 8.**
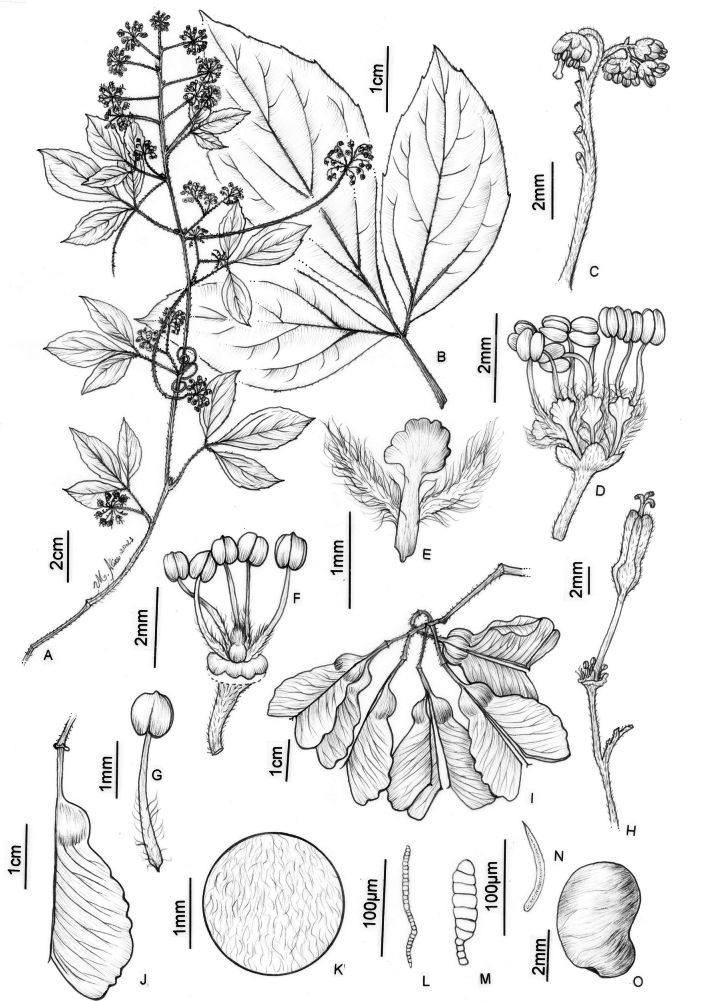
*Thinouiamucronata* Radlk. **A** distal portion of flowering branch **B** leaf showing leaflet venation **C** cincinnus **D** staminate flower **E** petal with bifid appendage, dorsal [abaxial] view **F** staminate flower with perianth removed showing nectary disc, stamens and pistillode **G** stamen **H** pistillate flower with long-stipitate, developed gynoecium **I** infructescence **J** mericarp **K** indumentum detail of seed locule cavity **L**, **M** capitate trichomes with uniseriate stalk and unicellular terminal cells **N** simple trichome from locule cavity **O** seed (**A, C–G** from *Bianek 229***B** from *Caxambu 5215***H** from *Kozera 3718***I–O** from *Caxambu 6486*). Illustration by Maria Alice de Rezende.

##### Description.

Tendrilled liana; stem puberulous or glabrescent, lenticels ferruginous, rounded or elliptic; cross-section simple or with neo formations when mature. Leaves trifoliolate; stipules minute, ca. 0.5 mm long, pubescent, triangular; petiole 1–5.5 cm long, canaliculate or sometimes semiterete, glabrous or puberulous; terminal petiolules 0–0.3 cm long, lateral petiolules 0–0.2 cm long; leaflets glabrous, puberulous along veins or sparsely pubescent on both surfaces, the leaflet secondary venation semi-craspedodromous; secondary veins 3–5 pairs, with irregular spacing and domatia on the abaxial side of secondary vein axils; intersecondaries presents; tertiary veins irregular reticulate or alternate-percurrent; margins subentire to repand-serrate, glabrous or ciliate, revolute, the teeth 2–5(8–12), sometimes reduced to inconspicuous glands; terminal leaflet 3–9.7 × 1.5–5 cm, ovate or oblong, the apex acute to obtuse, mucronate, the base decurrent; lateral leaflets 3–7.7 × 1–4.3 cm, ovate or oblong, asymmetrical, the apex acute or obtuse, mucronate, the base decurrent. Thyrses axillary or terminal, umbelliform, 2.5–7.8 long; peduncle 1.5–6.5 cm long; secondary peduncle 0–0.5 cm long; cincinni numerous; peduncle of cincinnus 2.5–4 mm long. Flower ca. 3–5 mm long; pedicel 2–2.5 mm long, glabrous or puberulous; sepals 0.5–0.8 mm long, connate at the base, deltoid, abaxially puberulous and adaxially villous; petals 1.5–1.7 mm long, spatulate, erose, clawed, abaxially glabrous and adaxially villous; appendages ca. 1 mm long, shorter than the petals, bifid, villous; nectary disc glabrous, annular. Staminate flower with stamens 8, 2.5–3.5 mm long, the filaments villous on lower half, the anthers 0.4–0.5 mm long, glabrous; pistillode <0.5 mm long, villous. Pistillate flower with staminodes ca. 1.5 mm long, with same indumentum as the stamens; pistil 0.5–1 mm long, villous. Fruits chartaceous, 2.2–3.8 × 1.5–2.6 cm; accrescent pedicel 5–6(7) mm long; stipe (4)5–8 mm long; seed locule subglobose; epicarp glabrous, sometimes puberulous on the locule, rarely tomentose on stipe and base of fruits; cavity of seed locule villous with simple and capitate trichomes with uniseriate stalk and unicellular or multicellular terminal cells. Seed 5–6 × 3–4 mm, ellipsoid.

##### Distribution and habitat.

*Thinouiamucronata* is known from tropical and subtropical moist broadleaf forests; tropical and subtropical dry broadleaf forest; tropical and subtropical grassland, savannas and shrublands; and desert and xeric shrublands in northern Argentina, southern Bolivia, SE Brazil, and Paraguay (Fig. 13B), in cerrado, chaco, gallery, ombrophilous dense, semi-deciduous and deciduous forests. Flowering from December to March and fruiting from December to August.

##### Notes.

This species is strongly supported as sister to *T.paraguayensis* (Fig. 4). They both have simple stems or with neo formations when mature, leaflets with mucronate apex, flowers 3–5 mm long, and chartaceous fruits, with seed locule subglobose. However, *T.mucronata* is distinguished by the semi-craspedodromous secondary venation and 3–5 pairs of secondary veins, where basal secondary veins form an acute angle with the midvein, (vs. craspedodromous and 4–5(6) pairs of secondaries, basal secondary veins form an obtuse angle with the midvein).

##### Conservation status.

*Thinouiamucronata* possesses a broad EOO of 3,328,158.18 km^2^ and an AOO of 320.00 km^2^, with more than 10 known localities. The EOO values and the number of threat situations extrapolate the thresholds for the inclusion of the species in a threat category. Moreover, there are no data on population declines for the application of other criteria. Thus, it should be regarded as Least Concern (LC).

##### Selected specimens examined.

**Argentina. Jujuy** • Dep. El Carmen, Abra de Santa Laura, 14 Feb 1972, Cabrera et al. 22085 (F) • Dep. General San Martín, Parque Nacional Calilegua, 18 Apr 1998, Vanni et al. 4179 (CTES, F, US) • Dep. Ledesma, Parque Nacional Calilegua, 1107 m, 30 Aug 2012, Coulleri et al. 384 (CTES, NY, RB, UFACPZ, US). **Misiones** • Dep. Apóstoles, Ayo. Chimiray y ruta 40, 9 Feb 1993, Tressens et al. 4409 (CTES, US) • Dep. Cainquás, Ruta 14, km 252, 26 Jan 1950, Schwindt 3087 (US) • Dep. Candelaria, Loreto, 2 Feb 1948, Montes 100B (US) • Dep. Guaraní, Predio Guaraní, 27 Apr 1999, Tressens et al. 6164 (CTES, MBM) • Dep. Iguazú, Acesso al Parque Nacional Iguazú, 23 Feb 2001, Vanni et al. 4518 (CTES, ESA, F, MEXU, US) • Dep. Montecarlo, Colonia Guatambú, 25 Feb 2001, Keller 622 (CTES, MEXU) • Santa Ana, 11 Jan 1913, Rodríguez 715 (F). **Salta** • Dep. La Caldera, El Ucumar, ruta 9, 12 Mar 1982, Schinini 22301 (CTES, F, ICN). **Bolivia. Chuquisaca** • Prov. Sud Cinti, Trayecto Las Abras-Cañón Verde, 903–1031 m, 6 Feb 2006, Lozano and Peñaranda 2128 (HSB, MO, US) • Prov. Tomina, Las Casas, 1280 m, 18 Apr 2005, Gutiérrez et al. 1200 (HSB, MO). **Santa Cruz** • Prov. Andrés Ibáñez, 10 km E of Cotoca, 350 m, 27 Jul 1994, Nee 45374 (NY, US) • Prov. Cordillera, 2.5 km W of railroad and 3.5 km w of the Santa Cruz-Abapó, 570 m, 24 May 2005, Nee 53120 (NY, US) • Prov. Florida, 14.2 km NE of Achira Camping, 1800 m, 17 Jan 1998, Nee 48052 (MEXU, NY, US). **Tarija** • Prov. Arce, 29.2 km S of Emboroza-Sidras road on road to Bermejo, 21 Apr 1983, Solomon 9966 (US) • Prov. O’Connor, Abra de la Cuesta de San Simón, 30 Apr 1983, Krapovickas et al. 39043 (F). **Brazil. Bahia** • Mun. Boa Nova, Fazenda Cotermaia, 8 Mar 2003, Fiaschi et al. 1395 (CEPEC, NY, SPF) • Mun. Maracás, 13–22 km ao S de Maracás, 27 Apr 1978, Mori et al. 10061 (CEPEC, MO, NY, RB) • Mun. Planaltino, ca. 6 km W de Nova Itarana, 790 m, 14 May 2001, França et al. 3507 (CEPEC, HUEFS). **Mato Grosso do Sul** • Mun. Amambai, Rio Iguatemi, 13 Feb 1983, Hatschbach 46203 (INPA, MBM, MG, MO, NY) • Mun. Antônio João, Fazenda Cervo, 11 Jun 2006, Barbosa and Silva 1448 (HUEFS, MBM, MG, RB) • Mun. Ponta Porã, Fazenda Itamarati, 9 Mar 2004, Hatschbach et al. 76943 (MBM, RB). **Minas Gerais** • Without locality, s.d., Claussen 521 (P). **Paraná** • Mun. Arapongas, Campinho, 29 Jan 1997, Kinupp 217 (FUEL, MBM) • Mun. Bom Sucesso, 460 m, Proença 82 (ICN) • Mun. Campo Mourão, RPPN Ana Tramujas, 667 m, 9 Jun 2009, Siqueira et al. 204 (HCF, MBM) • Mun. Céu Azul, Parque Nacional do Iguaçu, 674 m, 18 Jun 2015, Caxambu et al. 6559 (HCF) • Mun. Cianorte, Estrada Cambuci, 12 Feb 2013, Rosado 371 (HCF, HUEM) • Mun. Diamante do Norte, Estação Ecológica do Caiuá, 24 Feb 2006, Zeiden 34 (HUEM, RB) • Mun. Doutor Camargo, Rio Ivai, 16 May 1969, Hatschbach et al. 21527 (MBM) • Mun. Entre Rios do Oeste, Linha Divisa, 229 m, 29 Jan 2015, Siqueira et al. 1385 (MBM) • Mun. Fênix, RPPN Vila Rica, 16 May 2014, Caxambu et al. 5215 (MBM) • Mun. Figueira, Fazenda São Pedro, 26 Apr 2001, Pavão s. n.. (FUEL, RB422905) • Mun. Foz do Iguaçu, Parque Nacional do Iguaçu, 203 m, 4 Jun 2015, Caxambu et al. 6486 (HCF) • Mun. Goioerê, RPPN Moreira Sales, 27 Mar 2017, Siqueira et al 2162 (HCF) • Mun. Guaira, Parque Nacional de Sete Quedas, 21 Mar 1982, Kirizawa et al. 756 (SP) • Mun. Guarapuava, Estrada para Campo Mourão km 7, 6 Feb 1969, Hatschbach 21007 (MBM, NY) • Mun. Ibiporã, Fazenda Doralice, 24 Apr 2003, Urdampilleta 148 (FUEL, HCF) • Mun. Jaboti, Água Branca, 18 Mar 1994, Hatschbach et al. 60563 (MBM, MEXU, MO) • Mun. Londrina, Fazenda Ramses-Distrito de São Luiz, 12 Mar 2003, Urdampilleta et al. 118 (FUEL, HCF) • Mun. Mamboré, Fazenda São Domingos, 16 May 1967, Lindeman and Haas 5325 (MBM, RB) • Mun. Maringá, 517 m, 16 Jan 2013, Proença 89 (ICN) • Mun. Medianeira, Estrada para Santa Helena km 10, 8 Feb 1969, Hatschbach 21077 (MBM, SP) • Mun. Moreira Sales, Fazenda Moreira, 24 Mar 2007, Marques s. n.. (HCF 5351, MBM) • Mun. Palotina, Parque Estadual de São Camilo, 20 Jan 2011, Kozera and Cardozo 3770 (MBM) • Mun. Pitanga, 19 Feb 2005, Bianek 229 (HCF, MBM) • Mun. Planalto, Estrada de terra próximo ao Rio Capanema, s.d., Rodriguea et al. 133 (ESA) • Mun. Porto Rico, Mata ciliar do Rio Paraná, 15 Jan 1987, Soares-Silva et al. 26 (FUEL, RBR) • Mun. Primeiro de Maio, Mata Santa Rosa-Distrito de Ibiaci, 11 May 1998, Francisco s. n.. (FUEL, MBM 338060, RBR) • Mun. Rolândia, Córrego dos Carangueijos, 24 Jan 1997, Kinnup s. n.. (FUEL27790, R) • Mun. São Jorge do Oeste, Rio Iguaçu-Salto Osório, 10 Jun 1968, Hatschbach and Guimarães 19360 (MBM, NY) • Mun. Sertaneja, Fazenda Tangará, 21 May 1999, Francisco s. n.. (FUEL, MBM338061) • Mun. Telêmaco Borba, Dec 2011, Bonaldi 506 (MBM) • Mun. Tuneiras do Oeste, Fazenda Água do Índio, 22 Jan 2004, Bianek 172 (HCF) • Mun. Turvo, Propriedade da família Rickli, 1104 m, 27 Feb 2009, Caxambu et al. 2524 (HCF, MBM) • Mun. Ubiratã, Sítio Invicta, 409 m, 9 Jan 2009, Sekine et al. 97 (HCF, MBM) • Mun. Umuarama, Serra Dourada, 19 Jan 1967, Hatschbach 15753 (MBM). **Rio de Janeiro** • Mun. Cabo Frio, Estação Radiogoniométrica de Campos Novos, 12 Jun 2009, Somner et al. 1354 (RBR) • Mun. Miguel Pereira, Sítio Xapuri, 12 Jan 2006, Menescal 103 (RB, UFAPZ) • Mun. Paraty, Estrada do Cabral, 19 Apr 1994, Marquete 1692 (RB, UFACPZ) • Mun. São Pedro da Aldeia, Ilha dos Macacos, 14 Apr 2019, Bastos and Neves F5 (RB). **Rio Grande do Sul** • Mun. Arroio do Tigre, Barragem de Itaúba, 19 Apr 1978, Lise 5860 (F) • Mun. Caxias do Sul, Caravágio, 27 Jan 1999, Kegler et al. 153 (US) • Mun. Giruá, Granja Sodol, 20 Dec 1966, Hagelund 4947 (ICN) • Mun. Ibarama, 11 Feb 2013, Proença 123 (ICN) • Mun. Iraí, Ladera con selva frente al Balneario Oswaldo Cruz, 29 Jan 1992, Krapovickas and Cristóbal 44016 (CTES, MBM) • Mun. Jaboticaba, IFN-Conglomerado 752-2-10-37, 24 May 2013, Maihack s. n.. (RB613850) • Mun. Jaguari, Rio Jaguari, 5 Jan 2011, Durigon and Ferreira 456 (ICN) • Mun. Linha Nova, Roseiral, 1 Feb 2018, Massing 125 (ICN) • Mun. Maquiné, 6 Jun 2013, Proença 115 (ICN) • Mun. Pontão, Projeto de Assentamento Encruzilhada Natalino III e IV, 30 Aug 2008, Grings 346 (ICN) • Mun. Nova Petrópolis, 3 Apr 2018, Massing 224 (ICN) • Mun. Santa Maria, BR-158, 20 Feb 2019, Figueira et al. 927 (RB, SMDB) • Mun. Santa Rosa, 8 Jul 1965, Hagelund 3678 (ICN) • Mun. São Jeronimo, Faz. Do Conde, 17 May 1982, Abruzzi 655 (F) • Mun. Tenente Portela, Parque Estadual do Turvo, Mar 1982, Bueno et al. s. n.. (ICN 2808) • Mun. Três de Maio, Feb 1967, Hagelund 5243 (ICN) • Mun. Tupanciretã, Projeto de Assentamento Tarumã, 7 Feb 2008, Grings 1272 (ICN) • Mun. Vale do Sol, Linha XV de Novembro, 27 Feb 1993, Jarenkow 2343 (MBM, PEL). **Santa Catarina** • Mun. Aberlado Luz, Comunidade da Grama, 110 m, 14 Apr 2009, Stival-Santos 588 (HCF) • Mun. Belmonte, 17 Mar 2011, Durigon 607 (ICN) • Mun. Florianópolis, Cachoeira do Bom Jesus, 6 May 1970, Klein 8690 (MBM, PEL) • Mun. Itapiranga, Rio Piperiguaçú, 26 Dec 1973, Karhs and Schenkel s. n.. (ICN2810) • Mun. Seara, Nova Teutônia, 11 Jan 1944, Glaumaum 289 (RB). **São Paulo** • Mun. Campinas, 30 Jan 1995, Novaes 3202 (SP) • Mun. Coronel Macedo, Bairro dos Costas, 24 Jan 1996, Souza et al. 10426 (BHCB, ESA) • Mun. Guariba, Acesso da estrada SP 255 para Guariba, 11 Mar 1991, Cordeiro et al. 834 (SP) • Mun. Piracicaba, Mata da Pedreira ESALQ/USP, 20 Apr 1985, Catharino 288 (ESA, MBM, SP) • Mun. Monte Alegre, Amparo, 7 Apr 1943, Kuhlmann 610 (S) • Mun. Socorro, Entre Saltinho - Monte Sião, 9 May 1995, Tamashiro et al. 1015 (ESA, SP, SPF) • Mun. Teodoro Sampaio, Parque Estadual do Morro do Diabo, 23 Jun 1994, Pastore et al. 514 (SP, SPF). **Paraguay. Canendiyú** • Mbaracayu Natural Reserve, 10 Jun 1998, Zardini and Chaparro 48588 (MEXU, MO). **Concepción** • Estancia Primavera, 28 Jun 2002, Zardini and Guerrero 59086 (FACEN, MO, US). **Guairá** • Colonia Independencia. Camino a San Gervacio, 25 Mar 1993, Schinini et al. 27933 (CTES, MBM, US). **Itapúa** • Ruta 1, 65 km de Encarnación, 17 Aug 1980, Ferrucci 161 (CTES, F, NY, US). **Misiones** • San Ignacio. Rutal, 5 km de San Ignacio, 30 Mar 1981, Ferrucci and Schinini 179 (CTES, F, MBM). **Paraguari** • Parque Nacional Ybycui, 13 Jan 1983, Hahn et al. 1072 (MO, NY); Acahay Massif. Easternmost Peak, Zardini and Tilleria 29772 (MO, PY, US). **San Pedro** • Yaguareté forest, 20 Jun 1995, Zardini et al. 42893 (MO, PY, US) • San Estanislao. Estancia La Manina, 13 Feb 1975, Pedersen 11042 (MBM).

#### ﻿4. Thinouia myriantha

Taxon classificationPlantaeSapindalesSapindaceae

Triana & Planch., Ann. Sci. Bot. Ser. 4, 18: 369. 1862

3285D259-A650-5666-9698-83318C128CAD

Figs 9 , 10 , 13C

Carpidiopterix macroptera sensu H. Karst., Fl. Columb. 2: 45. 1863, not Thouiniamacroptera Casar.

##### Type.

**Colombia. Prov. de Bogotá** • Tocaima, Limba, alt. 450 m, May 1857, *J. Triana s. n..* (lectotype, designated here: MPU [2 sheets] [MPU010893, MPU010892] [image!]).

**Figure 9.**
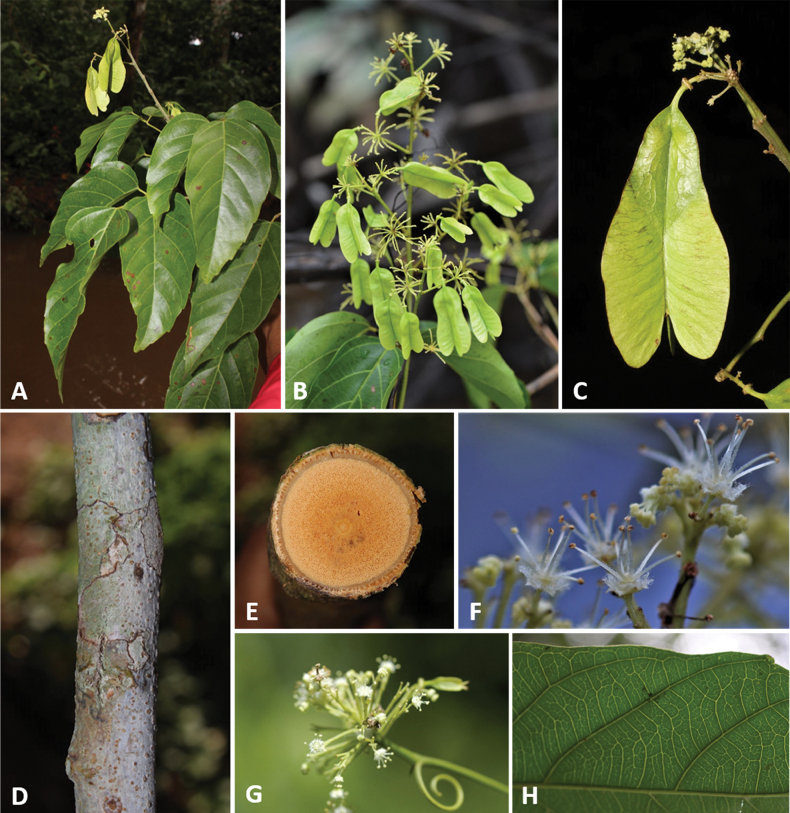
*Thinouiamyriantha* Triana & Planch. **A** fertile branch **B** fruiting branch **C** immature fruits **D** cylindrical stem **E** cross-section of the simple stem **F** staminate flower **G** detail of inflorescence **H** detail of the margin and secondary veins [Acevedo-Rodríguez 17136 (**B**) 17128 (**F**) 14262 (**G**); Medeiros 3330 (**A, C–E** and **H**); photos: **A, C, D, E, H** by H. Medeiros **B, F–G** by P. Acevedo-Rodríguez].

**Figure 10.**
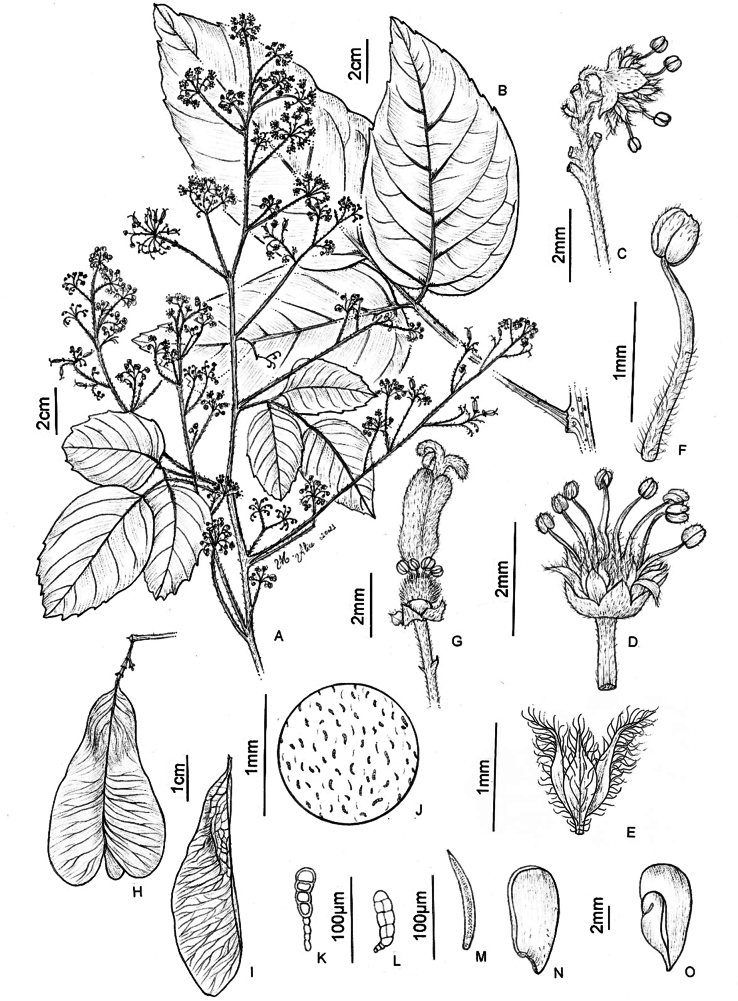
*Thinouiamyriantha* Triana & Planch. **A** flowering branch **B** leaf **C** portion of inflorescence, displaying a cincinnus and staminate flower **D** staminate flower **E** petal with bifid appendage, dorsal [abaxial] view **F** stamen of staminate flower **G** young fruit showing nectary disc **H** fruit **I** Mericarp **J** indumentum detail of locule cavity **K** capitate trichome with uniseriate stalk and unicellular terminal cells from locule cavity **L** capitate trichome with uniseriate stalk and multicellular terminal cells from locule cavity **M** simple trichome from locule cavity **N** seed **O** embryo (**A–F** from *Costa 359***H–O** from *Medeiros 3330*). Illustration by Maria Alice de Rezende.

##### Description.

Tendrilled liana 10–15 m; stem cylindrical, 10–12 cm diam., puberulous and ferruginous, lenticels rounded or elliptic and ferruginous; cross-section simple. Leaves trifoliolate; stipules minute, ca. 0.4 mm long, tomentose; petiole 1.8–6 cm long, terete, striate, pubescent, puberulous to glabrescent; terminal petiolule 1–4 cm long, lateral petiolules 0.3–1.5 cm long; leaflets glabrous on both sides, sometimes with sparse trichomes along veins; the leaflet secondary venation eucamptodromous or mixed semicraspedodromous at the apex; secondary veins (4)5–6 pairs, subalternate or alternate, spacing irregular, with domatia on abaxial side of axils; intersecondaries present; tertiary veins irregular reticulate to mixed alternate-opposite percurrent; margins entire to repand-serrate (rarely serrate), with (2)4–8 teeth per side, reduced to inconspicuous glands; terminal leaflet 6.5–17.5(22) × 3.5–10.5(11.3) cm, elliptic-ovate, apex acute or obtuse, long-apiculate, base truncate, obtuse or rounded; lateral leaflets 5.5–15.5(17) × 2–8.9(9.5) cm, ovate, asymmetrical, apex obtuse or acute and acuminate without drip tip, base truncate or rounded, sometimes cordate. Thyrses axillary or terminal, umbelliform, 1.5–10 cm long, peduncle 1–8.6 cm long, secondary peduncle 0.1–1.8 cm long; cincinni numerous, peduncle of cincinni 0.4–0.5 cm long, tomentose. Flower 1–2.5 mm long, pedicel 0.8–3 mm long, pubescent or tomentose; sepals 0.5–1 mm long, oblong-ovate, abaxially villous or slightly villous and adaxially glabrous; petals 0.4–0.8 mm long, lanceolate to obdeltoid, not clawed, villous; petal appendages 0.7–1.5 mm long, longer than the petals, bifid, villous; nectary disc annular, glabrous. Staminate flowers with stamens 8, 2–3 mm long, filaments villous on lower half, anthers glabrous to sparsely villous; pistillode ca. 0.5 mm long, villous. Pistillate flower with staminodes 8, ca. 1.5 mm long, with same indumentum as stamens; pistil ca. 4.5 mm long, ovary tomentose, stigma and styles tomentose. Fruits chartaceous, 2–5.4 × 1–3.2 cm; accrescent pedicel 1.6–3.5 mm long; stipe 2–5.1 mm long; seed locule slightly flattened; epicarp sparsely pubescent; cavity of seed locule with simple unicellular, multicellular and ferruginous trichomes. Seed 6–7.2 × 3.2 mm, ellipsoid, flat.

##### Distribution and habitat.

*Thinouiamyriantha* is a species widely distributed from Costa Rica to South America, skirting the Amazon basin (i.e., peri-Amazonian distribution sensu de Granville 1992), known from tropical and subtropical moist broadleaf forests in Costa Rica, Panama, Colombia, Venezuela, the Guianas, Ecuador, Peru, Bolivia, and Brazil (Fig. 13C). Flowering from November to February, and fruiting from December to August.

##### Notes.

According to our phylogenetic analyses, *Thinouiamyriantha* is sister to *T.silveirae* (see comments in *T.silveirae*), with both species forming a clade that is sister to *T.tomocarpa*. These species together form a clade characterized by the (1) petal appendages that are longer than the petal; (2) fruit cocci that are slightly flattened; (3) cross-section of stem simple; and (4) size and shape of leaflets. The species in this clade, however, are differentiated by the distribution of hairs on the stamens. *Thinouiamyriantha* is distinguished by the filaments that are villous only on lower half, white in *T.tomocarpa*, and the stamens that are villous from the base to the connective (see comments in *T.tomocarpa*). *Thinouiamyriantha*, as currently recognized, has an ample distribution with substantial morphological variation, and possibly includes more than one species. The resolution of this issue requires the analysis of additional data, including more molecular, morphometry and anatomical data to fully understand if there is some congruence between phylogenetic results and morphological variation.

In the current study, it was found that only a single collection has been made in the type locality region and that only the specimen housed in MPU was located, so that the MPU specimen is designated here as the lectotype.

##### Conservation status.

*Thinouiamyriantha* possesses a broad EOO of 5,040,364.60 km^2^ and AOO of 168.00 km^2^, with more than 10 threat situations and records in conservation units. The EOO values and the number of threat situations extrapolate the threshold for the inclusion of the species in a threat category. Furthermore, several conservation units protect the species. Thus, *T.myriantha* was considered as Least Concern (LC).

##### Selected specimens examined.

**Bolivia. La Paz** • Prov. Abel Iturralde, Parque Nacional Madidi, 535 m, 19 May 2001, Macía et al. 4542 (US) • Prov. Sud Yungas, Sapecho ca. 5m E del Río Beni, 28 Mar 1986, Beck 13309 (US). **Brazil. Acre** • Mun. Bujari, Rio Antimary, 12 Dec 2016, Frazão et al. 417 (SPF, UFACPZ) • Mun. Marechal Thaumaturgo, Rio Bagé, 5 Dec 2000, Daly et al. 10444 (NY, UFACPZ, US) • Mun. Rio Branco, Área de Proteção Ambiental Lago do Amapá, 20 Nov 2017, Medeiros et al. 3788 (RB, SPF, UFACPZ) • Mun. Sena Madureira, Reserva Extrativista do Cazumbá-Iracema, 4 Dec 2019, Medeiros et al. 4300 (NY, RB, SPF, UFACPZ, US) • Mun. Mâncio Lima, Parque Nacional da Serra do Divisor, 8 Dec 2022, Medeiros et al. 4898 (NY, RB, SPF, UFACPZ, UPCB). **Amazonas** • Mun. São Paulo de Olivença, Camatian, 23 Jan 1949, Fróes 23958 (IAN, UB). **Pará** • Mun. Belterra, Flona do Tapajós, 7 Feb 2017, Torke et al. 2024 (HSTM, RB, UFAPZ) • Mun. Parauapebas, Serra dos Carajás, 10 Jan 1995, Rodrigues 1632 (IAN) • Mun. Santarém, Floresta Nacional do Tapajós, 29 Apr 2010, Nascimento 33 (IAN) • Mun. Vitória do Xingu, 3°18'S, 51°47'W, 7 Jan 2015, Gonçalves PSACF_EX04702 (RB, UFACPZ). **Rondônia** • Mun. Porto Velho, Parque Nacional do Mapinguari, 13 Dec 2013, Silveira et al. 500 (INPA, RB, RON). **Colombia. Atlántico** • Cerca a Usiacurí, 2 Jan 1949, Molina et al. 19At075 (COL, US) • Barranquilla and vicinity, Dec 1934, Elias 1266 (F-2, MEDEL, US). **Bolivar** • Loma de los Colorados near San Juan de Nepomuceno, 31 Dec 1992, Gentry et al. 78457 (U, US) • Cartagena. Cerro de la Popa, 4 Feb 1962, Saraiva and Johnson 42 (COL). **Magdalena** • Delta of Magdalena river, 28 May 1935, Dugand 872 (F) • Roadside 10 km north of Codazzi, 23 Nov 1943, Haught 3867 (COL, RB, US) • Along stream near La Paz, 12 Jan 1944, Haught 3962 (COL, MO, US) • Barro Blanco, 29 Nov 1945, Haught 4746 (COL, F, MEDEL, NY, US) • Santa Marta. Agua Dulce Road, 2 Jan 1898, Smith 882 (F, MO, NY, U, US). **Meta** • Sierra de La Macarena, Río Guapaya, 21 Jan 1950, Philipson et al. 2197 (COL, MEDEL, US). **Costa Rica. Puntarenas** • Parque Nacional Corcovado Sirena Trail, 6 Jan 1989, Kernan 867 (F, MEXU, MO, US). **San José** • Carara National Park, 3 Apr 1993, Gentry et al. 79370 (MO). **Ecuador. Napo** • Tiputini Biodiversity Station, 18 Mar 1998, Burnham et al. 1671 (F, QCNE, US) • La Joya de los Sachas. Comunidad Indillama, 250 m, 14–28 Jan 1994, Grijalva et al. 450 (QCNE, MO, US) • Orellana. Via a los Pozos Gacela, 250 m, 8 Aug 1993, Palacios 11034 (QCNE, US). **French Guiana** • Haut Camopi Bauin Oyapock, 1 Feb 1949, De La Rue s. n.. P0669512 (P) • Aratai River, 1–2 km downstream, 22 Feb 2003, Acevedo-Rodríguez et al. 12359 (NY, US). **Guatemala** • Izabal bet Virginia and Lago Izabal, 50–100 m, 4 Apr 1940, Steyermark 38774 (F). **Guyana. [Without Region**] • Kanuku Mts., Rapununi R., Crabwood Cr., 100 m, 3 Jul 1995, Jansen-Jacobs et al. 4314 (F, MO, P, U, US). **U. Takutu-U. Essequibo** • Trewa River 0–5 km N of confluence of Rewa and Kwitaro Rivers, 90 m, 26 Feb 1997, Clarke 3964 (U, US). **Panama. Panamá** • Barro Colorado Is., Zetek, 26 Feb 1971, Foster 2204 (F) • Serrania de Majé, 300 m, 28 Jan 1984, Churchill and Nevers 4441 (MO). **Peru. Cuzco** • Prov. Cuzco, Campamento San Martín-C, 467 m, 15 Jan 1997, Acevedo-Rodríguez et al. 8788 (USM) • Prov. La Convención, Distrito Echarate, Chahuares, 805 m, Huamantupa and Carrión 9075 (MO). **Loreto** • Prov. Manu, Puerto Maldonado, Los Amigos Biological Station, 24 Nov 2003, Maceda et al. 1097 (US) • Prov. Maynas, Iquitos, Estación Experimental del Instituto de Investigaciones de la Amazonía Peruana, 24 Aug 1990, Vásquez et al. 14290 (MO, US) • Prov. Requena, Jenaro Herrera, 26 Feb 2010, Zárate 14005 (USM). **Madre de Dios** • Cocha Cashu Station, 350 m, 26 Nov 1980, Foster 5905 (F, NY) • Tambopata Tourist Camp, 260 m, Gentry and Ortiz 78338 (MO) • Trail from CICRA to Cocha Lobos, 9 Aug 2003, Acevedo-Rodríguez et al. 14262 (NY, US). **San Martín** • Fundo Pampahermosa (Huicte), 10 Jun 1964, Schunke-Vigo 6533 (F, USM). **Ucayali** • Upper Ucayali, Mashea, s.d., Tessmann 3314 (NY). **Suriname. Brokopondo** • Brownsberg, Mazaronitop, 23 Nov 2003, van Andel et al. 4485 (U, US). **Sipaliwini** • Sipaliwini Region, Voltzberg Nature Reserve, 100 m, Hoffman et al. 5308 (US) • Wilhelmina-Gebergte, 5 Jun 1926, B. W. 6988 (U) • In montibus Bakhuis inter flum. Kabalebo et Coppename Sinistruim, 30 Dec 1964, Florschütz and Maas 2577 (U). **Venezuela. Delta Amacuro** • Río Grande 37 km, este Noreste de El Palmar, 320 m, 10 Feb 1964, Steyermark 93135 (F, NY, P, U, US, VEN) • El Palmar-Raudal trail 2–6 km SW of Río Ganame, 22 Nov 1955, Wurdack and Monachino 38715 (US). **Zulia** • Alrededores de la Represa Burro Negro, 12 Feb 1980, Bunting 8744 (NY). **Yaracuy** • Los Cañizos, plains of the Yaracuy river, 50 m, Jan 1920, Pittier 8758 (NY, US).

#### ﻿5. Thinouia obliqua

Taxon classificationPlantaeSapindalesSapindaceae

Radlk., Sitzungsber. Math.-Phys. Cl. Königl. Bayer. Akad. Wiss. München. 8(3): 282. 1878

589841D3-2524-5B74-9A4F-FF86ADE8FA75

Figs 11 , 12 , 13D

##### Type.

**Peru** • 1875, *Ruiz and Pavón 916* (lectotype, designated here: MA [MA813132] [image!], isolectotypes: B†? [F-Negative239294] [image!], F [V0438017F] [image!], F [V031742F] [image!], FI [FI004651] [image!], G [G00008259] [image!], MA [MA813131] [image!], MA [MA813133] [image!], MA [MA817669] [image!], MA [MA817670] [image!]).

**Figure 11.**
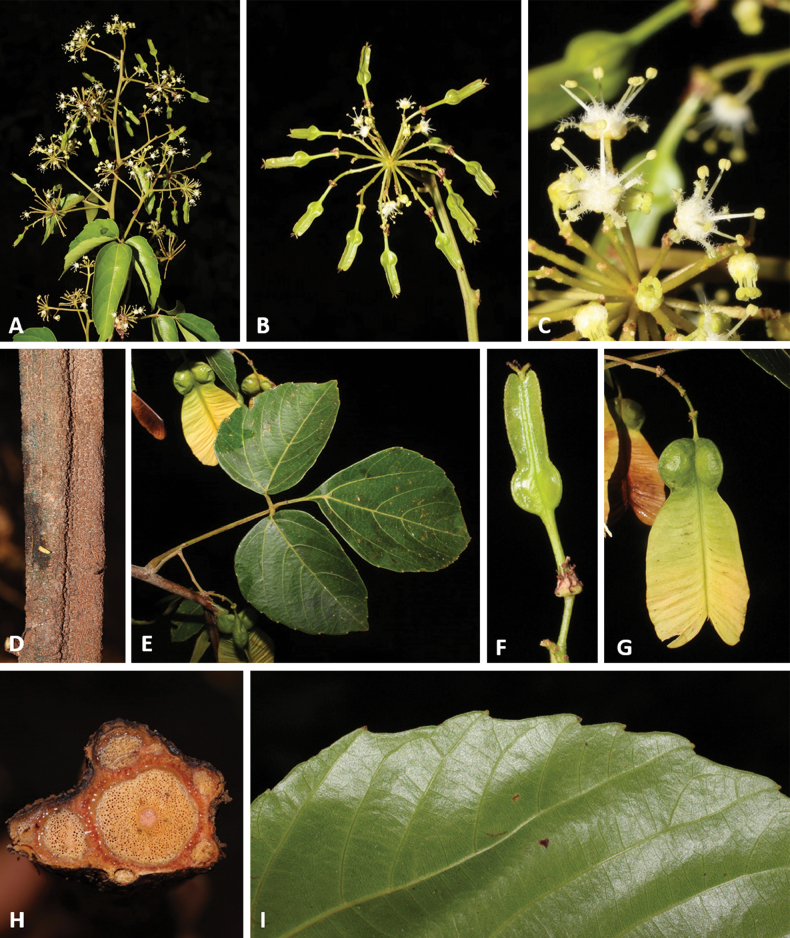
*Thinouiaobliqua* Radlk. **A** fertile branch **B** inflorescence branch with immature fruits **C** staminate flower **D** sulcate stem **E** leaf **F** immature fruit **G** mature fruit **H** stem with neo formations, central vascular cylinder surrounded by 7 neoformed, peripheral vascular cylinders of various sizes **I** leaflet margin [Medeiros 3332 (**A–D, I**) 3832 (**E–H**); photos: by H. Medeiros].

**Figure 12.**
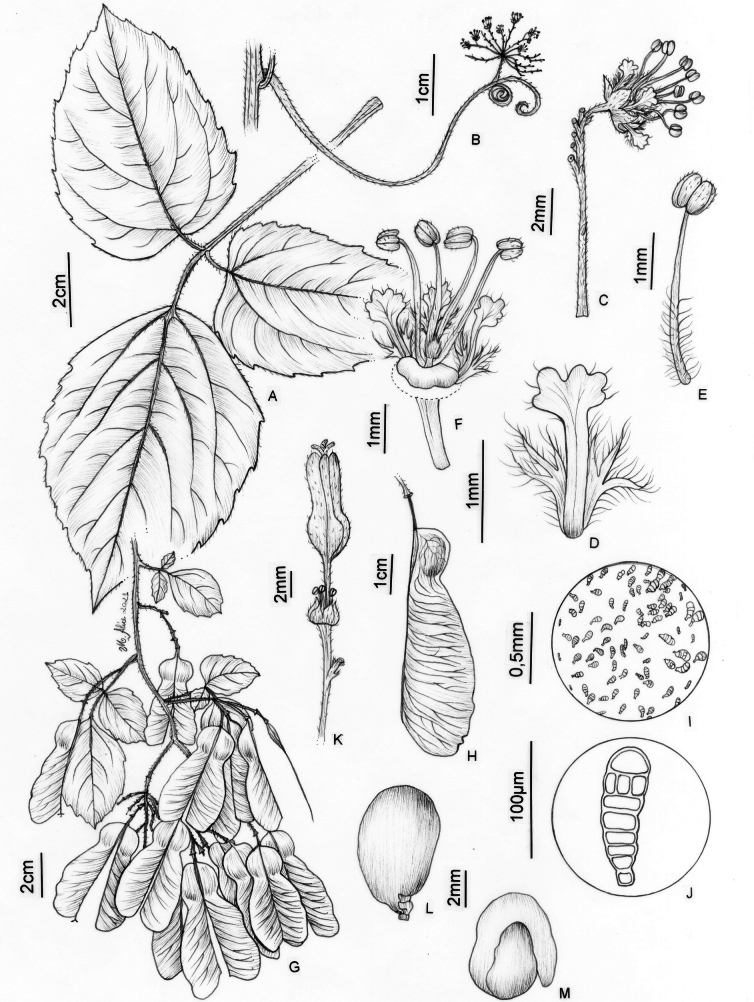
*Thinouiaobliqua* Radlk. **A** leaf **B** inflorescence with coiled tendrils **C** cincinnus with a staminate flower **D** petal with bifid and distally branched appendage, dorsal [abaxial] view **E** stamen of staminate flower **F** staminate flower with portion of perianth removed showing nectary disc, pistillode, petals with appendages and stamens **G** infructescence **H** mericarp **I** indumentum detail of locule cavity **J** capitate trichome with uniseriate stalk and multicellular terminal cells in locule cavity **K** pistillate flower with young fruit **L** seed **M** embryo (**A, G–M** from *Medeiros 3832***B–F** from *Medeiros 3332*). Illustration by Maria Alice de Rezende.

**Figure 13.**
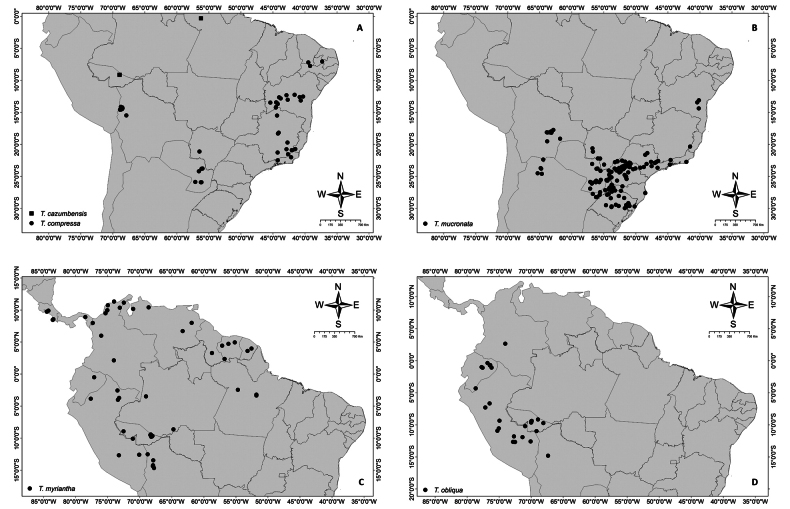
Distribution maps of species from *Thinouia***A***Thinouiacazumbensis* and *T.compressa***B***T.mucronata***C***T.myriantha***D***T.obliqua*.

##### Description.

Tendrilled liana, up to 40 m long (fide *Mexia 6675*); stem cylindrical-striate or 4–5 sulcate, 10–12 cm diam., pubescent, densely lenticellate, rounded and ferruginous; cross-section simple when young or with neo formations when mature. Leaves trifoliolate; stipules minute, ca. 1 mm long, tomentose; petioles 1–6.5 cm long, terete, striate, pubescent; terminal petiolule 0.1–1.1 cm long, lateral petiolules 0.1–1.1 cm long; leaflets with the adaxial side glabrescent or puberulous, puberulous or pubescent in the veins, the abaxial side glabrescent, puberulous or pubescent, puberulous or pubescent on the veins; the leaflet secondary venation craspedodromous; secondary veins (4)5–6 pairs, subalternate or alternate, spacing irregular, domatia present in abaxial surface of secondary vein axils; intersecondaries present; tertiary veins alternate percurrent; margins dentate-serrate, with (6)7–12 teeth on one side, reduced to inconspicuous glands; terminal leaflet 4.9–13.6 × 2.9–5.8 cm, elliptic-ovate or ovate-rhomboidal, symmetrical or asymmetrical, the apex acute to acuminate, mucronate, the base rounded to obtuse, sometimes slightly decurrent; lateral leaflets 3.6–11.1 × 1.8–7 cm, ovate, asymmetrical, the apex acute to acuminate, mucronate, the base truncate to rounded. Thyrses axillary or terminal, umbelliform, 2–5 cm long; peduncle 1–3 cm long; secondary peduncle subsessile or 0.1–0.6 cm long; cincinni numerous, peduncle of cincinnus 0.4–0.8 cm long, pubescent. Flower 4–5.5 mm long, pedicel 1.5–2.5 mm long, pilose or pubescent; sepals 0.5–1 mm long, deltoid, villous on both surfaces; petals 1.7–2.2 mm long, spatulate, clawed, glabrous or villous; petal appendages 0.7–1 mm long, shorter than the petals, bifid, distally bifurcated, villous; nectary disc annular, glabrous. Staminate flowers with stamens 6–7, 2–3.5 mm long, the filaments villous on lower half, the anthers papillose, glabrous or pilose; pistillode 0.5–1 mm long, villous. Pistillate flower with staminodes 6–7, 1.5–2 mm long; pistil 3.5–4.5 mm long, the ovary glabrous, the stigma and styles villous. Fruits chartaceous, 3.5–5.5 × 2.3–2.7 cm; accrescent pedicel 3.4–5 mm long; stipe ca. 2 mm long; seed locule subglobose; epicarp glabrous or with sparse trichomes; cavity of seed locule with simple or capitate, ferruginous trichomes; capitate trichomes with uniseriate stalk and multicellular terminal cells. Seed 6–6.7 × 5.5 mm, ellipsoid or subglobose, glabrous.

##### Distribution and habitat.

*Thinouiaobliqua* is known from Tropical and Subtropical Moist Broadleaf Forests, between 140–1000 m in the Amazon basin in western Brazil, Bolivia, Colombia, Ecuador and Peru (Fig. 13D). Flowering from October to May, and fruiting from January to August.

##### Notes.

Although our phylogenetic reconstruction suggests that *T.obliqua* and *T.myriantha* belong to different clades, these two species are vegetatively similar and are partly sympatric. Nevertheless, *T.obliqua* is differentiated by the long-spatulate petals, 1.7–2.2 mm long; petal appendages that are shorter than the petals, 0.7–1 mm long, bifid and distally branched; and stamens 6–7 (vs. small obdeltoid petals, ca. 0.7 cm long; petal appendages longer than the petals, 1–1.5 mm long, bifid and not branched distally; stamens 8). In addition, stems in *T.obliqua* present neo formations, simple in *T.myriantha*.

In the protologue for *T.obliqua*, Radlkofer (1878) mentioned that the material used in the description was collected by *Ruiz and Pavón 916* in Peru. Of the ten duplicates of this collection known to us, we are selecting a duplicate at MA [MA813132] as lectotype because it is more representative.

##### Popular name.

Macote, Macote Negro (Peru), Pacaguasca (Ecuador).

##### Conservation status.

*Thinouiaobliqua* occurs in the Western Amazon and possesses a broad EOO of 1,000,444.55 km^2^ and an AOO of 104.00 km^2^. It is protected by several conservations units, such as the Reserva Extrativista do Cazumbá-Iracema in the state of Acre (Brazil), Parque Nacional Natural Tinigua in the Department of Meta (Colombia), and Los Amigos Biological Station in the Department of Madre de Dios (Peru). Thus, *T.obliqua* should be regarded as Least Concern (LC).

##### Selected specimens examined.

**Bolivia. Beni** • Prov. Ballivián, Río Beni above confluence with Río Quiquibey, 320 m, 22 May 1990, Daly et al. 6570 (US). **La Paz** • Prov. Franz Tamayo, Serranía de Chepite, 700 m, Killeen et al. 3825 (LPB, F, MO, US). **Brazil. Acre** • Mun. Brasiléia, Rio Acre, 22 Mar 1998, Daly et al 9727 (MO, NY, UFACPZ) • Mun. Bujari, Riozinho do Andirá, 21 Jan 2018, Medeiros et al. 3328 (RB, SPF, UFACPZ) • Mun. Mâncio Lima, Parque Nacional da Serra do Divisor, 6 Dec 2022, Medeiros et al. 4884 (INPA, NY, RB, SPF, UFACPZ, UPCB) • Mun. Manoel Urbano, Parque Estadual Chandless, 180 m, 3 Apr 2019, Medeiros et al. 3810 (NY, RB, SPF, UFACPZ, US) • Mun. Sena Madureira, RESEX Cazumbá-Iracema, 3 Jan 2019, Medeiros et al. 3792 (RB, SPF, UFACPZ, US). **Colombia. Meta** • Centro de Investigaciones Ecológicas La Macarena, Apr 2000, Stevenson 2144 (NY). **Ecuador. Los Rios** • Bet Quevedo and Naranjal, 90 m, 7 Nov 1934, Mexia 6675 (F). **Napo** • 5 km of Las Sachas, 300 m, Baker et al. 5993 (QAME, QCNE, MO, NY) • Yasuní Forest Reserve, 240–310 m, 29 Jun 1995, Acevedo-Rodríguez and Cedeño 7577 (F, MO, US) • Estación Científica Yasuní, 200–300 m, 8 Nov 1997, Romoleroux and Baus 3200 (QCA, US) • Parque Nacional Yasuní, 200–300 m, 21 Jan 1998, R. J. Burnham and A. Krings 1569 (QCNE, F, MICH, US) • Estación Biológica Jatun Sacha, 450 m, 17 Feb 1988, Cerón 3657 (QCNE, MO, US) • Aguarico. Reserva Etnica Huaorani, 240 m, Aulestia 3367 (QCNE, MO, US). **Zamora-Chinchipe** • Nangaritza Cantón, Parroquia Zurmi, 1000 m, 13 Dec 2001, Clark et al. 6466 (QCNE, US). **Peru. Ayacucho** • Río Apurimac Valley, near Kimpitiriki, 400 m, 10 May 1929, Killip and Smith 22969 (F, NY). **Cuzco** • Prov. Convención, Echarate, 900m, 3 Feb 1939, Sork et al. 10496 (F). **Huánuco** • Vicinity of Tingo María, 10 Aug 1959, Mathias and Taylor 3991 (F, USM) • Ca. 30 km, SW of Pucallpa-Tingo María road, 300 m, Gentry and Diaz 58605 (F, MO, NY, USM). **Junín** • Between Azupizu and Santa Rosa, 650 m, 28 Jun 8 Jul 1929, Killip and Smith 26139 (NY) • Puente Perene, 600 m, 9 May 1961, Schunke-Vigo 4104 (MO). **Pasco** • Prov. Oxapampa, Carretera Oxapamapa-Paucartambo, 730 m, 11 Jun 2003, Rojas et al. 1147 (HOXA, MO, USM). **San Martín** • Prov. Chazuta, Río Huallaga, 260m, Apr 1935, Klug 4058 (F, MO, NY) • Prov. Juan Jui, Alto Río Huallaga, 400–800 m, Apr 1936, Klug 4293 (F, NY, USM) • Prov. Pachiza, Río Huayabamba, 1 Aug 1959, Mathias and Taylor 3980 (F, USM) • Prov. San Martín, Quebrada Mamonaquinha to junction with Río Mayo, 250 m, 25 May 1986, Knapp et al. 7405 (MO, USM) • Saposoa, Monte Real, 400 m, 7 Jul 1958, Woytkowski 5065 (MO). **Ucayali** • Middle Ucayali, Boca de Yarina, s.d., Tessmann 3500 (NY) • Purús, Río la Novia, 22 Feb 2002, Schunke-Vigo and Graham 14868 (MO, USM).

#### ﻿6. Thinouia paraguayensis

Taxon classificationPlantaeSapindalesSapindaceae

(Britton) Radlk. in Engler & Prantl, Nat. Pflanzenfam. 3(5): 308. 1895

1FD99935-F063-5881-9E2D-6B86DE3E8FDE

Figs 14 , 19A

Thouinia paraguayensis Britton, Ann. New York Acad. Sci. 7: 75. 1893
Thinouia sepium S. Moore, Trans. Linn. Soc. London, Bot. 4: 341. 1895. Type: Brazil. Mato Grosso. 1891–1892, *S. Moore 1076* (lectotype, designated here: BM [BM000838100] [image!]; syntype: Brazil. Mato Grosso. 1891–1892, *S. Moore 943*, B†).

##### Type.

**Paraguay** • Central Paraguay. Road to Lambare, 05 May 1889, *T. Morong 625a* (lectotype, here designated: NY! [NY02684301], isolectotypes: F [V0361740F] [image!], MO [MO101264008]).

**Figure 14.**
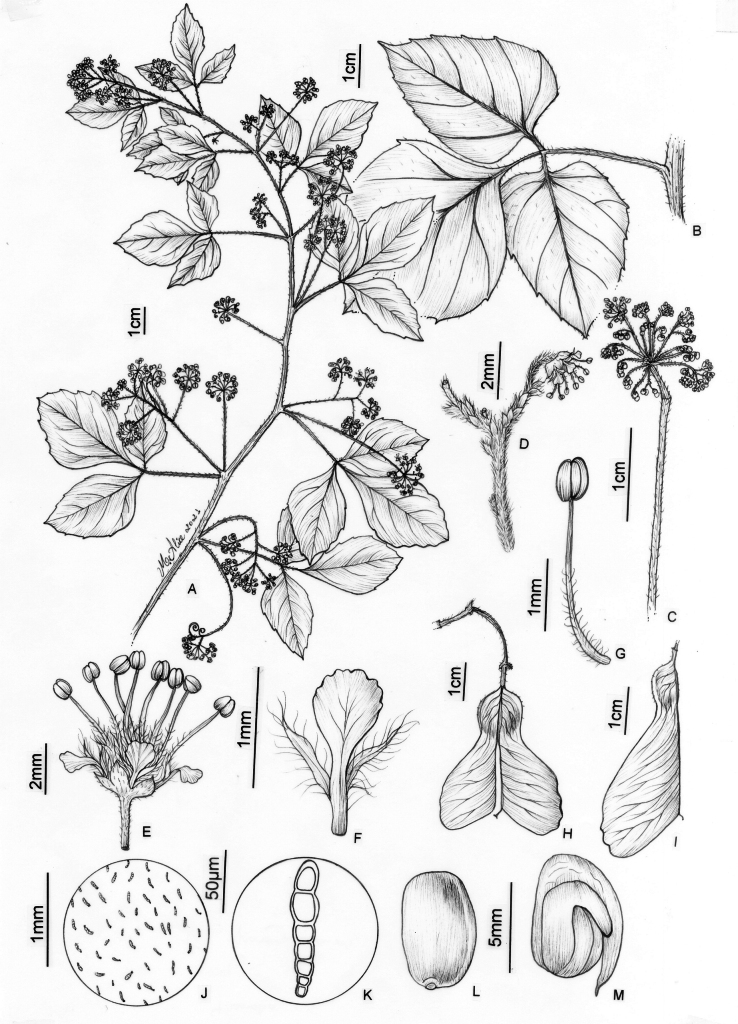
*Thinouiaparaguayensis* (Britton) Radlk. **A** portion of flowering branch **B** leaf **C** portion of Inflorescence **D** portion of inflorescence, displaying two cincinni and a staminate flower **E** staminate flower **F** petal with bifid appendage, dorsal [abaxial] view **G** stamen of staminate flower **H** fruit **I** mericarp **J** indumentum detail of locule cavity **K** capitate trichome with uniseriate stalk and unicellular terminal cells from locule cavity **L** seed **M** embryo (**A–G** from *Ferrucci 178***H–M** from *Hatschbach 49244*). Illustration by Maria Alice de Rezende.

##### Description.

Tendrilled liana; stem cylindrical, striate, puberulous, pubescent or tomentose, with yellowish to whitish indumentum, lenticels sparse, rounded or elliptic; cross-section simple or with neo formations when mature. Leaves trifoliolate; stipules minute, ca. 0.5 mm long, tomentose, deltoid; petiole 2.6–4.2 cm long, canaliculate, pubescent or tomentose; petiolules with keel in the middle, terminal petiolule 0.7 cm long, lateral petiolules 0.1–0.5 cm long; leaflets with the adaxial side puberulous, pubescent-tomentose only on the veins, the abaxial side pubescent or tomentose, discolorous; the leaflet secondary venation craspedodromous; secondary veins 4–5(6) pairs, opposite or alternate, spacing irregular, domatia sometimes present in abaxial surface of secondary vein axils; tertiary veins alternate percurrent; margins dentate-serrate, with 7–14 teeth per side, reduced to inconspicuous glands; terminal leaflet 4.4–6.5(10) × 2.5–5(8.5) cm, broadly ovate, apex obtuse or acuminate, mucronate, base decurrent or cuneate; lateral leaflets 3.5–4.5 × 1.8–4.4 cm, ovate, apex obtuse or acuminate, rarely retuse, mucronate, base truncate or rounded. Thyrses axillary or terminal, umbelliform, 1–6(7.5) cm long; peduncle 0.7–4(6) cm long; secondary peduncle (0)0.1–0.7 cm long; cincinni numerous, peduncle of cincinnus 1–3 mm long. Flower 3–5 mm long, pedicel ca. 2.3 mm long, villous; sepals ca. 0.5 mm long, connate at the base, deltate, abaxially villous, adaxially glabrous; petals ca. 1.6 mm long, spatulate, clawed, erose, adaxially glabrous or villous on the central part; appendages ca. 0.6 mm, shorter than the petals, bifid, sometimes bifurcate distally, villous; nectary disc annular, glabrous. Staminate flower with stamens 8, ca. 2.5 mm long, the filaments villous on lower half, the anthers papillose, glabrous, sometimes puberulous; pistillode ca. 0.6 mm long, villous at the apex. Pistillate flower with staminodes ca. 1 mm long, villous; pistil 2 mm long, puberulous or villous. Fruits chartaceous, 2.6–4.2 × 1.3–2.3 cm; accrescent pedicel 4.2–6.9 mm long; stipe 5.2–6.4 mm long; seed locule subglobose; epicarp glabrous or with sparse simple trichomes in the locule and ventral side of the wing; cavity of seed locule with sparse capitate trichomes; capitate trichomes with uniseriate stalk and unicellular terminal cells. Seed ellipsoid, 5.4–6.4 × 3.7–4.5 mm, basally attached, glabrous.

##### Distribution, habitat and phenology.

*Thinouiaparaguayensis* is known from tropical and subtropical dry broadleaf forests; tropical and subtropical grasslands, savannas, shrublands, and flooded grasslands and savannas in Bolivia, Brazil, and Paraguay (Fig. 19A), along roadside thickets, cerrado, chaco, gallery forests, and semi-deciduous forests. Flowering from December to April, and fruiting from January to August.

##### Notes.

*Thinouiaparaguayensis* is morphologically similar to *T.mucronata* and *T.ternata*. To distinguish it from *T.mucronata*, refer to the comments under that species. From *T.ternata* it is distinguished by the terminal leaflets that are <5.2 cm long (vs. >5.2 cm long), the lateral petiolules 0.1–0.5 cm long (vs. 2.1–4.4 cm long), the fruit 1.3–2.3 (vs. 2.5) cm wide), epicarp glabrous or subglabrous with sparse simple trichomes in the locule and ventral side of the wing (vs. glabrous).

In the protologue of the species (Morong and Britton 1893), Britton indicated the locality and number of specimens, but without specifying herbaria. The specimen NY02684301 is chosen here as the lectotype of *Thinouiaparaguayensis* (Britton) Radlk. The author of the basionym, Dr. Nathaniel Lord Britton, worked as the director-in-chief of the New York Botanical Garden and probably examined this specimen to describe the species.

For *Thinouiasepium*, a synonym of *T.paraguayensis*, the specimen *S. Moore 1076* (BM000838100) is chosen here as the lectotype. The description of this species was based on two collections (*S. Moore 943* and *1076*), both collections from the same place, however the collection *S. Moore 1076* has flowers and fruits and was the only collection we were able to locate.

##### Conservation status.

*Thinouiaparaguayensis* has an EOO of 403,484.82 km^2^ and an AOO of 148.00 km^2^. In central-eastern Brazil and Paraguay, where the species is found, the major threat affecting this species is to convert forest areas to agricultural land (large-scale plantations), and urban expansion. Despite this, the EOO values and the number of threat situations approach the thresholds for the inclusion of the species in a threat category. There are no data on population decline for the application of other criteria, thus, it should be regarded as Least Concern (LC).

##### Selected specimens examined.

**Bolivia. Santa Cruz** • Prov. Andrés Ibáñez, 7 km NW of Puerto Pailas, 300 m, 10 Feb 1994, Nee et al. 44866 (NY, US) • Prov. Chiquitos, Parque Histórico Santa Cruz La Vieja, 5 Apr 2006, Ferrucci et al. 2535 (CTES, UEC) • Prov. Cordillera, Cabezas, 20 Jan 1945, Peredo 22 (MO, NY) • Prov. Ñuflo de Chaves, Estancia San Miguelito, 260 m, 21 Dec 1995, Fuentes 1484 (MEXU) • Velasco, 58 km W de Roboré, 110 m, 19 Jul 2013, Ferrucci et al. 3128 (CTES, US). **Brazil. Mato Grosso** • Vicinity of Estancia Miranda, 22 Jun 1979, Prance et al. 26298 (CEN, NY). **Mato Grosso do Sul** • Mun. Corumbá, Baia de Tamengo, 8 Aug 1979, Claudio 454 (RB, UFACPZ, US) • Mun. Ladário, Estrada da Manga, 150 m, 3 Jun 1998, Damasceno Júnior et al. 1518 (COR) • Mun. Porto Murtinho, Estrada para o Rio Apa, 14 Jun 2006, Barbosa 1544 (HCF, HUEFS, MBM, MEXU). **Paraguay. [without Department**] • Cordillera de Altos, 1898–1899, Hassler 3745 (NY, P). **Central** • Estero del Ypoá, 10 Feb 1990, Zardini and Velázquez 18791 (US) • Jardín Botánico y Zoológico, 20 Jun 1990, Pérez 68 (US). **Cordillera** • Río Salado basin, 21 Jul 1990, Zardini and Velázquez 22320 (AS, MO, US) • Altos. 21 Mar 1990, Mereles 3842 (FCQ, MO). **Paraguarí** • Ruta 1.5 km N de Quindy, 29 Mar 1981, Ferrucci et al. 178 (F, MBM) • Cerro Mbatoví, 2 Jul 1988, Zardini 5433 (MO) • Cerro Palacios, 9 Jul 1988, Zardini 5651 (MO, US) • Acahay. Macizo Acahay, 5 Jan 1989, Zardini et al. 9207 (MO, PY, US). **San Pedro** • Alto Paraguay, 21 Jan 1961, Woolston et al. 1248 (HUEFS, SP).

#### ﻿7. Thinouia restingae

Taxon classificationPlantaeSapindalesSapindaceae

Ferrucci & Somner, Brittonia 60(4): 371. 2008

2259D64B-4DFD-5724-B1F9-4D39BD524C51

Figs 15 , 19A

##### Type.

**Brazil. Rio de Janeiro** • Município Saquarema, Reserva Ecológica Estadual de Jacarepiá, próximo à lixeira, 11 May 1994, *D. Araújo 10025* (holotype: RB! [724823], isotype: CTES [CTES0013570] [image!]).

**Figure 15.**
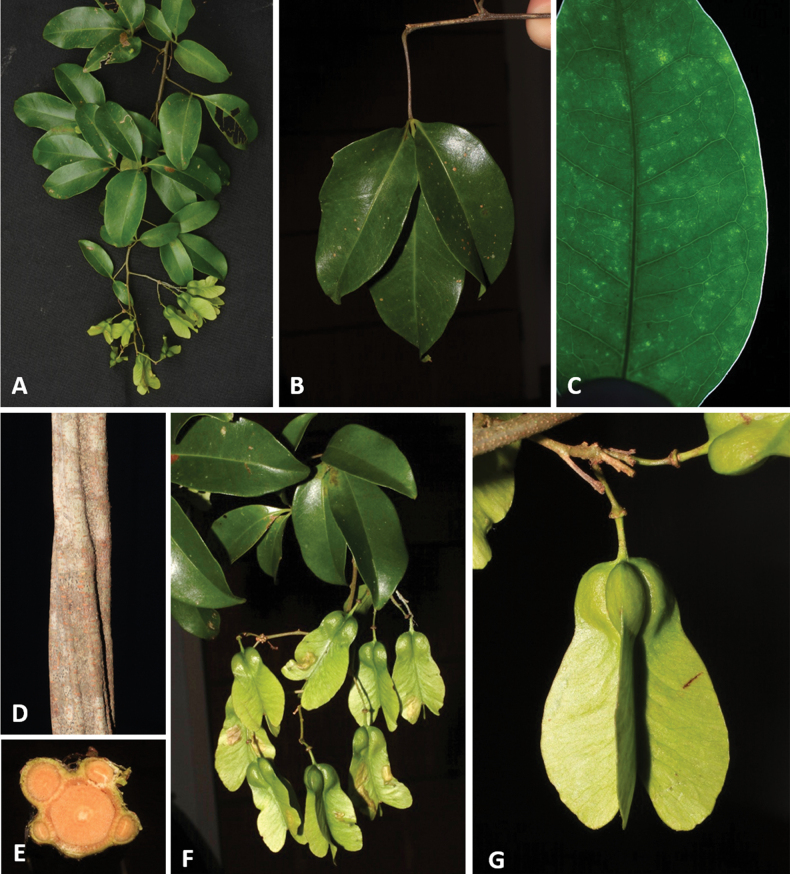
*Thinouiarestingae* Ferrucci & Somner **A** fertile branch **B** leaf **C** detail of the secondary veins and leaflet margin **D** lobed stem **E** cross-section of stem with neo formations **F** fruiting branch **G** mature schizocarp [Medeiros 4453 (**A–G**); photos: **A** by C. Toledo **B–G** by H. Medeiros].

##### Description.

Tendrilled liana; stem cylindrical-striate or 4–5 lobed, glabrous or puberulous, lenticels rounded and ferruginous; cross-section simple when young or with neo formations when mature. Leaves trifoliolate; stipules minute, ca. 0.5 m long, puberulous, triangular; petiole 1.5–4 cm long, canaliculate, glabrous, rarely puberulous; leaflets glabrous on both sides; the leaflet secondary venation brochidodromous; secondary veins 8–10 pairs, alternate, spacing irregular; intersecondaries present; tertiary veins irregular reticulate; margins entire, sometimes undulate and rarely with 1 tooth on the base; terminal petiolule 0.1–0.5 cm long, lateral petiolules 0.1–0.2 cm long; terminal leaflet 5–8 × 2.7–3.3 cm, oblong, symmetrical or asymmetrical, apex obtuse, rounded or emarginate, mucronate, base acute to decurrent; lateral leaflet 4.7 × 2.4 cm, oblong or oblong-ovate, asymmetrical, apex obtuse, rounded or emarginate, mucronate, base decurrent or rounded. Thyrses axillary or terminal, umbelliform, 0.8–2.8 cm long, peduncle 1–4.2 cm long, secondary peduncle subsessile 0.1–0.7 cm long, cincinni numerous, peduncle of cincinnus 0.2–0.8 cm long, tomentose to glabrescent. Flower 3–5 mm long, pedicel 1.4–4 mm long, glabrous; sepals ca. 1.3 mm long, deltoid, glabrous or sometimes glabrescent, ciliate; petals ca. 2 mm long, spatulate, erose at apex, clawed, adaxially glandular and with sparse simple trichomes; petal appendages 1–1.5 mm long, shorter than the petals, bifid, sometimes distally branched, villous; nectary disc annular, glabrous. Staminate flowers with stamens 8, ca. 4.8 mm long, filaments villous on lower half, anthers glabrous; pistillode ca. 0.3 mm long, villous. Pistillate flower with staminodes 8, ca. 2 mm long; pistil ca. 3 mm long, ovary adpressed-pubescent along dorsal edges, stigma and style puberulous. Fruits chartaceous, 3.6–4.2(5.4) × 2.6–3 cm; accrescent pedicel 3–4 mm long; stipe 3–6 mm long; seed locule lenticular; epicarp glabrous; cavity of seed locule densely villous with arachnoid, biseriate, simple or branched trichomes. Seed 6.8–7.3 × 5.2–5.6 mm, obovoid to ellipsoid, glabrous.

##### Distribution, habitat and phenology.

*Thinouiarestingae* is endemic to tropical and subtropical moist broadleaf forests in SE of Brazil, in restinga vegetation and ombrophilous and semi-deciduous forests in the states of Bahia, Espírito Santo, and Rio de Janeiro (Fig. 19A). Flowering from October to December, and fruiting from January to August.

##### Notes.

*Thinouiarestingae* and *T.ventricosa* have similar fruit morphology and are the only species with densely arachnoid trichomes on the locule cavity. *Thinouiarestingae* however, is distinguished from *T.ventricosa* by the entire or rarely basally 1-toothed margins (vs. dentate-serrate with 2–3(4) teeth), 8–10 pairs of secondary veins (vs. 3–5 pairs), secondary veins framework brochidodromous (vs. semicraspedodromous), and the absent of domatia (vs. domatia on abaxial side of secondary vein axil).

##### Conservation status.

*Thinouiarestingae* is represented by records distributed by the Atlantic Coast within an EOO of 95,642.60 km^2^ and AOO of 58.00 km^2^ in antropically modified restinga vegetation in the states of Rio de Janeiro and Espírito Santo, and less frequent in the semi-deciduous and ombrophilous forests in the states of Bahia, Espírito Santo and Rio de Janeiro. Thus, it should be regarded as Vulnerable [VU, B2ab(ii,iii,iv)], due to its range of distribution being less than 100 km^2^ and the number of locations being ≤10. Additionally, these species have a continually declining habitat quality, principally in the restinga vegetation where it faces intense pressure from human occupation.

##### Selecte specimens examined.

**Brazil. Bahia** • Mun. Jussarí, Rod. Jussarí/Palmira, 11 Feb 2003, Paixão et al. 211 (CEPEC). **Espírito Santo** • Mun. Aracruz, Retiro Serra Peladinha, 14 Dec 2007, Mansano et al. 480 (RB) • Mun. Nova Venécia, Área de Proteção Ambiental Pedra do Elefante, 18 Feb 2008, Forzza et al. 5089 (CEPEC, CTES, MBML, RB, UPCB) • Mun. Piúma, Estrada entre Marataízes e Piúma, 29 Nov 2006, Souza et al. 32487 (RB) • Mun. Sooretama, Reserva Biológica de Sooretama, 12 Feb 2021, Medeiros and Toledo 4475 (RB). **Rio de Janeiro** • Mun. Armação de Búzios, Praia Gorda, 16 Dec 1998, Fernandes et al. 195 (RB) • Mun. Cabo Frio, Parque Ecológico Municipal do Mico Leão Dourado, 29 May 2003, Fernandes et al. 855 (RB, RBR) • Mun. Campos dos Goytacazes, Morro do Itaoca, 361 m, 15 Feb 2021, Medeiros and Toledo 4453 (RB) • Mun. Maricá, Reserva Ecológica de Jacarepiá, 27 Feb 2021, Medeiros and Toledo 4491 (RB) • Mun. Rio das Flores, Estrada para Rio das Flores, 2 Aug 2006, Marquete et al. 3775 (RB, UFAPZ) • Mun. Rio das Ostras, Praia Virgem, 5 May 2016, Somner et al. 1826 (RB, RBR) • Mun. Saquarema, Reserva Ecológica Estadual de Jacarepiá, 28 Sep 1990, Somner et al. 616 (RB).

#### ﻿8. Thinouia scandens

Taxon classificationPlantaeSapindalesSapindaceae

(Cambess.) Triana & Planch., Ann. Sci. Bot. Ser. 4, 18: 369. 1862

CD9AA369-68BD-5666-836A-60CA0BD3031C

Figs 16 , 19B

Thouinia scandens Cambess. in A. de Saint-Hilaire, Flora Brasiliae Meridionalis 1: 384. 1828. Thinouiascandensformagenuina nom. invalid.
Paullinia caudata Vell., Fl. Fluminensis 159. 1829 (“1825”); *Fl. Fluminensis Icones 4: tab. 31*. 1831 (“1827”). Thinouiascandensformacaudata (Vell.) Radlk., Sitzungsber. Math.-Phys. Cl. Königl. Bayer. Akad. Wiss. München. 8(3): 282. 1878. (lectotype, designated here: Brasil. [Rio de Janeiro or São Paulo] no locality or habitat given; [illustration] Original parchment plate *Flora Fluminensis* in the Manuscript Section of the Biblioteca Nacional do Rio de Janeiro [cat. no.: mss1198653_029] and later published in Vellozo, *Fl. Fluminensis Icones 4: tab. 31*. 1831. [image!]).
Thinouia scandens forma racemosa Radlk., Sitzungsber. Math.-Phys. Cl. Königl. Bayer. Akad. Wiss. München. 8(3): 282. 1878. Thouiniamacroptera Casar., Nov. Stirp. Bras. 5: 45. 1843. Carpidiopterixmacroptera (Casar.) H. Karst., Fl. Columb. 2: 45. 1863. Type: Brazil. “Habitat circa Rio de Janeiro, s.d. [before 1840], Riedel s.n [*Casaretto Herb. no. 2479*] (lectotype, designated here: TO [image!]).
Thinouia scandens forma areolata Radlk. nom. nud.

##### Type.

**Brasil. Rio de Janeiro** • “Nascitur in sylvis primaevis provinciae Rio de Janeiro, “Florebat Februario”, *Saint-Hilaire s. n..* (lectotype, here designated: MPU [MPU010891] [image!]).

**Figure 16.**
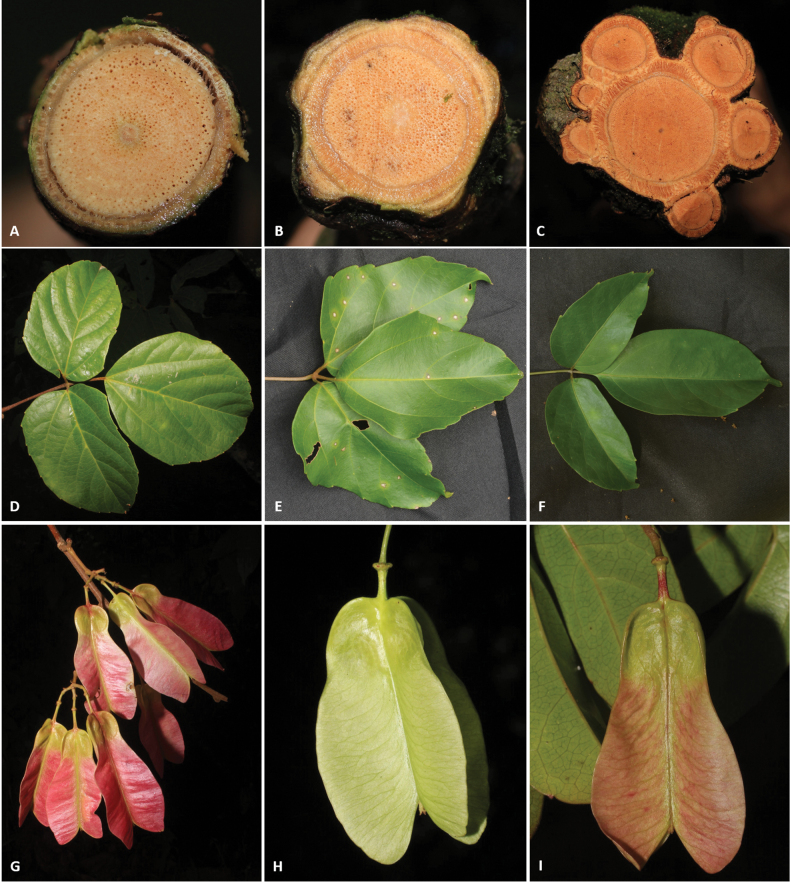
Morphological variation in *Thinouiascandens* (Cambess.) Triana & Planch. **A** stem cross-section cylindrical, simple **B** stem cross-section obtusely pentagonal, showing early neo formation **C** stem cross-section ribbed (lobed), showing neo formations at different stages of development **D–F** leaf morphological variation **G–I** fruit morphological variation [Medeiros 4450 (**E, H**) 4451 (**B**) 4473 (**A, D, G**) 4481 (**C**) 4486 (**F**) 4488 (**I**); photos: by H. Medeiros].

##### Description.

Tendrilled liana; stem cylindrical or 4–5 lobed, glabrous, pubescent or tomentose, lenticels rounded, elliptic or oblong; cross-section simple or with neo formations when mature. Leaves trifoliolate; stipules ca. 0.5 mm long, tomentose, triangular; petiole 5.6–9.1 cm long, semiterete to canaliculate with keel in the middle, striate, glabrous to pubescent; terminal petiolule 0.8–3.7 cm long, canaliculate with keel in the middle, striate; lateral petiolules with keel in the middle 0.4–1.4 cm long; leaflets with the adaxial side glabrous and the abaxial side glabrous, glabrescent, puberulous or tomentose; the leaflet secondary venation craspedodromous to semicraspedodromous, secondary veins 5–7 pairs, alternate or subalternate, spacing irregular, domatia on abaxial surface of secondary vein axils commonly on lowermost pair of secondary veins; intersecondaries present; tertiary veins alternate percurrent or mixed opposite-alternate percurrent; margins entire, dentate or serrate with 3–9 teeth, reduced to inconspicuous glands; terminal leaflet 5.2–11.7 × 3.1–7 cm, obovate, oblong, oblong-ovate or ovate to very widely ovate, symmetrical or asymmetrical, apex rounded, obtuse, acute or acuminate, mucronate or sometimes emarginate, base rounded to obtuse; lateral leaflets 4.3–12.7 × 2.7–8.1 cm, ovate, oblong, elliptic or lanceolate, asymmetrical, the apex rounded, obtuse, acute or acuminate, mucronate, base truncate or obtuse to rounded. Thyrses axillary or terminal, umbelliform, 2–4 cm long, peduncle 1.5–2 cm long, secondary peduncle 0.2.–0.5 cm long; cincinni numerous, peduncle of cincinnus 0.6–0.7 cm long, tomentose. Flower 4.5–6 mm long; pedicel 2–3 mm long, pilose or sparsely pubescent; sepals ca. 0.5 mm long, oblong-ovate, abaxially pilose or sparsely pubescent, adaxially glabrous or sometimes villous, ciliate; petals 1.6–2.5 mm long, oblong-spatulate, clawed, erose, adaxially glandular, abaxially glabrous; appendages 0.7–1.5 mm long, shorter than the petals, villous; nectary disc annular, glabrous. Staminate flowers with stamens 8, 2.8–3 mm long, the filaments villous on lower half, the anthers 0.5–0.6 mm long, glabrous, papillose; pistillode ca. 0.5 mm villous. Pistillate flower with staminodes 8, 1.5–1.7 mm long, villous throughout; pistil ca. 4.5 mm long, the ovary adpressed-pubescent along dorsal edges, the stigma and style tomentose. Fruits chartaceous, 4.3–7.5 × 2.5–4.1 cm; accrescent pedicel 2.3–6.1 cm long; stipe 4.3–9.7 cm long; seed locule subglobose to lenticular; epicarp glabrous; cavity of seed locule pubescent with capitate trichomes; capitate trichomes with uniseriate stalk and multicellular terminal cells. Seed ellipsoid, 6–7.4 × 4.3–5.6 cm, glabrous.

##### Distribution and habitat.

*Thinouiascandens* is endemic to tropical and subtropical moist broadleaf forests in SE Brazil, between 40–1200 m, in gallery forests, semi-deciduous forests, *tabuleiro* forests and ombrophilous forests in Bahia, Espírito Santo, Minas Gerais, São Paulo and Rio de Janeiro states (Fig. 19B). Flowering from December to April and fruiting from January to October.

##### Notes.

*Thinouiascandens* is easily recognized by the leaflets with entire, dentate or serrate margins, with 3–9 pairs teeth per side or with teeth reduced to inconspicuous glands; venation craspedodromous to semicraspedodromous; fruits 4.3–7.5 cm long; and locule cavity densely pubescent with capitate trichomes. Radlkofer (1878, 1931–1934) recognized several taxonomic forms within *T.scandens* based on variations of leaf size, form and pubescence. However, our study found that these characters overlap. Additionally, in our molecular phylogenetic studies the samples representing *T.scandens* were recovered with very strong support in a clade. Therefore, we considered the forms proposed by Radlkofer as synonyms of *T.scandens*.

In the protologue of the *Thinouiascandens* (Cambess.) Triana & Planch. only the place of collection was indicated without mentioning a particular specimen. We have found three specimens that correspond to the collector and identified by the basionym species author: MPU010891, P00754921, and P00754922. Of these, only in the former does the locality correspond with that of the protologue and therefore has been chosen as the lectotype. For the name *Paulliniacaudata* Vell., we have selected as the lectotype the illustration of Vellozo (1831, t. 31), which is the only original material available for this name, *Paulliniacaudata*. This illustration clearly fits in the *Thinouiascandens* due its elliptic to ovate leaves with margins entire to dentate-serrate and the long fruits. Thinouiascandensf.racemosa was based upon two elements, i.e., the illegitimate *Paulliniaracemosa* of Vellozo (1831, t. 29) and a collection of Riedel s.n. (the type of *Thouiniamacroptera* Casar.). We have selected the latter as the lectotype, as it is preferable to an illustration.

##### Conservation status

. *Thinouiascandens* possesses a broad EOO of 180,117.85 km^2^ and an AOO of 136.00 km^2^, with many known locations. However, the ombrophilous forest vegetation in SE Brazil, where the species is found, is subject to continuing decline in area and quality of habitat due to anthropic pressure. Despite this, there are no data on population decline to apply other criteria and several conservation units in Brazil protect the species. Thus, it should be regarded as Least Concern (LC).

##### Selected specimens examined.

**Brazil. Bahia** • Mun. Almadina, Serra do Corcovado, 20 Mar 2006, Paixão et al. 909 (CEPEC, RBR) • Mun. Nova Viçosa, Estrada para Nova Viçosa, 22 Feb 2021, Medeiros and Toledo 4486 (RB, SPF, UFACPZ) • Mun. Porto Seguro, Estação Veracel, 12 Oct 2006, Colman et al. 21 (ALCB) • Mun. Una, Reserva Biológica do Mico-Leão, 10 Mar 1993, San’t Ana et al. 291 (CEPEC, SP). **Espírito Santo** • Mun. Alegre, São João do Norte, 17 Mar 2009, Couto et al. 1131 (MBML, VIES) • Mun. Linhares, Estrada paralela ao Rio Doce, 18 Feb 2021, Medeiros and Toledo 4472 (RB, SPF, UFAPCPZ) • Mun. Nova Venécia, Área de Proteção Ambiental Pedra do Elefante, 329 m, Fraga et al. 2083 (CEPEC, CTES, MBML, RB, UPCB) • Mun. Regência, Reserva Biológica de Comboios, 24 Jan 1990, Folli 1076 (US) • Mun. São Mateus, Próximo de Boa Esperança, s.d, Maguanini and Mattos s. n.. (RB 87950). **Minas Gerais** • Mun. Alto Caparaó, Parque Nacional do Caparaó, 23 Apr 1998, Leoni 3950 (RB) • Mun. Carangola, PCH-Carangola, Feb 2007, Leoni et al. 63 (RB, UFACPZ) • Mun. Faria Lemos, Fazenda Santa Rita, 14 May 2006, Silva 122 (RB) • Mun. Francisco Sá, ca. 5 km NE of Francisco de São, 950 m, Irwin et al. 23129 (MG) • Mun. Ipaba, s.d., Pujals s. n.. (MBM 420905). **Rio de Janeiro** • Mun. Guapimirim, Parque Nacional da Serra do Órgãos, 734 m, 14 Feb 2021, Medeiros and Toledo 4452 (NY, RB, SPF, UFACPZ, US) • Mun. Itaperuna, Estrada para São Lourenço, 7 Jun 2004, Marquete et al. 3501 (IBGE, HRB) • Mun. Mangaratiba. Trilha para o Mirante, 17 Aug 2017, Somner and Acevedo-Rodríguez 1851 (RBR) • Mun. Natividade, Mata de São Vicente, Morro da Torre, 11 Jun 2006, Gonçalves et al. s. n.. (RB 00594985) • Mun. Niterói, Morro do Cavalhão, 28 Aug 1888, Glaziou et al. 17499 (P) • Mun. Nova Friburgo, Macaé de Cima, 9 Oct 1993, Vieira and Gurken 429 (ESA, MBM, NY, RB) • Mun. Paraty, Estrada para o morro do Corisquinho, 10 Mar 1994, Campos 14 (CTES, RB, RBR) • Mun. Petrópolis, Estrada velha Rio Petrópolis, 1 Sep 1990, Somner et al. 585 (RBR) • Mun. Piraí, Distrito de Cacaria, 27 May 2009, Somner et al. 1339 (RBR) • Mun. Rio Bonito, Distrito de Brasília, Fazenda Cachoeira, 13 Aug 1986, Acevedo-Rodríguez et al. 1436 (MO, NY, RB) • Mun. Rio de Janeiro, Horto Florestal, 28 Apr 1924, Florestal s. n.. (RB 148992, S) • Mun. Teresópolis, Parque Nacional da Serra dos Órgãos, 14 Feb 2021, Medeiros and Toledo 4450 (NY, RB, SPF, UFACPZ, US). **São Paulo** • Equipe Morro das Pedras, 1917, Brade 7923 (R) • Mun. São Paulo. Nativa do Jardim Botânico, 25 Feb 1942, Hoehne s. n.. (SP 46367).

#### ﻿9. Thinouia silveirae

Taxon classificationPlantaeSapindalesSapindaceae

H. Medeiros sp. nov.

26B772B1-795C-524A-9070-E3DDFA6D1C22

urn:lsid:ipni.org:names:77356941-1

Figs 17 , 18 , 19B

##### Diagnosis.

*Thinouiasilveirae* is most closely related to *T.myriantha* but differs from the latter by its mericarps with lenticular locule that are flat at the base, the pubescent epicarp and the densely villous locule cavity with capitate trichomes, and the petal appendage adnate to the marginal portion (vs. mericarps with slightly flattened locule; epicarp glabrous, locule cavity sparsely pilose or glabrous with scattered capitate trichomes, and petal appendage adnate to the basal portion).

**Figure 17.**
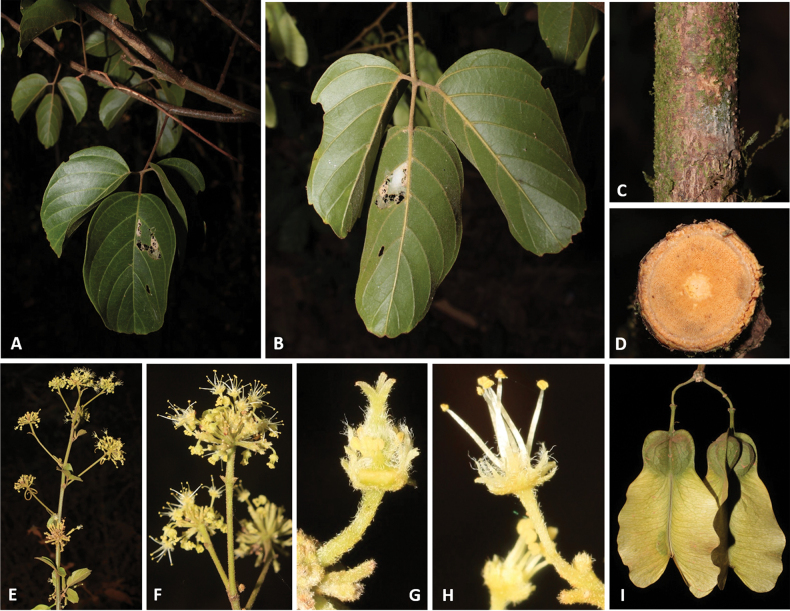
*Thinouiasilveirae* H. Medeiros **A** vegetative branch **B** leaf showing abaxial surface **C** stem with lenticellate bark **D** stem cross-section **E** distal synflorescence **F** distal portion of synflorescence **G** mature fruits **H** staminate flower **I** pistillate flower [Medeiros 2191 (**A–D, G**) 4496 (**E–F, H–I**); photos by H. Medeiros].

##### Type.

**Brazil. Acre** • Mun. Xapuri. Ramal que dá acesso a pousada do Seringal Cachoeira, 9 Jul 2021, *H. Medeiros, C. G. Silva and M. H. Oliveira 4496* (holotype: RB!, isotypes: INPA!, NY!, SPF!, RON!, UFACPZ!, US!).

**Figure 18.**
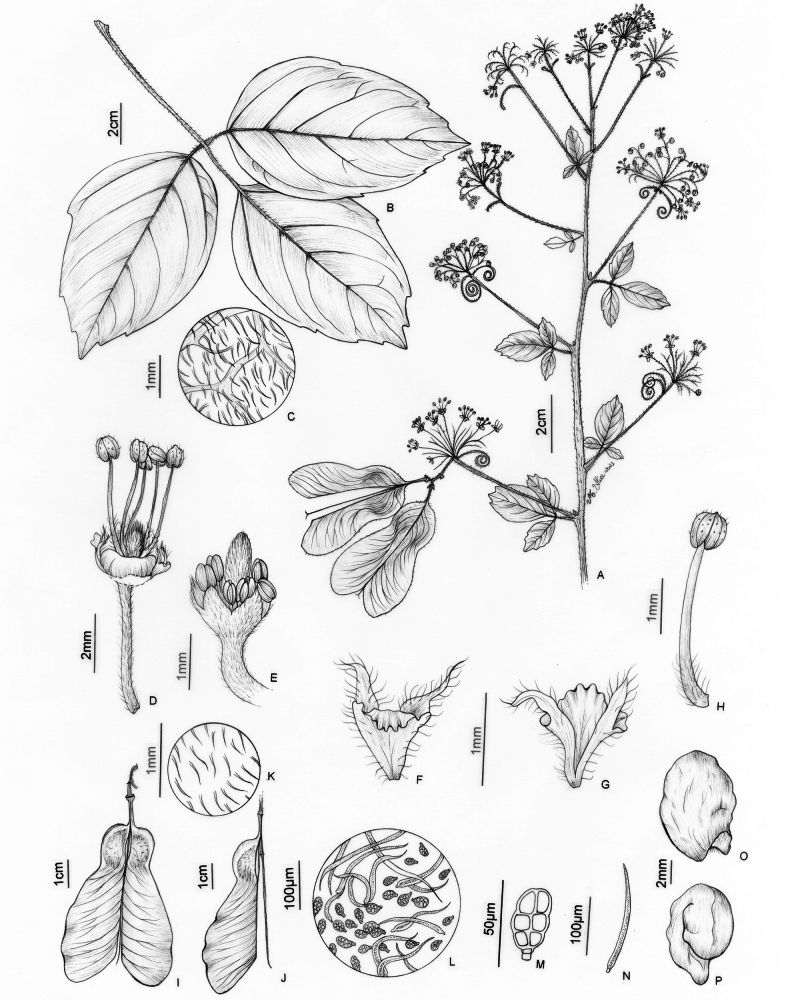
*Thinouiasilveirae* H. Medeiros **A** portion of flowering branch **B** leaf **C** indumentum on leaflets **D** staminate flower with portion of perianth removed showing nectary disc **E** pistillate flower **F** petal with bifid and marginal appendage, dorsal [abaxial] view **G** petal with bifid appendage, frontal [adaxial] view **H** stamen of staminate flower **I** fruit **J** mericarp **K** indumentum of epicarp **L** indumentum detail of locule cavity **M** capitate trichomes with uniseriate stalk and multicellular terminal cells from locule cavity **N** simples trichomes on locule cavity **O** seed **P** embryo (**A–H** from *Medeiros 4496***I–P** from *Medeiros 2191*). Illustration by Maria Alice de Rezende.

##### Description.

Tendrilled liana, 10–15 m long; stem 3–5 cm diam., cylindrical, striate, tomentose, glabrescent when mature, lenticels rounded or elliptic; cross-section simple. Leaves trifoliolate; stipules ca. 1 mm long, tomentose, triangular; petiole 5.8–12.4 cm long, terete or angular, striate, tomentose or pubescent; terminal petiolule 2–4.5 cm long, semiterete or terete, striate; lateral petiolules 0.7–1.9 cm long; leaflets with adaxial side glabrous or glabrescent, sometimes pubescent along veins, the abaxial side glabrescent, puberulous or pubescent; the leaflet secondary venation eucamptodromous but semicraspedodromous toward the apex; secondary veins 5–6(7) pairs, alternate or subalternate, spacing irregular, with domatia on the abaxial side of secondary vein axils; intersecondaries present; tertiary veins alternate percurrent; margins entire or sparsely dentate, with 3–6 vestigial teeth reduced to inconspicuous glands; terminal leaflet 8.6–16.6 × 6–11.6 cm, elliptic-ovate or ovate, symmetrical or asymmetrical, the apex acuminate, sometimes emarginate, the base rounded; lateral leaflet 7.8–15.6 × 5.7–10 cm, elliptic or ovate, asymmetrical, the apex acuminate, sometimes rounded to retuse, the base truncate or slightly cordate. Thyrse umbelliform, axillary or terminal; solitary when axillary, 3.5–5.5 cm long, with a pair of circinate tendrils on distal portion of peduncle; peduncles 1.5–3.5 cm long; secondary peduncles 0.1–0.7 cm long; cincinni numerous, peduncle of cincinnus 1.7–4.7 mm long. Flowers 2.5–7.3 mm long, pedicel 1.2–3.5 mm long, pilose to villous; sepals ca. 0.7 mm long, connate at the base, deltate, abaxially villous, adaxially glabrous; petals ca. 0.5 mm long, obdeltoid to widely obtrullate, not clawed, villous; appendages ca. 0.9 mm long, longer than the petals, marginal and bifid, villous; nectary disc glabrous, annular. Staminate flower with stamens 8, ca. 2.7 mm long, the filaments villous at the base, the anthers ca. 0.5 mm long, papillose, glabrous or puberulous; pistillode ca. 0.5 mm long, villous. Pistillate flower with staminodes 0.8–1.2 mm long, villous; pistil ca. 1.5 mm long, villous. Fruits chartaceous, 4–6.1 × 2.5–3.9 cm; accrescent pedicel 2.6–5.3 cm long; stipe 4.2–9.4 cm long; seed locule lenticular but flattened at the base; epicarp pubescent at the locule, puberulous or pilose at the wing; cavity of seed locule densely villous, with capitate trichomes with uniseriate stalk and multicellular terminal cells. Seed ellipsoid 7.5–9.5 × 4.1–6.2 mm, glabrous.

##### Distribution and habitat.

**Figure 19.**
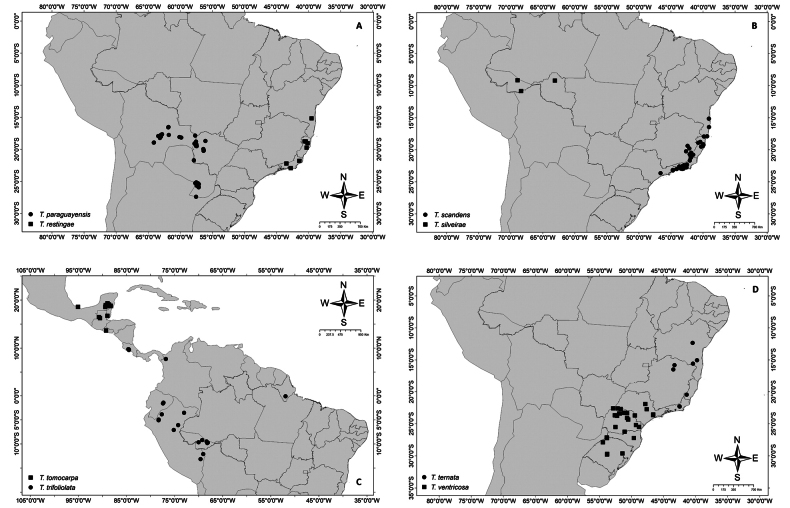
Distribution maps of *Thinouia* species **A***Thinouiaparaguayensis* and *T.restingae***B***T.scandens* and *T.silveirae***C***T.tomocarpa* and *T.trifoliolata***D***T.ternata* and *T.ventricosa*.

*Thinouiasilveirae* is known only from the southwestern Amazonian region, in the states of Acre and Rondônia, Brazil (Fig. 19B); in tropical and subtropical moist broadleaf forests. Flowering from June to July, and fruiting from July to September.

##### Etymology.

The specific epithet honors Dr. Marcos Silveira, professor and ecologist, at the Universidade Federal do Acre, who has made significant contributions to the floristic studies of Acre and SW Amazonia.

##### Notes.

*Thinouiasilveirae* is strongly supported as sister to *T.myriantha* (Fig. 4) by our phylogenetic analyses of molecular data. In addition, both species share similar morphology, including elliptic to ovate leaflets, the presence of domatia, leaflet margins that are dentate to serrate, an annular and glabrous disc, and petal appendages that are longer than the petals (Fig. 5). *Thinouiasilveirae* is differentiated from this close relative by its mericarps which have: (1) inflated, pubescent cocci that are flattened at the base; (2) wings that are puberulous or pilose; and (3) a densely villous seed locule cavity with simple and capitate trichomes.

##### Conservation status.

*Thinouiasilveirae* possesses a broad EOO of 59,955.343 km^2^ and an AOO of 12.00 km^2^. Although this species is known only from a few collections, its conservation status is here treated as Least Concern (LC) due to its occurrence within two conservation units, the Reserva Extrativista do Cazumbá-Iracema in the state of Acre, and the Floresta Nacional do Jamari in the state of Rondônia.

##### Selected specimens examined.

**Brazil. Acre** • Mun. Sena Madureira, RESEX Cazumbá-Iracema, 31 Jul 2017, Medeiros et al. 2189 (RB, SPF, UFACPZ, US) • Mun. Xapuri, Estrada para o Seringal Cachoeira, 8 Sep 2017, Medeiros et al. 2193 (INPA, RB, SPF, UFACPZ). **Rondônia** • Mun. Itapoã do Oeste, Floresta Nacional do Jamari, 28 Aug 2017, Medeiros et al. 2191 (RB, RON, SPF, UFACPZ, US).

#### ﻿10. Thinouia ternata

Taxon classificationPlantaeSapindalesSapindaceae

Radlk., Sitzungsber. Math.-Phys. Cl. Königl. Bayer. Akad. Wiss. München 8: 282. 1878

2C59C367-64F5-5CE6-83B1-47B0616B674D

Figs 19D , 20 , 21

Banisteria ternata Vell., Fl. Fluminensis: 159. 1829 (“1825”) (nom. illeg.).

##### Type.

**Brazil. Minas Gerais** • Lagoa Santa, s.d. *Warming s. n..* (lectotype, designated here: P [P06695484] [image!] – epitype, designated here: Brazil. Bahia. Rui Barbosa. ARIE Serra do Orobó, Fazenda Bom Jardim [12°19'43"S, 40°28'34"W] 591 m, 21 Apr 2006, *D. Cardoso and K. S. Matos 1245* RB! [RB01464762]; isoepitype: NY!, HUEFS [HUEFS108414] [image!]).

**Figure 20.**
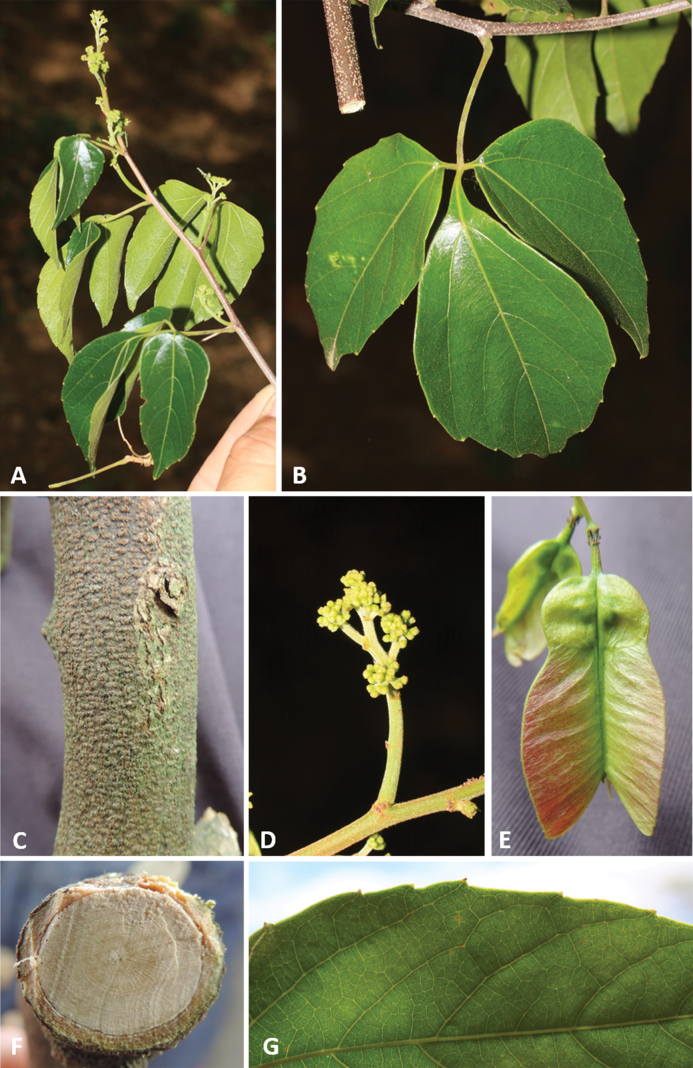
*Thinouiaternata* Radlk. **A** fertile branch **B** leaf **C** stem with lenticellate bark **D** portion of young synflorescences **E** mature fruit **F** stem cross-section, simple **G** partial view of leaflets showing secondary veins and serrulate margins [Medeiros 4489 (**A, B, D, G**); Daneu 746 (**C, E–F**); photos: **A, B, D, G** by H. Medeiros **C, E, F** by L. Daneu].

##### Description.

Tendrilled liana; stem pubescent or tomentose and glabrescent when mature, lenticels round or elliptic; cross-section simple. Leaves trifoliolate; stipules ca. 0.5 mm long, tomentose, triangular; petiole 3.5–7.7 cm long, terete or semiterete and keeled along the middle, striate, pubescent to tomentose; terminal petiolule 0.3–0.7 cm long, semiterete; lateral petiolules 0.2–0.5 cm long; leaflets with adaxial side tomentose to glabrescent, sometimes strigose, the abaxial side tomentose; leaflet secondary venation craspedodromous; secondary veins 4–6 pairs, alternate or subalternate, spacing irregular, with domatia on the abaxial side of secondary veins axils; intersecondaries present; tertiary veins mixed opposite-alternate percurrent or alternate percurrent; margins dentate or serrate, with (7)8–12 vestigial teeth on each side, reduced to inconspicuous glands; terminal leaflet 5.3–10.6 × 4.4–5.6 cm, broadly elliptic, obtrullate or ovate, the apex acuminate and mucronate, the base decurrent; lateral leaflet 4.2–8.9 × 2.3–3.8 cm, ovate, asymmetrical, the apex acute to acuminate, mucronate, the base truncate to rounded. Thyrses axillary or terminal, umbelliform, 1.5–5.3 cm long; peduncle 0.5–4.2 cm long; secondary peduncle 0.1–0.5 cm long; cincinni numerous, peduncle of cincinnus 2–3 mm long, tomentose. Flower 5–5.2 mm long, pedicel 2–3 mm long, pilose or sparsely pilose; sepals ca. 0.5 mm long, triangular, abaxially pilose or sparsely pubescent, adaxially glabrous or sometimes villous, ciliate; petals 1–1.7 mm long, oblanceolate, spatulate, clawed, erose, adaxially villous and abaxially glabrous; petal appendages <0.5 mm long, shorter than the petal, bifid, villous; nectary disc annular, glabrous. Staminate flowers with stamens 8, ca. 2.5 mm long, the filaments villous on lower half, the anthers ca. 0.5 mm long, glabrous, papillose; pistillode ca. 0.4 mm long, villous. Pistillate flower with staminodes 8, ca. 1 mm long, the filaments villous ca. on lower half, the anthers ca. 0.3 mm, glabrous, papillose; pistil ca. 4 mm long, glabrous at base, villous from middle to apex. Fruits chartaceous, 3–4 × 2.5 cm; accrescent pedicel 4.7–5.2 cm long; stipe 6.2–7 mm long; seed locule lenticular; epicarp glabrous; cavity of seed locule glabrous or with sparse simple or capitate trichomes with uniseriate stalk and unicellular terminal cells. Seed ellipsoid 5.7–7 × 3.6–4.7 cm, glabrous.

**Figure 21.**
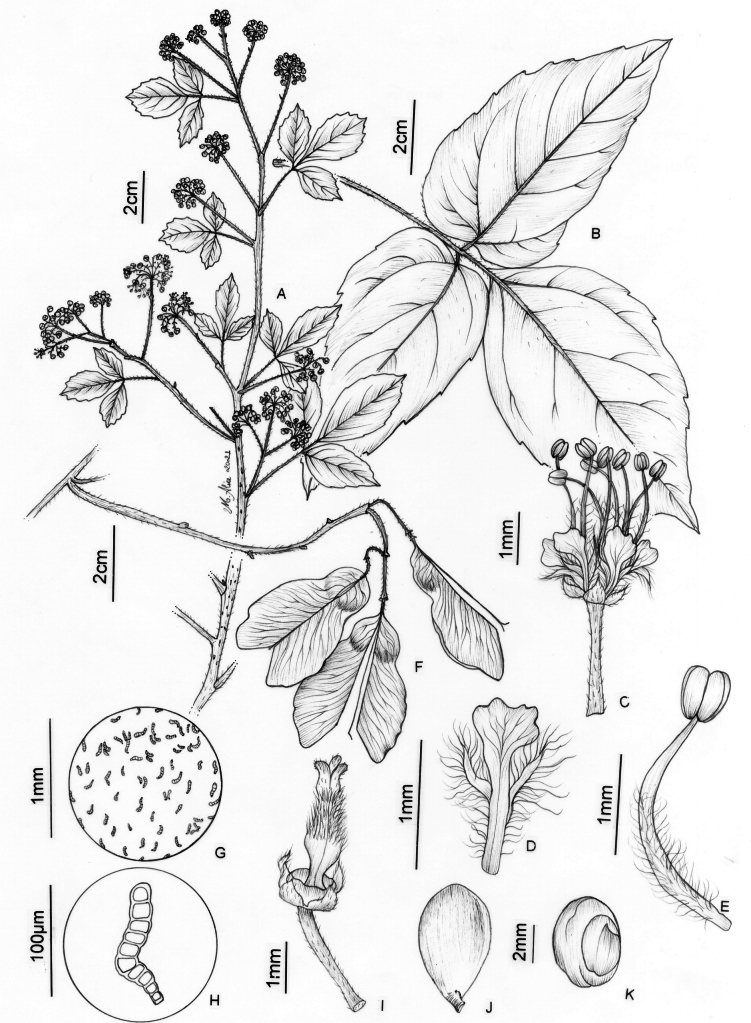
*Thinouiaternata* Radlk. **A** flowering branch **B** leaf **C** staminate flower **D** petal with bifid appendage, dorsal [abaxial] view **E** stamen of staminate flower **F** infructescence **G** indumentum detail of locule cavity **H** capitate trichomes with uniseriate stalk and unicellular terminal cells from locule cavity **I** pistillate flower with portion of perianth removed showing the gynoecium and nectary disc **J** seed **K** embryo (**A–E** from *Amorim 3580***H–M** from *Cardoso 1245*). Illustration by Maria Alice de Rezende.

##### Distribution and habitat.

This species is endemic to tropical and subtropical Brazil, found in moist broadleaf forests in ombrophilous, semi-deciduous and deciduous forests, and along streams, on limestone outcrops, and forest margins, in Bahia, Espírito Santo, Minas Gerais and Rio de Janeiro states (Fig. 18D), at 215–950 m elevation. Flowering from February to April, and fruiting from May to October.

##### Notes.

*Thinouiaternata* is sister to *T.mucronata* and *T.paraguayensis* (see comments under *T.paraguayensis*) (Fig. 4). They are morphologically similar, with simple stems, leaflets with mucronate apex, flowers 3–5 mm long, and fruits chartaceous. *Thinouiaternata* can be distinguished from *T.mucronata* by the lateral leaflet that are truncate to rounded at the base (vs. decurrent) and by the craspedodromous (vs. semi-craspedodromous) venation.

*Banisteriaternata* Vell. is the older name for this species. However, as it is a later homonym of *Banisteriaternata* DC., therefore the name is illegitimate. Consequently, the name *Thinouiaternata* should be credited to Radlkofer (1878), not a combination based on Vellozo’s name. In the protologue of *T.ternata* Radlk., Radlkofer cited a collection of Warming from Minas Gerais in addition to the name of Vellozo. We designate the collection of Warming deposited at P as the lectotype, as a specimen is preferable over an illustration. Additionally, we designated an epitype because the lectotype does not have the necessary features in order to fix this name with certainty to a given species.

##### Conservation status.

*Thinouiaternata* is represented by a few records from four states of Brazil’s Atlantic Forest, with an EOO of 264,322.39 km^2^ and an AOO of 28.00 km^2^ in the anthropically modified semi-deciduous forests. Thus, it should be regarded as Vulnerable [VU, B2ab(ii, iii, iv)], due to its range of distribution being less than 100 km^2^ and the number of locations being ≤10. Additionally, these species have continuously declining habitat quality, principally in the restinga vegetation where it faces intense pressure from human occupation.

##### Selected specimens examined.

**Brazil. Bahia** • Mun. Itabuna, Margem do Rio Pardo, 23 May 1968, Belém 3597 (CEPEC, F, IAN, NY) • Mun. Itajú do Colônia, ca. 81km de Santa Cruz da Vitória, 25 Feb 2021, Medeiros and Toledo 4489 (CEPEC, RB, SPF, UFACPZ, US). **Espírito Santo** • Mun. Muniz Freire, Vieira Machado, 17 Feb 1993, Souza 439 (CVRD). **Minas Gerais** • Serra do Espinhaço, ca. 5 km NE of Francisco de Sá, 13 Feb 1969, Irwin et al. 23219 (NY, UB) • Mun. Janaúba, Ribeirão Poções, 13 Feb 1991, Hatschbach 55100 (MBM) • Mun. Pains, 613 m, 12 Jul 2006, Borges et al. 224 (RB, RBR) • Fazenda Amargoso, 750–810 m, 25 May 2003, Melo et al. 625 (BHCB, RB). **Rio de Janeiro** • Mun. Nova Friburgo, caminho para a Pedra do Cão Sentado, 27 Oct 1986, Somner et al. 544 (RBR).

#### ﻿11. Thinouia tomocarpa

Taxon classificationPlantaeSapindalesSapindaceae

Standley, Field Mus. Nat. Hist., Bot. Ser. 12: 411. 1936

83FF50B6-857F-5AF8-89C1-0FDB1D2C1598

Figs 19C , 22

##### Type.

**Belize** • Temash River, 6 Feb 1935, *W. A. Schipp 1336* (holotype: F [0361405F] [image!], isotypes: G [G00008257] [image!], G [G0008262] [image!], G [G00008260] [image!], G [G00008261] [image!], MICH [MICH1115487] [image!], K [K000634088] [image!], NY! [NY00387405], S [S-R-11015] [image!]).

**Figure 22.**
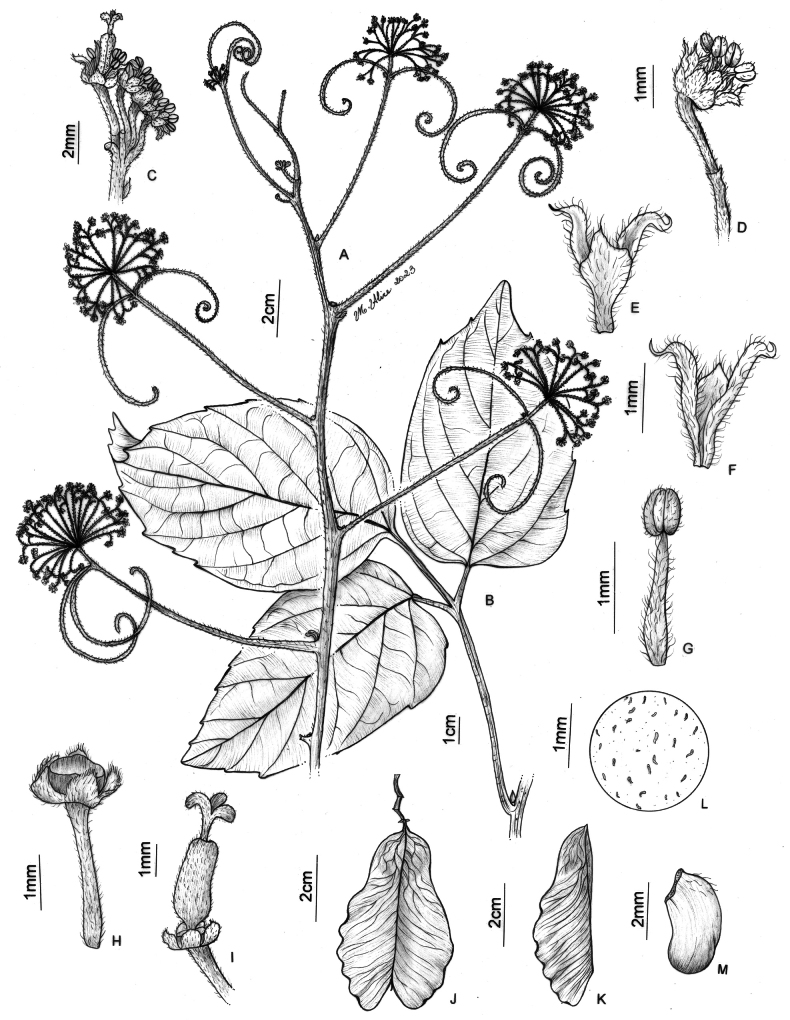
*Thinouiatomocarpa* Standley **A** flowering branch **B** leaf **C** cincinnus with pistillate flowers **D** staminate flower **E–F** petal with bifid appendage, dorsal [abaxial] and frontal [adaxial] views **G** stamen **H** flower with petals removed showing nectary disc **I** young fruit **J** mature fruit **K** mericarp **L** indumentum detail of locule cavity **M** seed (**A–I** from *Manríquez 495***J–M** from *Breedlove 50858*). Illustration by Maria Alice de Rezende.

##### Description.

Tendrilled liana; stems cylindrical, striate, tomentose to glabrescent, lenticels round or elliptical; cross-section simple. Leaves trifoliolate; stipules minute, < 0.5 mm long; petiole ca. 4.8 cm long, terete, puberulous; terminal petiolules 2–2.2 cm long, canaliculate; lateral petiolules 0.8–1 cm long; leaflets glabrous on both surfaces; leaflet secondary venation semicraspedodromous to eucamptodromous, secondary veins 4–6 pairs, alternate, spacing irregular, domatia wanting; intersecondaries present; margins repand-serrate, sometimes only at the apex, with (2) 4–6 teeth on each side, reduced to inconspicuous glands; terminal leaflet 6–8 × 5 cm, elliptic or oblong-ovate, sometimes asymmetrical, the apex long-apiculate or obtuse, the base rounded to obtuse; lateral leaflets 6.5 × 3.9–4.2 cm, elliptic or ovate, sometimes asymmetrical, the apex long-apiculate or obtuse, with a gland at the apex, the base truncate to rounded. Thyrses axillary or terminal, umbelliform, 3.5–6 cm long; peduncle 1.8–3.6 cm long; secondary peduncle sessile or 0.1–0.7 cm long; cincinni numerous, peduncle of cincinnus 2–5 mm long, tomentose. Flower 3–4 mm long, pedicel 1.9–2.4 mm long, tomentose; sepals ca. 0.7 mm long, deltate, abaxially villous and adaxially glabrous; petals 0.6–1.3 mm long, obdeltate, not clawed, villous; appendages 1.4–1.6 mm long, longer than the petals, bifid, marginal, villous; nectary disc annular to slightly lobed, glabrous. Staminate flowers with stamens 8, ca. 4 mm long, the filaments villous throughout, the anthers sparsely villous; pistillode ca. 1 mm long, tomentose. Pistillate flowers with staminodes 8, ca. 2 mm long, villous throughout, the anthers sparsely villous; pistil 1.2–1.4 mm long, villous, the ovary villous, the stigma and style villous. Fruits chartaceous, 3–8 × 1.7–4 cm; accrescent pedicel 2.4–3.8 mm long; stipe ca. 4.3 mm long; seed locule subglobose, but flattened at the base, sometimes slightly flattened, puberulous; seed locule cavity densely covered with capitate trichomes; these with uniseriate stalk and unicellular terminal cells. Seed ellipsoid, 2.5–3.5 × 1.5–2 mm, basally attached.

##### Distribution and habitat.

This species is known from southern Mexico (Chiapas & Veracruz), the Yucatan Peninsula in Mexico (Campeche, Quintana Roo) and Belize and in El Salvador, in tropical and subtropical moist broadleaf forests at 120–830 m elevation (Fig. 19C). Flowering from February to April, and fruiting from April to May.

##### Notes.

*Thinouiatomocarpa* was described by Standley and Record (1936) based on the morphology of the fruit, which appeared as if its apex has been cut off by shears, hence the name *tomocarpa* [from the Greek word *tomus*, meaning ‘cutting’]. This species is otherwise very similar to *T.myriantha*. Although Croat (1976) lumped *T.tomocarpa* in the synonymy of *T.myriantha*, we are resurrecting it because (1) morphologically, *T.tomocarpa* and *T.myriantha* are differentiated by the stamens which are villous throughout in the former but villous only on the lower half in the latter (2) petal appendage is marginal in *T.tomocarpa* (vs. basal); and (3) in our molecular phylogenetic studies show that samples representing *T.tomocarpa* form a monophyletic group with very strong support which is sister to a clade containing *T.myriantha* and *T.silveirae* (Fig. 4).

##### Conservation status.

*Thinouiatomocarpa* possesses an EOO of 227,986.147 km^2^ and an AOO of 56.00 km^2^, with more than 10 known locations. The EOO values and the number of threatening situations approach the thresholds for classifying the species in a threat category. Beyond this, there are no data on population declines for the application of other criteria, thus it should be regarded as Least Concern (LC).

##### Selected specimens examined.

**Belize. Cayo** • Chiquibul Forest 4 km on the road from Las Cuevas, 12 Apr 2003, MacMaster et al. 12 (MEXU). **El Salvador. La Libertad** • Laderas de La Laguna, 6 May 1987, R. Cruz 44 (MEXU). **Mexico. Campeche** • Mun. Calakmul, 7.5 km al W de Flores Magón, 175 m, 12 Mar 2002, Soto et al. 22768 and 22770 (MEXU) • Mun. Hopelchén, a 3 km al N de Zoh-Laguna camino a Dzibalchén, 200 m, 1 Apr 1996, Alvaro and Martínez 268 (MEXU, MO). **Chiapas** • Mun. Ocosingo, Ribera del río Chajulillo al sur de la Estación, 22 Feb 199, Colín 2369 (MEXU) • Mun. Palenque, near side road to Agua Azul 0 km South of Palenque, 13 Apr 1981, Breedlove 50858 (MEXU). **Quintana Roo** • 18 km sobre camino a Tomas Garrido, 8 May 1980, Téllez and Cabrera 2134 (MEXU) • 3 km al sur de La Pantera, por la vía corta a Mérida, 21 Mar 1981, Cabrera 1672 (MEXU) • 12 Km al N de San Felipe Bacalar, 22 Mar 1983, Cabrera 4530 (MEXU, NY) • Mun. Jóse María Morelos, a 11.9 km al SE de La aguada La Presumida, 150 m, 12 Mar 2004, Álvarez et al. 8113 (MEXU) • Mun. Othón P. Blanco, La Pantera, 10 Apr 1998, Granados and Chí 641 (MEXU). **Veracruz** • Mun. San Andrés Tuxtla, Estación de Biología Tropical Los Tuxtlas, 27 Mar 1983, Manriquez 495 (MEXU, NY).

#### ﻿12. Thinouia trifoliolata

Taxon classificationPlantaeSapindalesSapindaceae

[sic, as trifoliata] (Radlk.) Acev.-Rodr. & Ferrucci., Syst. Bot. 42(1): 111. 2017

36CC8528-C164-5DE5-923E-46C62C91CD66

Figs 19C , 23 , 24

Allosanthus trifoliolatus Radlk. in A. Engler, Pflanzenr. [Heft 98f] 4, Fam. 165: 1157. 1933.

##### Type.

**Peru** • Stromgebiet des Maranon, Santiago-Mundung am Pongo de Manseriche, ca. 77°30'W, 1924, *G. Tessmann 4462* (lectotype, here designated: B [2 sheets] [B100673676, B100673675] [image!], syntype: Peru. Stromgebiet des Maranon, Santiago-Mundung am Pongo de Manseriche, ca. 77°30'W, 1924, *G. Tessmann 4444*, B [B10067362] [image!], NY! [NY4206164]).

**Figure 23.**
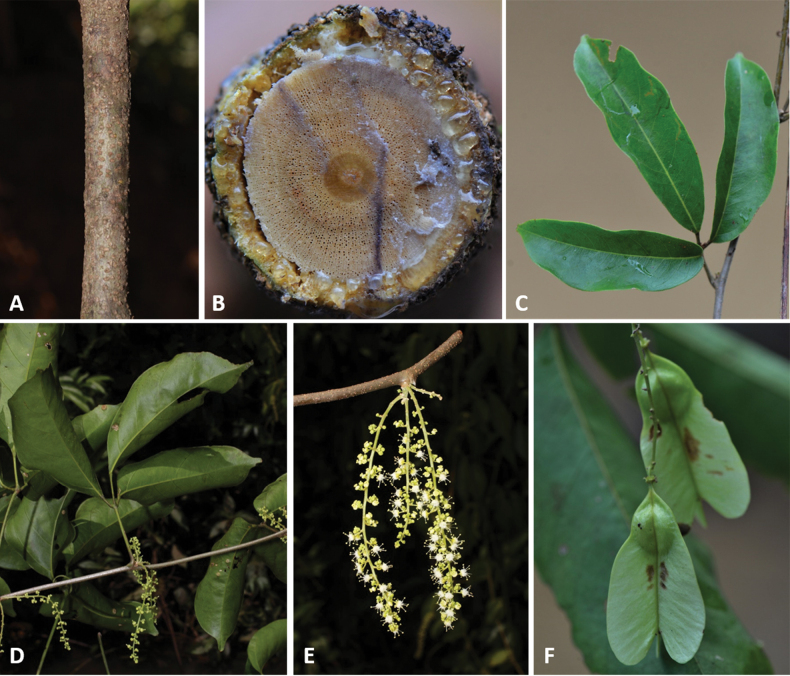
*Thinouiatrifoliolata* (Radlk.) Acev.-Rodr. & Ferrucci **A** stem with lenticellate bark **B** stem cross-section simple **C** leaf **D** fertile branch with axillary inflorescence **E** cauliflorous inflorescence **F** mature fruits [Medeiros 3331 (**A, D, E**); Acevedo-Rodríguez 17159 (**B, C, F**); photos: **B, C, F** by Acevedo-Rodríguez **A, E, F** by H. Medeiros].

**Figure 24.**
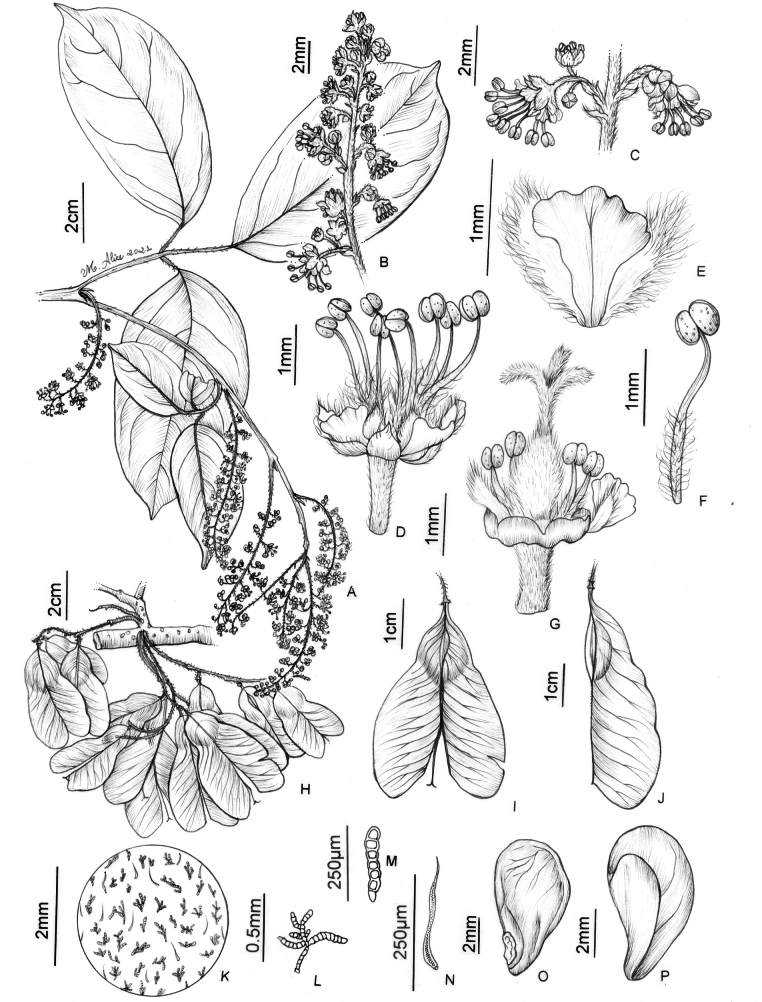
*Thinouiatrifoliolata* (Radlk.) Acev.-Rodr. & Ferrucci **A** flowering branch **B** distal portion of inflorescence **C** portion of inflorescence, showing two cincinni with staminate flowers **D** staminate flower **E** petal with bifid appendage, dorsal [abaxial] view **F** stamen **G** pistillate flower with portion of perianth removed showing nectary disc **H** infructescence **I** fruit **J** mericarp **K** indumentum detail of locule cavity **L** arachnoid trichome of locule cavity **M** capitate trichome with uniseriate stalk and unicellular terminal cells of locule cavity **N** simple trichome of locule cavity **O** seed **P** embryo (**A–G** from *Medeiros 3331***H–P** from *Costa 468*). Illustration by Maria Alice de Rezende.

##### Description.

Tendrilled liana; stem cylindrical, striate, glabrous or puberulous, lenticels round or elliptical, sometimes with whitish or mucilaginous exudate; cross-section simple. Leaves trifoliolate; stipules < 0.5 mm long; petiole 5–7 cm long, terete, glabrous or subglabrous, sometimes pulvinate at base; terminal petiolules 1.6–2.8 cm long, canaliculate, sometimes pulvinate; lateral petiolules 0.7–1.9 cm long, canaliculate, sometimes pulvinulate; leaflets glabrous on the both sides; leaflet secondary venation eucamptodromous, secondary veins 5–8 pairs, alternate or subalternate, spacing irregular, without domatia; intersecondaries present; tertiary veins mixed opposite-alternate percurrent or alternate percurrent; margin entire, sometimes serrate at the apex; terminal leaflet 7–14.5 × 3.4–7.2 cm, elliptic or oblong, symmetrical or asymmetrical, the apex acuminate, rarely rounded to retuse, with an apical gland, the base obtuse; lateral leaflets 6.8–12.5 × 3.3–6.8 cm, elliptic or oblong, the apex acuminate, with an apical gland, the base acute or obtuse. Thyrses cauliflorous, axillary or terminal, racemiform, 4–5.2 cm long; bracts ca. 2.8 mm, linear-lanceolate, pubescent, glabrescent; peduncle 0–0.2 cm long, tomentose; cincinni numerous, peduncle of cincinnus ca. 0.1 cm long, tomentose. Flower ca. 3.5 mm long; pedicel 1.5–2 mm long, glabrous to strigose; sepals ca. 1 mm long, deltate to obdeltate, abaxially strigose or glabrous, adaxially glabrous; petals ca. 1 mm long, obtrullate, not clawed, erose, ciliate along margins; appendages 0.8–1 mm long, shorter than the petal, villous; nectary disc annular, lobed, glabrous. Staminate flowers with stamens 8, ca. 3 mm long, the filaments villous on lower half, the anthers ca. 0.5 long, glabrous, papillose; pistillode ca. 0.5 mm long, villous on the apex. Pistillate flowers with staminodes 8, ca. 1 mm long, villous on lower half; pistil ca. 1.5 mm long, the ovary villous at the apex, the style and stigma villous. Fruits chartaceous, 3–5.1 × 2–2.8 cm; accrescent pedicel ca. 2 cm long; stipe 2–2.5 mm long; seed locule lenticular, sometimes the base flattened; epicarp glabrous; seed locule cavity sparsely ferruginous-pubescent, with simple, capitate and arachnoid trichomes. Seed 7.5 × 4–4.5 mm, obovoid, glabrous.

##### Distribution and habitat.

*Thinouiatrifoliolata* is known from Costa Rica, Colombia, Ecuador, Peru, and Brazil (Fig. 19C), in tropical and subtropical moist broadleaf forests at 100–320 m elevation, in dense and open ombrophilous forests. Flowering from November to February, and fruiting from December to March.

##### Notes.

Unlike most members of the clade in which *T.trifoliolata* emerges (Fig. 4) the inflorescence of *T.trifoliolata* is racemiform. This character is also found in *T.cazumbensis*, a species sympatric with *T.trifoliolata*, so in the absence of molecular data both species could be interpreted as closely related. However, our molecular phylogenetic analyses strongly supports that this character is homoplaseous, i.e., it evolved independently within the clades where these two species belong. Despite sharing a similar inflorescence, *T.trifoliolata* differs from *T.cazumbensis* by its lobed annular disc and sparsely ferruginous pubescent locule cavity (vs. 5-lobbed disc and glabrous locule cavity).

The description of this species was based on two collections (*Tessmann 4444* and *4462*) from the same place and deposited in the same herbarium (B), however the collection *Tessmann 4462* is more representative as it has flowers and complete leaflets. Therefore, we are selecting *Tessmann 4462* as the lectotype.

##### Conservation status.

*Thinouiatrifoliolata* possesses a broad EOO of 3,450,116.37 km^2^ and AOO of 96.00 km^2^, with more than 10 threat situations and records in conservation units. The EOO values and the number of threat situations approach the threshold for the inclusion of the species in a threat category. Thus, *T.trifoliolata* is considered as Least Concern (LC).

##### Selected specimens examined.

**Brazil. Acre** • Mun. Bujari, Riozinho do Andirá, 28 Nov 2013, Costa et al. 373 (LABEV, NY, RB) • Mun. Manoel Urbano, Parque Estadual Chandless, 20 Jan 2014, Costa et al. 468 (LABEV, NY, RB) • Mun. Sena Madureira, Reserva Extrativista do Cazumbá-Iracema, 13 Dec 2019, Medeiros et al. 4380 (NY, RB, SPF, UFACPZ). **Amapá** • Mun. Mazagão, Reserva Genética de Felipe, 10 Oct 1987, Rabelo et al. 3541 (INPA, HAMAB, MO). **Pará** • Mun. Parauapebas. Floresta Nacional de Carajás, 27 Sep 2022, Obermuller et al. 1920 (MG, NY, RB, UPCB). **Colombia. Antioquia** • Rain forest near Río León, 100 m, 20 Mar 1962, Feddema 1983 (MICH, NY, US). **Costa Rica. Puntarenas** • Carara National Park, 30–40 m, Grayum et al. 4720 (MO, NY). **San José** • Carara National Park, 5 Apr 1993, Gentry et al. 79454 (MO). **Ecuador. Pastaza** • Pastaza Cantón, Pozo petrolero Villano 2 de ARCO, 400 m, 1 Dec 1991, Hurtado et al. 2885 (US). **Sucumbios** • Pastaza Cantó, Pozo Reserva Faunística Cuyabero, sendero detrás de estación, tierra firme, 265 m, Apr-Oct 1988, Paz y Miño 81023 (MO). **Peru. Amazonas** • Prov. Bagua, Yamayakat Bosque de Rivera, 320 m, 9 Feb 1996, Jaramillo et al. 1143 (MO, US). **Loreto** • Prov. Requena, Cocha Iricahua, margen izquierda del Río Ucayali, 17 Feb 1982, Encarnación 1298 (NY) • Prov. Ucayali, Canchahuayo, 200 m, 30 Nov 1985, Vásquez and Jaramillo 7058 (MO, NY) • Prov. Maynas, Nauta, quebrada Saragosa, 150 m, 11 Dec 1986, Vásquez and Jaramillo 8577 (F, MO, NY) • Iquitos, Caserio Picuruyacu, 11 Feb 1976, Revilla et al. 127 (MO, USM). **Madre de Dios** • Prov. Tambopata, Puerto Maldonado, 26 Nov 2002, Valenzuela and Huamantupa 1063 (US) • San Martín, Juan Jui, Alto Huallaga, 400–800 m, s.d., Klug 4176 (F).

#### ﻿13. Thinouia ventricosa

Taxon classificationPlantaeSapindalesSapindaceae

Radlk., Sitzungsber. Math.-Phys. Cl. Königl. Bayer. Akad. Wiss. München 8: 282. 1878

C3B73E7A-AA4B-5AEE-BB6D-88372923F661

Figs 19D , 25

##### Type.

**Brazil. São Paulo** • without locality, *C. F. P. von Martius 1303* (lectotype, designated by Massing and Miotto 2020, pg. 160: M [M0212718] [image!]; isolectotype: P [P02297040] [image!].

**Figure 25.**
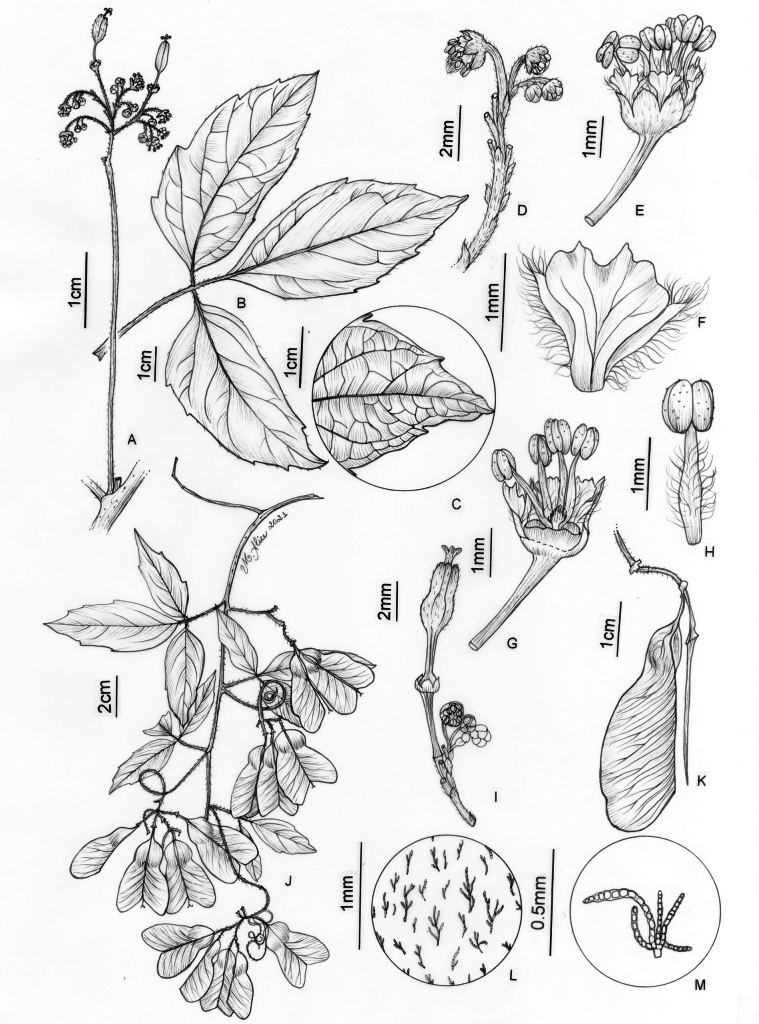
*Thinouiaventricosa* Radlk. **A** axillary inflorescence **B** leaf **C** detail of apex of distal leaflet **D** cincinnus **E** lateral view of staminate flower **F** petal with bifid appendage, dorsal [abaxial] view **G** staminate flower with portion of perianth removed showing nectary disc and pistillode **H** stamen **I** cincinnus with young fruit **J** branch with fruits **K** mericarp **L** indumentum detail of locule cavity **M** arachnoid trichomes of locule cavity (**A, D–I** from *Rosado 222***B, C** from *Hatschbach 19194* and **J–M** from *Caxambu 5920*). Illustration by Maria Alice de Rezende.

##### Description.

Tendrilled liana; stem cylindrical, striate, puberulous, with round or elliptical lenticels; cross-section simple or with neo formations when mature. Leaves trifoliolate; stipules ca. 0.3 mm long, triangular, glabrous or puberulous; petiole 1.5–5.3 cm long, terete or subterete, puberulous or pubescent; petiolules canaliculate, terminal petiolules ca. 0.6 cm long, lateral petiolules 0.2–0.4 cm long, rarely subsessile; leaflets glabrous on both sides or sometimes abaxially pubescent; leaflet secondary venation semicraspedodromous, secondaries 4–5 pairs, alternate, spacing irregular, with domatia on abaxial surface of secondary vein axils, frequently only on the lowermost pair of secondary veins, rarely on other secondaries; intersecondaries presents; tertiary veins irregular reticulate; margins entire to serrate, with 2–3(4) teeth reduced to inconspicuous glands; terminal leaflet 4.7–7.6 × 1.8–2.8 cm, lanceolate, symmetrical or asymmetrical, the apex acute to acuminate, mucronate, the base acute to decurrent, rarely obtuse; lateral leaflets 3.9–7 × 1.5–2.8 cm, lanceolate, asymmetrical, the apex acute to acuminate, mucronate, the base decurrent or rounded. Thyrses axillary or terminal, umbelliform, 2.4–4.5 cm long, peduncle 1–5 cm long, secondary peduncle (0)0.1–0.2 cm long; cincinni numerous, peduncle of cincinnus 0.7–2 mm long. Flowers 2.6–4 mm long, pedicel 2–2.5 mm long, glabrous; sepals ca. 0.5 mm long, connate at the base, deltate, abaxially puberulent, adaxially glabrous, villous at margins; petals ca. 1.4 mm long, short spatulate, abaxially villous on the central part, the rest glabrous; petal appendages rudimentary, bifid, smaller than the petals, villous; nectary disc annular, glabrous, Staminate flower with stamens 8, ca. 3 mm long, filaments villous for little more than half of their length, anthers papillose, glabrous or sparsely pubescent; pistillode ca. 0.9 mm long, pilose on distal half. Pistillate flower with staminodes ca. 1.2 mm long, villous; pistil ca. 1.3 mm long, pilose on distal half, ovary ca. 1 mm long, style ca. 0.2 mm long. Fruits chartaceous, 3.4–4.7 × 1.8–2.5 cm; accrescent pedicel 5–6.1 mm long; stipe 6.3–8.1 mm long; seed locule lenticular; epicarp glabrous to subglabrous; cavity of seed locule villous, with arachnoid, biseriate, or simple trichomes. Seed ellipsoid, ca. 5.8 × 4.4 mm, basally attached, glabrous.

##### Distribution, habitat and phenology.

*Thinouiaventricosa* is known from tropical and subtropical moist broadleaf forests in the states of Paraná, Rio Grande do Sul, Santa Catarina, and São Paulo, Brazil, and from Argentina (Fig. 18D), in semi-deciduous and ombrophilous forests. Flowering from December to February, and fruiting January to August.

##### Notes.

*Thinouiaventricosa* can be confused with *T.restingae* (see comments under *T.restingae*). Additionally, *T.ventricosa* is similar to *T.scandens* due to the great morphological variability of leaves and fruits. However, *T.ventricosa* is easily distinguished from the latter species by its smaller flowers (2.6–4 mm long vs. 4.5–6 mm long).

##### Conservation status.

*Thinouiaventricosa* possesses a broad EOO of 306,232.35 km^2^ and an AOO of 92.00 km^2^, with few known locations. However, the semi-deciduous and ombrophilous forests in Southeastern Brazil and Northeastern Argentina, where the species is found, are subject to continuing decline in area and quality of habitat due to anthropic pressure. Despite this, there are no data on population declines to apply other criteria, and several conservation units in Brazil and Argentina protect the species. Thus, it should be regarded as Least Concern (LC).

##### Selected specimens examined

**. Argentina. Misiones** • El Dorado, Km 31 ruta nac. 12, 10 Jul 1972, Schinini 4904 (MBM). **Brazil. Paraná** • Mun. Cianorte, Estrada Cambuci, 5 Apr 2012, Rosado 59 (HUEM) • Mun. Engenheiro Beltrão, Reserva Florestal de Figueira D’Oeste, 19 Mar 2007, Grande s. n.. (MBM 342283) • Mun. Fênix, RPPN Vila Rica, 4 Mar 2015, Caxambu et al. 5920 (HCF) • Mun. Guarapuava, Parque Municipal das Araucárias, 8 Jan 2005, Cordeiro 176 (MBM, ESA) • Mun. Maringá, Parque das Palmeiras, 16 Aug 2019, Rosado 969 (HUEM) • Mun. Morretes, Fartura, 29 Jan 1980, Hatschbach 42750 (F, INPA, MBM, MO, MU, RB, UEC, US, WAG) • Mun. Nova América da Colina, Rio Água Três Barras, 18 May 1998, Francisco et al. s. n.. (MBM 338062) • Mun. Rio Bonito do Iguaçu, Fazenda Giacmet-Marodin, 23 Jun 1995, Poliquesi 347 (MBM, MEXU) • Mun. Rio Branco do Sul, Ribeirinha, 7 May 1968, Hatschbach 19194 (F, MBM, NY, P, UPCB, US) • Mun. Sarandi, Condomínio Estância Zaúna, 31 Jul 2019, Rosado 947 (HUEM). **Rio Grande do Sul** • Mun. Derrubada, 15 Aug 2018, Massing 323 (ICN) • Mun. Santa Rosa, 15 Jul 1967, Hagelund 5408 (ICN) • Mun. Tenente Portela, Parque Estadual do Turvo, s.d., Brack et al. 1758 (ICN). **Santa Catarina** • Mun. Porto União, São Miguel, 12 Jul 1962, Reitz et al. 13110 (HBR) • Mun. Rio do Sul, Perto da Cidade, 8 Jul 1964, Reitz et al. 17062 (HBR). **São Paulo** • Without locality, s.d., Helmreichen 58 (F, HUH, NY, WAG) • Mun. Campinas, Prope Campinas, s.d., Mello s. n.. (P06695482) • Mun. Itaporanga, Rio Verde, 17 Jun 1990, Hatschbach et al. 54328 (MBM, US) • Mun. Mombuca, Mata do Pinheirinho, 26 Jun 2021, Medeiros and Toledo 4493 (ESA, RB, SPF) • Mun. São Carlos, São Carlos do Pinhal, Jul 1888, Loefgren 705 (SP) • Mun. São Paulo, Nativa do Jardim Botânico, 6 Jan 1940, O. Handro s. n.. (SP, US01319028).

## ﻿Discussion

### ﻿Phylogenetic relationships

The phylogeny presented here meets the main objective of this study, which was to enhance the understanding of the phylogenetic relationships among *Thinouia* species. Furthermore, it was possible to confirm previous phylogenetic studies that supported the monophyly of *Thinouia*, with *Thinouia* being the sister group to the rest of Paullinieae (Acevedo-Rodríguez et al. 2017; Chery et al. 2019; Medeiros et al. 2020; Cunha Neto et al. 2023). This study provides a baseline for understanding the genus *Thinouia* and the evolution of the tribe Paullinieae. We then discuss the main clades recovered by the phylogenetic analyses and compare them with the infrageneric classification proposed by Radlkofer (1878).

The results of this study support *Thinouia* as monophyletic and indicate *T.cazumbensis* as the sister to all other species of the genus, which splits into two clades (Clade II and III). However, the divergence into the two clades is poorly supported. Clade II is widely distributed in southern Mexico, Central America, and northern South America. In all three of its species (*T.myriantha*, *T.silveirae* and *T.tomocarpa*) the petal appendages are longer than the petals, a character used by Radlkofer (1878) to define section Lepidodine. However, the shape of petal appendages of these species is different. *Thinouiamyriantha* has petal appendages adnate to the basal portion, while in *T.tomocarpa* and *T.silveirae* the petal appendage is adnate to the marginal portion.

Clade III includes *Thinouiacompressa*, *T.mucronata*, *T.obliqua*, *T.paraguayensis*, *T.restingae*, *T.scandens*, *T.ternata*, *T.trifoliolata*, and *T.ventricosa* (PP = 1.0). This clade included species that are almost exclusively distributed in South America with the exception of *T.trifoliolata*, which occurs in lowland rainforests of the Amazon region and Central America. All species in this clade have petal appendages that are smaller than or equal in size to the petals. This character was used by Radlkofer (1878), to define section Petalodine, but as this character is also found in *T.cazumbensis*, it cannot be used as a morphological descriptor for sect. Petalodine since it seems to be a symplesiomorphic character.

Within tribe Paullinieae, small petal appendages are found in all other genera, but *Thinouia*, which is sister to the rest of the tribe, is the only genus whose petal appendage character was used in an infrageneric classification. Despite the great morphological variation found in petal appendages in Sapindaceae, especially in the tribe Paullinieae (Radlkofer 1895), few studies compare or elucidate the evolutionary changes of petals and petal appendages in Sapindaceae (Leinfellner 1958; Endress and Matthews 2005).

In conclusion, considering that only one of the *Thinouia* sections defined by Radlkofer (1878) is monophyletic, and that there is not enough data at the moment to propose an infrageneric classification, in *Thinouia* we propose to refrain from using one.

## Supplementary Material

XML Treatment for Thinouia

XML Treatment for Thinouia cazumbensis

XML Treatment for Thinouia compressa

XML Treatment for Thinouia mucronata

XML Treatment for Thinouia myriantha

XML Treatment for Thinouia obliqua

XML Treatment for Thinouia paraguayensis

XML Treatment for Thinouia restingae

XML Treatment for Thinouia scandens

XML Treatment for Thinouia silveirae

XML Treatment for Thinouia ternata

XML Treatment for Thinouia tomocarpa

XML Treatment for Thinouia trifoliolata

XML Treatment for Thinouia ventricosa

## ﻿Additional information

### Author ORCIDs

Herison Medeiros https://orcid.org/0000-0001-9785-6777

Pedro Acevedo-Rodríguez https://orcid.org/0000-0002-0946-8668

Jenifer de Carvalho Lopes https://orcid.org/0000-0001-6485-9023

Rafaela Campostrini Forzza https://orcid.org/0000-0002-8366-0738

### Data availability

All of the data that support the findings of this study are available in the main text.

## ﻿Appendix 1

**Index to numbered collection studied**

The species epithet comes inside parentheses. The collection numbers for vouchers without collector´s numbers are provided within square brackets with acronym herbarium.

Abbott, J.R. 16326 (paraguayensis).

Abruzzi, M.L. 655 (mucronata).

Acevedo-Rodríguez, P. et al. 1436, 3697 (scandens); 4539, 4578 (mucronata); 7577 (obliqua); 8788, 12359, 14262, 14596, 15037, 17080 (myriantha), 16750 (paraguayensis).

Agra, M.F. 6985 (compressa).

Álvarez, D. 8113 (tomocarpa).

Alvaro, M.P. and Martínez, E. 268 (tomocarpa).

Alves, R.J.V. 4981 (compressa).

Amorim, A.M. 3580 (ternata).

André, Ed. 1841 (myriantha).

Alves, L.M.T. 7 (mucronata).

Anderson, R. 9187 (compressa).

Anderson, W.R. 36988 (compressa).

Araújo, A. 423 (compressa).

Araújo, D. 10025 (restingae).

Armond, N. [R19595] (compressa).

Aulestia, M. 3367 (obliqua).

Baker, M.A. 5993 (obliqua).

Balansa, B. 2486 (paraguayensis); 2488 (mucronata); 36945 (compressa).

Barbosa, E. 1448 (mucronata); 1544 (paraguayensis).

Barreto, K.D. 2010 (mucronata).

Barreto, M. 7558 (compressa).

Bastos, F.M. and Neves, L.F.F. F5 (mucronata).

Baylão, Jr., H.F. 367 (scandens).

Beck, St. G. 13309 (myriantha).

Belém, R.P. 3597 (ternata).

Belshaw, C.M. 3176 (obliqua).

Bianek, A.E. 172, 229 (mucronata).

Bonaldi, R. 506, [MBM375789], [MBM375790] (mucronata);

Borges, R.A.X. 224 (ternata).

Bovini, M.G. 170, 1925 (restingae); 3824 (compressa).

Bowie, J. and Cunningham 254 (scandens).

Brack, P. et al. 1197 (mucronata); 1758 (ventricosa);

Brade, A.C. 7923 (scandens).

Braga, H.N. 701 (restingae).

Braun, B.K. PSACF_EX05036 (myriantha);

Breedlove, D.E. 50858 (tomocarpa).

Bueno, R. [ICN2805], [ICN2808] (mucronata).

Burnham, R.J. 1569 (obliqua); 1671 (myriantha).

Bunting, G.S. 8744 (myriantha).

Cabrera, A.L. 22085, 31483 (mucronata).

Cabrera, E. 1672, 4530 (tomocarpa).

Calió M.F.A. 70 (compressa).

Campos, M.D. 14 (scandens).

Campos, V.A. 5540 (tomocarpa).

Campos Novaes, J. 3202 (mucronata).

Cardoso, D. 1245 (ternata).

Carneiro, J.S. 118 (mucronata).

Carvalho, A.M. 2489 (scandens).

Carvalho, W.B. 265 (scandens).

Catharino, E.L.M. 288 (mucronata).

Caxambu, M.G. 176, 5920 (ventricosa); 304, 306, 2524, 5215, 6486, 6559 (mucronata); Cerón, C.E. 3657, 5453 (obliqua).

Ceteno, P. and Janovec, J.P. 155 (myriantha).

Chagas, F. and Silva 1501 (mucronata).

Churchill, H.W. and Nevers, G. 4441 (myriantha).

Clarke, D. 3964 (myriantha).

Clark, J.L. 6466 (obliqua).

Claudio, 454 (paraguayensis).

Claussen, M. 521 (mucronata).

Colín, S.S. 2369 (tomocarpa).

Colleta, G.D. and Flores, T.B. 520 (scandens).

Colman, L.P. 21 (scandens).

Cordeiro, I. 176 (ventricosa); 834 (mucronata).

Correa de Mello 7 (ventricosa).

Costa, D.S. 359, 756 (myriantha); 373, 468 (trifoliolata); 486 (obliqua).

Coulleri, J.P. 384 (mucronata).

Couto, D.R. 1131 (scandens).

Cruz, R. 44 (tomocarpa).

Daly, D.C. 6570, 9727 (obliqua); 10444 (myriantha).

Daneu, L. 746 (ternata).

Daniel [RBR 32855] (mucronata).

Damasceno Júnior, A.G.1518, 2186 (paraguayensis).

De La Rue, E.A. [P0669512] (myriantha).

Duarte, A.P. 4966 (scandens).

Ducke, A. [INPA12163] (compressa).

Dugand, A. 872 (myriantha).

Durigon, J. 456, 930 (mucronata); 607 (compressa); 977 (trifoliolata).

Ekman, L. 1514 (mucronata).

Elias, Bro. 1266, 1412 (myriantha).

Encarnación, F. 1298 (trifoliolata).

Farney, C. et al. 2173 (restingae).

Feddema, C. 1983 (trifoliolata).

Fernandes, D.S. 195, 361, 855 (restingae).

Ferrucci, M.S.178, 2535, 3128 (paraguayensis); 2726 (mucronata).

Ferrucci, S. 161, 179 (mucronata).

Florschütz, P.A. and Maas, P.J. 255 (myriantha).

Fiaschi, P. 755 (scandens); 1395 (mucronata).

Fiebrig, K. 506, 911 (paraguayensis); 5859, 5978 (mucronata).

Figueira, M. 927 (mucronata);

Folli, D.A. 1021, 1076, 3910, 4162, 4740, 7529 (scandens).

Forzza, R.C. et al. 5089, 5147, 7815 (restingae).

Foster, R. 2204, 5905 (myriantha).

Fraga, C.N. 2083 (scandens).

Francisco, E.M. [MBM338060], [MBM338061] (mucronata); [MBM338062] (ventricosa).

França, F. 3507 (mucronata); 4971 (compressa).

Frazão, A. 417, 418 (myriantha).

Fróes, R.L. 23958, 24586 (myrianhta).

Fuentes, A.C. 1484 (paraguayensis).

Gentry, A.H. 58605 (obliqua); 78338, 78457, 79370 (myriantha); 79454 (trifoliolata).

Giordano, L.C. 1032 (scandens).

Glaziou, A. 857, 3897, 3898, 3899, 3902, 5773, 6864, 8599, 15449, 17499, 18170 (scandens).

Gonçalves, A.C. PSACF_EX04702, PSACF_EX04844 (myriantha).

Gonçalves, M.I.T.M. [RB00594985] (scandens).

Granados, J. and Chí, F. 641 (tomocarpa).

Grande, F. [MBM342283] (ventricosa).

Grayum, M. 4720 (trifoliolata).

Grijalva, A. 450 (myriantha).

Grings, M. 315, 346, 1272 (mucronata).

Gutiérrez J. 1200 (mucronata).

Hagelund, K. 2418, 3678, 3707, 4947, 5243, 8273 (mucronata); 5408 (ventricosa).

Hahn, W. 1072, 1506 (mucronata).

Harley, R.M. 21701, 21997 (compressa).

Hassler, E. 1846, 3745, 8297, 11514 (paraguayensis); 4460 (mucronata).

Hatschbach, G. 15753, 19360, 21007, 21527, 46203, 50380, 60563, 76943 (mucronata); 19194, 42750, 54328 (ventricosa); 49244 (paraguayensis); 55100 (ternata); 65076, 67777, 74746 (compressa).

Haught, O. 3867, 4746 (myriantha).

Heringer, E.P. 725 (compressa).

Hoehne, F.C. 3969, 3971 (paraguayensis); [SP46367] (scandens).

Hoffman, B. 5308, 6091 (myriantha).

Holton, I.F. [NY04219069] (myriantha).

Huamantupa, I. and Carrión, L. 9075 (myriantha).

Hurtado, F. 2885, 3013 (trifoliolata).

Irwin, H.S. 23129 (scandens); 23219 (ternata).

Jacques, E.L. 91 (scandens).

Jansen-Jacobs, M.J. 4314 (myriantha).

Jaramillo, N. 1143 (ventricosa).

Jarenkow, J.A. 2343 (mucronata).

Kaprovickas, A. 39403, 44016 (mucronata).

Karhs and Schenkel [ICN2810] (mucronata).

Karsten, H. [NY04219067] (myriantha).

Kegler, A. 153 (mucronata).

Keller, H.A. 144, 622, 6919 (mucronata).

Kernan, C. 867 (myriantha).

Kessler, M. 3983 (compressa).

Killeen, T. 3825 (obliqua).

Killip, E.P. and Smith, A.C. 22969, 26139 (obliqua).

Kinnup, V.F. 217, [FUEL27790] (mucronata).

Kirizawa, M. 756 (mucronata).

Klein, O.M. [MBM 075952] (mucronata).

Klein, R.M. 8690 (mucronata).

Klug, G. 4058, 4102, 4293 (obliqua); 4176 (trifoliolata).

Kollmann L. 11067 (compressa).

Kozeira, C. and Ribeiro, A. 3716, 3718 (mucronata).

Kozera, C. and Cardozo, A.L. 3770 (mucronata).

Knapp, S. et al. 7405 (obliqua).

Kuhlmann [RB 78342] (mucronata).

Kuhlmann, M. 610 (mucronata).

Kuntze, O. [NY04219055] (mucronata); [NY04219094] (paraguayensis).

Laclette, P. 166, 751 (scandens).

Leoni, L.S. 63, 2134, 3950, 7575 (scandens).

Lima, H.C. 3501 (scandens).

Lindman, C.A.M. 1063 (mucronata).

Lindman, J.C. and Haas, J.H. 4254, 5325 (mucronata).

Linneo, I. and Carreño 1405a (obliqua).

Lise, A. 5860 (mucronata).

Löfgren, A. 625 (compressa), 705 (ventricosa).

Lopes, M.M.M. 1338 (compressa).

Lovato, M.C. [RBR 36323] (mucronata).

Lozano, R. 2056, 2128 (mucronata).

Maceda, A. 1097 (myriantha).

Macía, M.J. 4542 (myriantha).

MacMaster, G. 12 (tomocarpa).

Maguanini A. [RB87950] (scandens).

Maihack, R. [RB613850] (mucronata).

Malme, G.O. 2748 (paraguayensis).

Manriquez, G.I. 495, 3365 (tomocarpa).

Manriquez, G.I. and Sinaca, S.C. 2369 (tomocarpa).

Mansano, V.F. 480 (restingae).

Manzine, F.F.415 (mucronata).

Marques, J.P. [HCF5351] (mucronata).

Marquete, R. 351, 3501 (scandens); 1692 (mucronata); 1826 (restingae).

Martínez, E.S. 18295, 11916 (tomocarpa).

Massing, A.A. 125, 224 (mucronata); 323 (ventricosa).

Mathias, M.E. and Taylor, D. 3479, 3980, 3991, 5395 (obliqua).

Mauad, L.P. 119 (restingae).

Medeiros, H. 2189, 2191, 2193, 4496, 4498 (silveirae); 3328, 3332, 3792, 3810, 3832, 4884 (obliqua); 3330, 3788, 4237, 4300, 4898 (myriantha); 3331, 4380 (trifoliolata); 3401 (cazumbensis); 3800 (compressa); 4450, 4451, 4452, 4472, 4473, 4481, 4486, 4488 (scandens); 4475, 4453, 4491 (restingae), 4489 (ternata); 4493 (ventricosa).

Mello, de C. [P06695482] (ventricosa).

Mello-Silva, R. 1292 (compressa).

Melo, P.H.A. 625 (ternata).

Menescal, A.L. 103 (mucronata).

Mereles, F. 3842 (paraguayensis).

Mexia, Y. 4996 (compressa); 6675 (obliqua).

Mikan 5566 (scandens).

Miranda, E.B. 706 (compressa).

Molina, J.A. 19Bo022, 19At075 (myriantha).

Montes, J.E. 100B, 1871, 2430, 2551 (mucronata).

Moore, S. 1076 (paraguayensis).

Mori, S.A. 10061 (mucronata); 14419, 14426 (compressa).

Morong, T. 625a (paraguayensis).

Mosén, H. 3953 (mucronata).

Mostacedo, B. 3637 (paraguayensis).

Nascimento, E.A.P. 33 (myriantha).

Nee, M. et al. 37644, 44866, 48561 (paraguayensis); 45374, 48052, 53120, 55032 (mucronata).

Nee, M. and Bohs, L. 49447 (mucronata).

Obermuller, F.A. 1920 (trifoliolata).

Ochioni, P. [RB2882] (trifoliolata).

Oliveira, J.E. 387 (compressa).

Ortíz, M. 711 (paraguayensis).

Paixão, J.L. 211 (restingae); 909 (scandens).

Palacios, W. 11034 (myriantha).

Pastore, J.A. 514 (mucronata).

Paula-Souza, J. and Souza V.C. 5694 (scandens).

Pauleiro and Vitorio [RB148994] (scandens).

Pavão, O.C. [RB422905] (mucronata);

Paz, G. and Miño 81023, 81043 (trifoliolata).

Pedersen, T.M. 11042 (mucronata).

Peixoto, A. 4124 (scandens).

Peredo, I. 22 [NY04219096] (paraguayensis).

Pérez B. 68 (paraguayensis).

Philipson, W.R. 2197 (myriantha).

Pirani, J.R. 482, 488 (mucronata); 3758 (compressa).

Pittier, H. 8758 (myriantha).

Pohl 3763, 5566 (scandens).

Poliquesi, C.B. 347 (ventricosa).

Prance, G.T. 26298 (paraguayensis).

Proença, M. 82, 89, 115, 123 (mucronata).

Pujals, A. [MBM420905], [MBM420916] (scandens).

Queiroz, L.P. 2472 (scandens); 9439 (ternata); 12715 (compressa).

Rabelo, B.V. 3541 (trifoliolata).

Rapini, A. 628 (compressa).

Reitz 13110, 17062 (ventricosa);

Revilla, J. 127 (trifoliolata).

Riedel, L. 513 (compressa); [US01318766] (scandens).

Rodriguea C.D.N. 133 (mucronata).

Rodrigues, I.A. 1632 (myriantha).

Rodríguez 715 (mucronata).

Rojas, R. 1147 (5).

Romagnolo, M.B. 3313 (trifoliolata).

Rombous, H.E. 669 (myriantha).

Romoleroux, K. and Baus, E. 3200 (obliqua).

Rosado 59, 222, 223, 224, 947, 969 (ventricosa); 371 (mucronata).

Rose, J.N. 19992 (compressa); 20073 (mucronata).

Rusby, H.H. 550 (compressa).

San´t Ana, S.C. 291 (scandens).

Saint-Hilaire [MPU010891] (scandens).

Saldanha, J. 5513 (scandens).

Salino, A. 3951 (scandens).

Salmazi, L.B. [RBR 32856] (mucronata).

Santos, M.C.F. 150 (scandens).

Saraiva, C. and Johnson, D. 42 (myriantha).

Saucedo, M. 836 (paraguayensis).

Schinni, A. 4904 (ventricosa); 22301, 27933 (mucronata).

Schipp, W.A. 1336 (tomocarpa).

Schott, H.W. 4421 (scandens).

Schunke, J.M. 183 (myriantha).

Schunke-Vigo, J. 4104, 12520, 14868 (obliqua); 6533 (myriantha).

Schwacke 7085 (scandens).

Schwarz, G.J. 7399 (mucronata).

Schwindt, E. 3087 (mucronata).

Sekine, E.S. 97 (mucronata).

Sellow, F. [NY 04219107] (scandens).

Silva, M.F.B. 122 (scandens).

Silva, M.F.F. 169 (myriantha).

Silva, N.T. 1022 (cazumbensis).

Silveira, A.L.P. 500 (myriantha).

Siqueira, E.L. 204, 1385, 2162 (mucronata).

Smith, H.H. 882 (myriantha).

Soares-Silva, L.H. 26 (mucronata).

Solomon, J.C. 9966 (mucronata).

Somner, G.V. 544 (ternata); 585, 1541, 1851, 1339 (scandens); 616, 752, 785, 788, 801, 1074, 1185, 1354, 1495, 1496 (restingae).

Sonia, N. 2083 (paraguayensis).

Sork, H.E. et al. 10496 (obliqua).

Soto, J.C. 22768, 22770 (tomocarpa).

Souza, de V. 439 (ternata).

Souza, V.C. 10426 (mucronata); 32487 (restingae).

Stehmann, J.R. [MBM227949], 2959 (compressa).

Stevenson, P. 2144 (obliqua).

Steyermark, J.A. 38774, 93135 (myriantha).

Stival-Santos, A. 588 (mucronata).

Stutz, L.C. 2331 (mucronata).

Urdampilleta, J.D. 118, 148 (mucronata).

Tamashiro, J.Y. 1015 (mucronata).

Téllez, O. and Cabrera, E. 2134 (11).

Tessmann, G. 3314 (myriantha); 3500 (obliqua); 4444, 4462 (trifoliolata).

Torke, B.M. 2024 (myriantha).

Tressens, S.G. 4409 (mucronata); 6164 (myriantha).

Triana, J. [MPU010893, MPU010892], [P04857748], [P06695510], [BM000838111] (myriantha).

Trigos, R.C. and Sinaca, S.C. 2682 (tomocarpa).

Uittien, H. 6988 (myriantha).

Valenzuela, L. and Humantupa, I. 1063 (trifoliolata).

van Andel, T.T 4485 (myriantha).

Vanni, R. 3621, 3939, 4179, 4518 (mucronata).

Vásquez, R. 14290 (myriantha).

Vásquez, R. and Jaramillo, N. 7058, 8577 (trifoliolata).

Victorio [RB003386344] (scandens).

Vieira, C.M. and Gurken, L.C. 429 (scandens).

von Martius, C.F.P. 1303 (ventricosa).

Zárate, R. 14005 (myriantha).

Zardini, E.M. 5433, 5651, 9207 (paraguayensis); 13513, 42893 (mucronata).

Zardini, E. and Aquino, P. 29700 (compressa); 33097 (paraguayensis).

Zardini, E.M. and Benítez, C. 3333 (compressa).

Zardini, E.M. and Chaparro, I. 48588, 48847 (mucronata).

Zardini, E.M. and Garcete, F. 43321 (compressa).

Zardini, E.M. and Guerrero, L. 48472 (compressa); 59086 (mucronata).

Zardini, E.M. and Soria, N. 4448 (paraguayensis).

Zardini E. and Tilleria 29772 (mucronata).

Zardini, E.M. and Velásquez, C. 12165, 13669 (compressa); 12324 (mucronata); 13053, 18791, 22320 (paraguayensis);

Zardini, E.M. and Vera, M. 45362 (compressa).

Zeiden, D.N.M. 34 (mucronata).

Warming [P06695484] (ternata).

Woolston, A.L. 664, 840, 850 (compressa); 784 (mucronata); 1248 (paraguayensis).

Woytkowski, F. 5065, 7153 (obliqua).

Wurdack, J.J. and Monachino, J.V. 38715 (myriantha).

## ﻿Appendix 2

**Voucher and GenBank information for the taxa included in the phylogenetic analyses**

Listed as: taxon, collection, herbarium, place of origin and GenBank accession numbers (ITS, trnL intron). Herbarium acronyms follow Index Herbariorum (Thiers 2022).

*Lophostigmaplumosum* Radlk., Acevedo-Rodríguez 6554 (US), Bolivia, KX584929, KX585020. *Paulliniacuneata* Radlk., Acevedo-Rodríguez 14255 (US), Peru, KX584932, KX585023. *Paulliniaelegans* Cam­bess., Acevedo-Rodríguez 14976 (US), Brazil, KX584933, KX585024. *Serjaniacaracasana* (Jacq.) Willd., Acevedo-Rodríguez 15107 (US), Mex­ico, KX584947, KX585038. *Serjaniacommunis* Cambess., Somner 1334 (US), Brazil, KX584950, KX585041. *Thinouiacazumbensis*, Medeiros 3401 (RB) Bra­zil, MT853074, MT847016. *Thinouiacompressa* Radlk., Medeiros 3800 (RB) Brazil, MT853076, MT847018. *Thinouiamucronata* Radlk., Keller 6919 (US), Argen­tina, KX584971, KX585058. *Thinouiamucronata* Radlk., Nee 48052 (US), Bolivia. *Thinouiamyriantha* Radlk., Torke 2024 (HSTM), Brazil, MT853071, MT847013. *Thinouiaobliqua* Radlk., Medeiros 3793 (RB) Bra­zil, MT853075, MT847017. *Thinouiaparaguayensis* (Britton) Radlk., Barbosa 1544 (HUEFS), Brazil. *Thinouiaparaguayensis* Nee 48561 (US), Bolivia. *Thinouiarestingae* Ferrucci & Somner, Somner 1074 (RBR), Brazil, KX584972, KX585060. *Thinouiarestingae* Ferrucci & Somner, Medeiros 4453 (RB), Brazil. *Thinouiascandens* (Cambess.) Triana & Planch., Medeiros 4472 (RB), Brazil. *Thinouiascandens* (Cambess.) Triana & Planch., Medeiros 4488 (RB), Brazil. *Thinouiascandens* (Cambess.) Triana & Planch., Somner s. n.. (RBR), Brazil. *Thinouiasilveirae*, Medeiros 2193 (RB), Brazil, MT853072, MT847014. Brazil. *Thinouiaternata* Radlk., Cardoso 1245 (RB), Brazil. *Thinouiatomocarpa* Standley, Acevedo-Rodríguez 12238 (US), Mexico. *Thinouiatomocarpa* Standley, Soto 22768 (MEXU), Mexico. *Thinouiatomocarpa* Standley, MacMaster 12 (MEXU), Belize. *Thinouiatrifoliolata* Acev.-Rodr. & Ferrucci, Rabelo 3541 (MO), Brazil. *Thinouiatrifoliolata* Acev.-Rodr. & Ferrucci, Revilla 127 (MO), Peru. *Thinouiaventricosa* Radlk., Ferrucci 1990. *Thinouiaventricosa* Radlk., Grande s. n.. (MBM 342283). *Thouiniaacuminata* S. Watson, Liston 633-2, ––, EU720478, EU721249.
